# Effect of HIIT on hemostasis and vascular stiffness: a systematic review and meta-analysis of randomized controlled trials

**DOI:** 10.3389/fcvm.2025.1573100

**Published:** 2025-07-08

**Authors:** Mingyue Jiao, Qingmei Li, Xianzhi Xie, Zhen Qiu

**Affiliations:** ^1^ School of Teacher Education, Hezhou University, Hezhou, Guangxi, China; ^2^ School of Tourism and Sports Health, Hezhou University, Hezhou, Guangxi, China

**Keywords:** HIIT, hemostasis, vascular stiffness, systematic review, meta-analysis

## Abstract

**Background:**

Several small randomized trials have examined the effects of high-intensity interval training (HIIT) on hemostasis and vascular stiffness. However, a clear consensus regarding these effects has not been established. The study is to systematically review the evidence and quantify the impacts of HIIT compared with moderate-intensity continuous training (MICT) or usual care (UC) on hemostasis and vascular stiffness.

**Methods:**

Randomized controlled trials (RCTs) exploring the impact of HIIT, MICT, or UC on hemostasis and vascular stiffness were retrieved from PubMed, Embase, Web of Science, and Cochrane databases up to June 10, 2025. A meta-analysis was performed to compare the standardized mean differences (SMD) of changes from baseline to post-intervention in platelet count (PLT), fibrinogen (FIB), D-dimer (D-D), carotid-femoral pulse wave velocity (cfPWV), augmentation index (AIx), AIx normalized to a heart rate of 75 beats·min^−1^ (AIx@75HR), flow-mediated dilation (FMD), and brachial flow-mediated dilation normalized (nFMD), which were used to evaluate hemostasis and vascular stiffness. The 95% confidence interval (95% CI) was calculated along with the SMD. All analyses were conducted in R (version 4.3.3).

**Results:**

Overall, 68 RCTs involving 2,679 patients were included in the analysis. PLT [SMD (95% CI) = −0.26 (−0.51; −0.01)] and FIB [SMD (95% CI) = −0.60 (−1.18; −0.01)] in hemostasis were decreased. Decreased cfPWV [SMD (95% CI) = −0.22 (−0.38; −0.06)], AIx [SMD (95% CI) = −0.16 (−0.30; −0.02)], and AIx@75HR [SMD (95% CI) = −0.35 (−0.61; −0.10)], as well as increased FMD [SMD (95% CI) = 0.37 (0.02; 0.72)] were observed in vascular stiffness. However, there were no notable differences in the D-D and nFMD parameters.

**Conclusion:**

HIIT notably improved FIB, cfPWV, AIx, and FMD compared to MICT, or UC. Under certain conditions, PLT and AIx@75HR can also benefit from HIIT. It may be particularly advantageous for patients with cardiovascular disease.

**Systematic Review Registration:**

https://www.crd.york.ac.uk/PROSPERO/view/522614, identifier CRD42024522614.

## 1 Introduction

Cardiovascular disease (CVD) is the most prevalent disease globally, responsible for roughly one-third of all global deaths (1, 2). It accounts for 35% of total female deaths and 31% of total male deaths. Additionally, CVD has a serious impact on health losses and expenditure on health system costs (3–5). In 2021, nearly 612 million people worldwide suffered from CVD, with approximately 846 new cases per 100,000 people (6), resulting in approximately 19.4 million deaths and 428.3 million disability-adjusted life years (DALYs) (7). In addition to the health burden, CVD also imposes a significant economic burden on individuals and society. Between 2019 and 2020, the cost of heart disease in the United States was approximately $252.2 billion (8, 9). Globally, the economic burden of CVD is projected to increase from $957 billion in 2015 to $1.04 trillion in 2030 (10).Patients with CVD may exhibit chest pain, dyspnea, dizziness, exercise intolerance, and palpitations (11–13), which largely impair patient's quality of life (14, 15). Moreover, the risk of heart attack, stroke, and other serious cardiovascular events may be increased (16). There is evidence that thrombosis and atherosclerosis, which are largely attributed to hemostasis and vascular stiffness, are involved in the pathogenesis of CVD (17–19). Both the hypercoagulable state of the blood and the progression of atherosclerosis (20, 21) result in an elevated risk of cardiovascular events (16), which in turn causes the occurrence and progression of CVD.

Many studies have emphasized the importance of high-intensity interval training (HIIT) and moderate-intensity continuous training (MICT) for health (22, 23). HIIT is broadly defined as alternating between high-intensity bursts [>80% of maximum oxygen consumption (VO_2max_) or >85% of maximum heart rate (HR_max_)] and low-intensity active recovery (24). MICT is defined as prolonged exercise within the moderate-intensity range (40%–60%) (25, 26). Some studies have shown that HIIT can achieve benefits equivalent to or higher than MICT in a shorter time (27). Compared to traditional MICT, HIIT alternates between short periods of high-intensity exercise and recuperation phases, leading to significant improvements in cardiorespiratory adaptations and metabolic health in a shorter time (28). Recent evidence suggests that HIIT (29, 30) and MICT (22, 31) exercise methods have an effective improvement in both CVD [coronary heart disease (32), heart failure (33–35), stroke (36, 37), and hypertension (38)] and non-CVD [diabetes (39), obesity (40), and healthy adults (41)] patients. These beneficiary groups are diverse, meaning that regardless of age or sex, there is a tendency to benefit from HIIT. Further research has shown that HIIT also has beneficial effects on various cardiovascular-related inflammatory factors (such as IL-6, TNF-α, and IL-10) (42) and biomarkers (such as hs-CRP) (43). Although several studies have been reported on the impacts of HIIT on hemostasis and vascular stiffness in the overall population, there is still controversy over their effectiveness (44, 45). For the effects on hemostasis, existing studies indicate conflicting results. Specifically, HIIT or MICT can improve hemostasis in some studies (40, 46). However, there is no improvement in hemostasis in other studies (47, 48). In addition, the effects on vascular stiffness are also contradictory in existing studies. Specifically, HIIT or MICT improves vascular stiffness in certain studies (49). However, there was no improvement in vascular stiffness in other studies (50, 51). Furthermore, limited studies compare the impacts of HIIT vs. MICT on hemostasis and vascular stiffness. This complicates the identification of the superior exercise modality in clinical settings (32, 52).

This study seeks to synthesize all available evidence using a systematic review and meta-analysis to address the effectiveness of HIIT and MICT in improving hemostasis [platelet count (PLT), fibrinogen (FIB), D-dimer (D-D)] and alleviating vascular stiffness (carotid-femoral pulse wave velocity [cfPWV], augmentation index [AIx], AIx normalized to a heart rate of 75 beats·min−1 [AIx@75HR], flow-mediated dilation [FMD], and brachial flow-mediated dilation normalized [nFMD]) in the overall population, including CVD and non-CVD populations. Additionally, it aims to determine the relative effectiveness of these two exercise approaches. Through this study, we aim to clarify the roles of HIIT and MICT in primary and secondary prevention of CVD, providing new insights for clinical practice.

## 2 Methods

### 2.1 Registration

The study was performed in compliance with the statement of the preferred reporting items for systematic reviews and meta-analyses (PRISMA) (53). The complete checklist can be found in Supplementary Appendix 1. Moreover, it was pre-registered on the international prospective register of systematic reviews (CRD42024522614) (54).

### 2.2 Literature search strategy

After a systematic search, relevant studies were searched from PubMed, Embase, Cochrane Library, and Web of Science. Searches in PubMed, Cochrane Library, and Embase employed a combination of subject and free words. The search strategy in PubMed is presented in the main text (Table 1). Supplementary Appendix 2 details the search strategy for each database. The main terms used to construct the search strategy include “HIIT” and “cardiovascular”. The retrieval strategy is reviewed and adjusted by experienced researchers. In addition, reference lists from chosen studies and reviews were also reviewed to find any pertinent studies that might have been missed in the electronic search. All randomized controlled trials (RCTs) that were written in the English language and published between the inception of the database and June 10, 2025 were included. To ensure the reliability of the study, all included RCTs should be published in peer-reviewed journals.

**Table 1:** Search strategy of pubMed.

Database	Search terms			
	High-intensity interval training	Hemostasis	Vascular stiffness	Randomized controlled trial
PubMed [Title/Abstract]	High-Intensity Interval Training [Mesh Terms] OR high intensity intermittent exercise OR High Intensity Intermittent Exercises OR high intensity intermittent training OR high intensity interval exercise OR high intensity interval training OR High Intensity Interval Trainings OR HIIE OR HIIT OR intermittent high intensity training OR interval high intensity training OR Sprint Interval Training OR Sprint Interval Trainings OR Exercise, High-Intensity Intermittent	Hemostasis [MeSH Terms] OR Blood Platelets [MeSH Terms] OR Fibrinogen [MeSH Terms] OR fibrin fragment D [MeSH Terms] OR blood stasis OR haemostasis OR haemostatic mechanism OR Hemostases OR hemostasis OR hemostatic mechanism OR blood platelet OR blood platelets OR platelet OR Platelets OR thrombocyte OR Thrombocytes OR blood clotting factor i OR Blood Coagulation Factor I OR clottagen OR clotting factor I OR Coagulation Factor I OR factor i OR fibclot OR fibrinogen OR fibryga OR gamma Fibrinogen OR human fibrinogen OR crosslinked fibrin degradation product OR D dimer OR D dimer fibrin OR D dimer fragments OR dimer OR fibrin degradation product d dimer OR fibrin fragment D OR fibrin fragment D dimer OR fibrin fragment D1 dimer OR fibrin fragment DD	Vascular Stiffness [MeSH Terms] OR Carotid-Femoral Pulse Wave Velocity [MeSH Terms] OR aorta stiffness OR aortic stiffening OR “aortic stiffness OR Aortic Stiffnesses OR aortic wall stiffening OR aortic wall stiffness OR arterial stiffening OR arterial stiffness OR Arterial Stiffnesses OR arterial wall stiffening OR arterial wall stiffness OR artery stiffening OR artery stiffness OR artery wall stiffening OR artery wall stiffness OR” vascular stiffness OR Vascular Stiffnesses OR Carotid Femoral Pulse Wave Velocity OR Carotid Femoral Pulse Wave Velocities OR PWV OR pulse wave velocity	Randomized controlled trial OR randomized OR placebo

### 2.3 Eligibility criteria

Studies were included in the analysis according to the following criteria: (P) any subjects aged 18 years and older with or without CVD; (I) studies that used HIIT in the experimental group; (C) studies that used MICT or usual care (UC) in the control group; (O) studies that reported at least one outcome measure about hemostasis (PLT, FIB, D-D) and vascular stiffness (cfPWV, AIx, AIx@75HR, FMD, and nFMD), and (S) RCT. Studies were excluded according to: (i) patients who were unable to receive HIIT for any reason; or (ii) duplicate publications, literature reviews, letters to editors, abstracts presented at conferences, and animal studies. All relevant studies were individually evaluated by two investigators. In case of disagreement, a third investigator reassessed the studies. Only studies that received unanimous agreement from all reviewers were included.

### 2.4 Risk of bias

The risk of bias (ROB) of the included studies was evaluated by two investigators independently using Cochrane's risk-of-bias tool (55). The tool includes seven distinct domains: (a) random sequence generation, (b) allocation concealment, (c) blinding of participants and personnel, (d) blinding of outcome assessment, (e) incomplete outcome data, (f) selective reporting, and (g) other sources of bias.

### 2.5 Data extraction

Data used in the analysis were independently extracted by two investigators. Discrepancies were addressed either through discussion to achieve consensus or by involving a third investigator if necessary. The extracted information included: first author, publication year, country, subject characteristics (experimental and control groups, number, sex, and age), HIIT intervention information (intensity, duration, frequency, and period), and outcome measures. In instances where information was lacking, authors of the included studies were reached out to by email to ask for any missing values. The graphical data extraction software Engauge Digitizer (version 4.1) was used to extract data that were only available in image form.

### 2.6 Statistical analysis

The outcome measures were hemostasis (PLT, FIB, D-D) and vascular stiffness (cfPWV, AIx, AIx@75HR, FMD, nFMD). Meta-analysis was performed only for studies reporting at least one of the above measures. Data synthesis and analysis were carried out focusing on changes from baseline to post-intervention. Since all extracted data were continuous, the analysis employed the standardized mean difference (SMD) and 95% confidence interval (CI). In this study, effect sizes were combined using a fixed-effects model (FEM). If heterogeneity was significant (*I*^2^ > 50%, *P* < 0.05), a random-effects model (REM) was applied instead. Subgroup analyses were performed to assess the impact of various interventions in the control group, the repetition of HIIT intervention, and the presence of CVD on hemostasis and vascular stiffness. The extent of heterogeneity among the included studies was measured by the *I*^2^ test. Sensitivity analysis and meta-regression would be performed if substantial heterogeneity existed. The independent variables for the meta-regression were intensity and duration of HIIT and participant age. Publication bias was evaluated by examining funnel plots. If more than ten studies were included, Egger's test was used to evaluate publication bias. Results were considered statistically significantly different only if the two-sided *P*-value was below 0.05.

All statistical analyses were employed R [version 4.3.3, meta-package (version 4.18-1)].

## 3 Results

### 3.1 Literature selection

**Figure 1:**
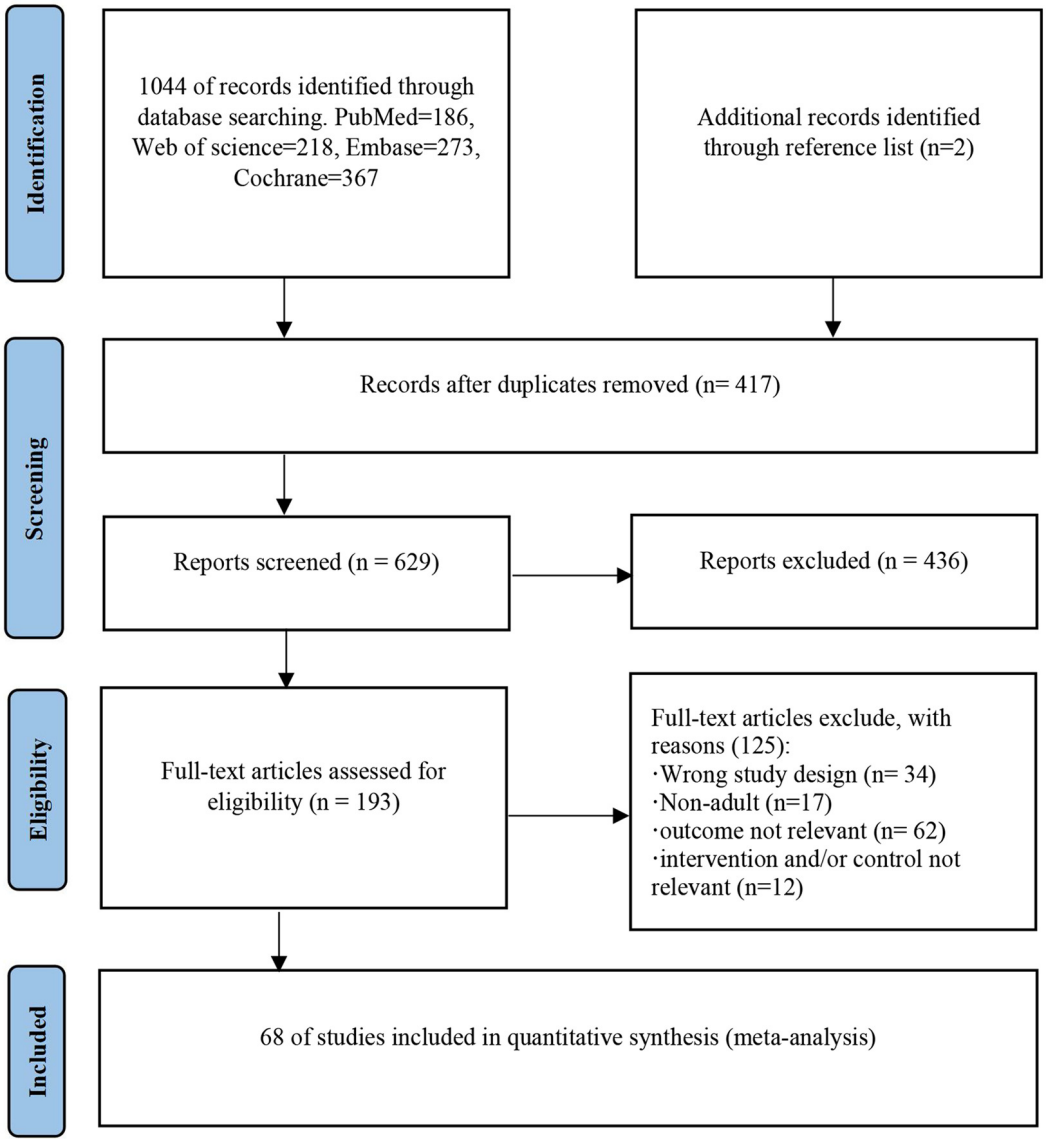
Flow diagram of the preferred reporting items for systematic reviews and meta-analyses depicting the study selection process.

The flow diagram depicting the study selection is presented in Figure 1. After the selection process, 1,046 potentially eligible studies were identified, 1,044 from database searches, and two from reference lists. After removing 417 duplicates, 629 studies remained to be filtered. After screening titles and abstracts, 436 studies were deleted, and 125 were removed post full-text review. Finally, 68 studies were included in the meta-analysis.

### 3.2 ROB analysis for the included studies

The overall ROB was rated as medium to low. The reduced quality of the evidence was primarily attributed to the difficulty in implementing HIIT regarding blinding of participants by implementers. Moreover, the significant intervention nature of HIIT itself made it difficult to adopt a blinded design. Therefore, 100% blinding of participants and personnel, and 2% blinding of outcome assessment were evaluated as high risk. Additionally, it was noted that the loss to follow-up rate reported in four studies exceeded 20%. These studies were rated as high risk unless the study was conducted using an intention-to-treat (ITT) analysis, and it was clearly stated in the paper that loss to follow-up did not affect the comparability between groups, or that the number and reasons for missing data between groups were similar (Supplementary Appendices 3 and 4).

### 3.3 Characteristics of the included studies

Supplementary Appendix 5 outlines the characteristics of the included studies. The list of the included studies is available in Supplementary Appendix 6. These studies were published between 2008 and 2025. 14 studies were performed in North America, one in South America, 28 in Asia, three in Europe, and 10 in Oceania. A total of 1,256 subjects (>18 years) were included in the experimental group and 1,423 in the control group (MICT: 763; UC: 660). In the HIIT group, 80% of subjects were aged 60 or younger, while 20% were older than 60. In the MICT group, 71.1% were aged 60 or younger, and 28.9% were older than 60. In the UC group, 73.8% were aged 60 or younger, and 26.2% were older than 60. About 1,433 (53.5%) subjects were female. Among the included studies, two focused exclusively on females, five on males only, 49 on both sexes, and 12 did not report sex distributions. Additionally, a total of 20 studies involved subjects with CVD.

As outlined in Supplementary Appendix 5, the exercise duration for the HIIT intervention included warm-up and relaxation phases. The mean duration of the exercise interventions was 1–52 weeks, with more than half (51.47%) of the studies reporting up to 8 weeks. Subjects typically engaged in an average of 3.5 sessions per week. A total of 13 studies involved acute exercise. A total of five studies involved two different HIIT intervention protocols, two of which used both short and prolonged HIIT.

High-intensity interval cycling and treadmills were the most commonly used interventions across the studies, with some employing high-intensity interval walking and one focusing on high-intensity interval respiratory muscle training. Of the studies, 51 exclusively used HIIT. In six studies, HIIT was combined with MICT, and resistance training, respectively.

There were significant differences in the intervention measures for the control group UC (Supplementary Appendix 5), which can be primarily categorized into five distinct features: no exercise training (26.3%, e.g., “no exercise training,” “maintaining a sedentary lifestyle”), maintaining daily activities (28.9%, e.g., “continuing routine physical activities,” “maintaining habitual activities”), seated rest (21.1%, e.g., “seated for 30 min,” “seated for 90 min”), standard care or medical management (13.2%, e.g., “standard heart failure management”, “medical care”), and other interventions (10.5%, e.g., green tea, intermittent fasting).

The control group MICT demonstrated moderate consistency in terms of exercise mode, intensity range, and duration (Supplementary Appendix 5). The primary exercise modes were cycling (43.4%) and treadmill/walking (31.2%), with a smaller proportion involving swimming, rowing, or mixed exercises (25.4%). Intensity metrics were primarily based on heart rate (HR_peak_/HR_max_, 60.9%) or oxygen consumption (VO_2peak_/VO2_max_, 34.8%), followed by peak power output (PPO, 10.9%) or the Rating of Perceived Exertion (RPE, 8.7%). Intensity ranges were concentrated between 60 and 75% HR_peak_ (71.4% of the heart rate group) or 50–70% VO_2peak_ (87.5% of the oxygen uptake group). Duration was primarily 30–60 min (89.6%), with only 5 studies lasting less than 30 min or more than 60 min.

### 3.4 Synthesis of the results

#### 3.4.1 PLT analysis

Subgroup analysis based on the repetition of HIIT intervention indicated different results. Nine studies (*n* = 248 subjects) reported that HIIT interventions lasting more than one week with a repetitive exercise pattern led to notable improvements in PLT levels compared to the control group [*I*^2^ = 0%; FEM; SMD (95% CI) = –0.26 (–0.51; −0.01)] (Table 2). In contrast, no significant effect was observed for a single HIIT intervention. A notable difference (*P* = 0.007) in the impact on PLT was observed between repeated HIIT and non-repeated HIIT. In addition, there were no notable differences in PLT levels according to subgroup analyses based on interventions (MICT or UC) in the control group, the presence of CVD, the protocol length of HIIT (≤8 weeks vs. >8 weeks), and exercise modes of HIIT interventions (treadmill, cycling, or others).

**Table 2:** Subgroup analysis results of hemostasis indicators.

Indicators	SMD [95% CI]	Studies	Sample size
PLT			
Total	*p* *=* 0.48		
HIIT vs. Non-HIIT	−0.08 [−0.29; 0.14]	14	348
Different control	*p* = 0.17		
HIIT vs. MICT	−0.24 [−0.52; 0.05]	7	200
HIIT vs. UC	0.13 [−0.19; 0.46]	7	148
Repeat or not	***p* = 0.0069**		
Repeat	**−0.26 [−0.51; −0.01]**	9	248
Not repeat	0.39 [−0.01; 0.78]	5	100
CVD	*p* = 0.82		
With CVD	−0.05 [−0.35; 0.24]	6	182
Without CVD	−0.10 [−0.41; 0.20]	8	166
Protocol length	*p* = 0.08		
≤8 weeks	0.05 [−0.21; 0.30]	12	246
>8 weeks	−0.26 [−0.95; 0.44]	2	102
Mode of HIIT	***p* = 0.03**		
Treadmill	0.33 [−0.07; 0.72]	5	100
Cycling	−0.19 [−0.46; 0.08]	7	220
Other	−0.67 [−1.44; 0.10]	2	28
FIB			
Total	***p* *=* *0.04***		
HIIT vs. Non-HIIT	**−0.60 [−1.18; −0.01]**	15	538
Different control	***p* = 0.03**		
HIIT vs. MICT	0.03 [−0.26; 0.32]	5	185
HIIT vs. UC	**−0.43 [−0.65; −0.21]**	10	353
Repeat or not	***p* = 0.02**		
Repeat	**−0.44 [−0.65; −0.22]**	11	371
Not repeat	0.09 [−0.21; 0.40]	4	167
CVD	*p* = 0.08		
With CVD	**−0.32 [−0.51; −0.12]**	11	442
Without CVD	−0.02 [−0.42; 0.38]	4	96
Protocol length	*p* = 0.14		
≤8 weeks	−1.10 [−2.33; 0.13]	8	254
>8 weeks	−0.17 [−0.40; 0.07]	7	284
Mode of HIIT	*p* = 0.30		
Treadmill	−1.11 [−2.35; 0.13]	8	157
Cycling	−0.13 [−0.34; 0.08]	5	341
Other	−0.07 [−0.70; 0.55]	2	40
D-D			
Total	*p* *=* 0.28		
HIIT vs. Non-HIIT	0.26 [−0.22; 0.75]	5	315
Different control	*p* = 0.34		
HIIT vs. MICT	0.01 [−0.33; 0.35]	2	135
HIIT vs. UC	0.73 [0.43; 1.04]	3	180
Repeat or not	*p* = 0.33		
Repeat	0.67 [0.38; 0.96]	4	198
Not repeat	0.00 [−0.36; 0.37]	1	117
Protocol length	*p* = 0.33		
≤8 weeks	0.00 [−0.36; 0.37]	1	117
>8 weeks	0.35 [−0.25; 0.94]	4	198

CI, confidence interval; Bold, significant difference within a group; Bold, significant difference between groups; PLT, platelet count; FIB, fibrinogen; D-D, D-dimer.

#### 3.4.2 FIB analysis

Subgroup analysis based on interventions in the control group revealed different results. Ten studies (*n* = 353 subjects) reported a difference in FIB levels between before and after HIIT and UC [*I*^2^ = 83%; REM; SMD (95% CI) = −0.98 (−1.87; −0.09)] (Table 2). Additionally, no notable differences in FIB levels were observed between HIIT and MICT. However, the intergroup difference test for MICT and UC reached a significant level (*P* = 0.03).

Subgroup analyses were conducted according to the repetition of HIIT intervention. Eleven studies (*n* = 371 subjects) reported that compared to the control group, a notable difference in FIB was found in the repeated HIIT group when HIIT interventions lasted over one week and employed a repetitive exercise pattern [*I*^2^ = 81%; REM; SMD (95% CI) = −0.93 (−1.71; −0.14)] (Table 2). In addition, no improvement in FIB levels was observed in the single HIIT subgroup. However, subgroup analysis comparing repeated HIIT intervention with a single intervention indicated a notable difference between the two groups (*P* = 0.02).

In the subgroup analysis of the presence of CVD, eleven studies (*n* = 442 subjects) showed that HIIT treatment improved FIB levels in the group of CVD patients (C) [*I*^2^ = 83%; REM; SMD (95% CI) = −0.85 )−1.69; −0.02)] (Table 2).

Additionally, no significant differences were observed between subgroups based on HIIT protocol length (≤8 weeks vs. >8 weeks) and training methods (treadmill, cycling, or other methods).

#### 3.4.3 D-D analysis

In the analysis of D-D, four studies were included, all of which involved CVD patients (*n* = 315 subjects). The results demonstrated that no notable difference was found between the HIIT group and the control group [*I*^2^ = 76%, *P* < 0.01; REM; SMD [95% CI] = 0.26 [−0.22, 0.75], *P* = 0.28] (Supplementary Appendix 7). Additionally, no notable differences were found in D-D levels according to subgroup analyses based on interventions (MICT or UC) in the control group, the repetition of HIIT intervention, and the protocol length of HIIT intervention (≤8 weeks vs. >8 weeks).

#### 3.4.4 cfPWV analysis

Subgroup analysis was conducted based on MICT or UC for the control group. A subgroup analysis containing 33 studies (*n* = 1,114 subjects) indicated a notable difference in cfPWV between HIIT and UC [*I*^2^ = 59.3%; REM; SMD (95% CI) = −0.40 (−0.60; −0.21)] (Table 3). The subgroup analysis based on MICT or UC as the control group showed significant differences between subgroups (*P* = 0.007).

**Table 3:** Subgroup analysis results of vascular stiffness indicators.

Indicators	SMD [95% CI]	Studies	Sample size
cfPWV			
Total	***P* = 0.0019**		
HIIT vs. Non-HIIT	**−0.22 [−0.35; −0.08]**	69	2,209
Different control	***p* = 0.007**		
HIIT vs. MICT	−0.04 [−0.22; 0.14]	36	1,095
HIIT vs. UC	**−0.40 [−0.60; −0.21]**	33	1,114
Repeat or not	*p* = 0.90		
Repeat	**−0.22 [−0.38; −0.06]**	57	1,982
Not repeat	−0.20 [−0.46; 0.06]	12	227
CVD	*p* = 0.68		
With CVD	**−0.24 [−0.42; −0.06]**	19	779
Without CVD	**−0.19 [−0.37; −0.01]**	52	1,430
Protocol length	*p* = 0.28		
≤8 weeks	**−0.13 [−0.27; −0.00]**	35	895
>8 weeks	**−0.28 [−0.52; −0.05]**	34	1,314
Mode of HIIT	*p* = 0.38		
Treadmill	−0.27 [−0.65; −0.10]	22	580
Cycling	**−0.22 [−0.34; −0.10]**	34	1,044
Other	−0.15 [−0.40; 0.10]	13	585
AIx			
Total	***p* *=* 0.01**		
HIIT vs. Non-HIIT	**−0.17 [−0.30; −0.03]**	24	908
Different control	***P* = 0.007**		
HIIT vs. MICT	−0.01 [−0.18; 0.17]	15	517
HIIT vs. UC	**−0.37 [−0.57; −0.17]**	9	391
Repeat or not	*p* = 0.77		
Repeat	**−0.16 [−0.30; −0.02]**	21	834
Not repeat	−0.23 [−0.71; 0.24]	3	74
CVD	*p* = 0.12		
With CVD	0.00 [−0.36; 0.37]	4	283
Without CVD	**−0.24 [−0.40; −0.08]**	20	625
Protocol length	*p* = 0.91		
≤8 weeks	−0.17 [−0.36; 0.01]	13	464
>8 weeks	−0.18 [−0.43; 0.07]	11	444
Mode of HIIT	*p* = 0.36		
Treadmill	−0.32 [−0.65; 0.02]	6	145
Cycling	**−0.21 [−0.41; −0.01]**	13	405
Other	−0.06 [−0.27; 0.15]	5	358
AIx@75HR			
Total	*p* *=* 0.08		
HIIT vs. Non-HIIT	−0.14 [−0.31; 0.02]	19	617
Different control	***p* = 0.01**		
HIIT vs. MICT	0.05 [−0.23; 0.34]	11	310
HIIT vs. UC	**−0.35 [−0.61; −0.10]**	8	307
Repeat or not	*p* = 0.18		
Repeat	−0.20 [−0.47; 0.07]	15	514
Not repeat	0.12 [−0.27; 0.51]	4	103
CVD	*p* = 0.12		
With CVD	**−0.35 [−0.66; −0.05]**	3	166
Without CVD	−0.07 [−0.26; 0.12]	16	451
Protocol length	*p* = 0.06		
≤8 weeks	0.06 [−0.15; 0.27]	11	341
>8 weeks	−0.39 [−0.81; 0.04]	8	276
Mode of HIIT	***p* = 0.04**		
Treadmill	**−0.68 [−1.13;- 0.24]**	4	86
Cycling	−0.10 [−0.31; 0.11]	11	359
Other	−0.00 [−0.30; 0.30]	4	172
FMD			
Total	*p* *=* 0.03		
HIIT vs. Non-HIIT	0.35 [0.03; 0.68]	18	582
Different control	***p* = 0.04**		
HIIT vs. MICT	0.09 [−0.16; 0.34]	12	349
HIIT vs. UC	**0.92 [ 0.15; 1.69]**	6	233
Repeat or not	*p* = 0.53		
Repeat	**0.37 [0.02; 0.72]**	17	542
Not repeat	0.14 [−0.48; 0.76]	1	40
Protocol length	*p* = 0.76		
≤8 weeks	0.43 [−0.34; 1.2]	8	264
>8 weeks	**0.31 [0.08; 0.53]**	10	318
Mode of HIIT	*p* *=* 0.96		
Treadmill	0.30 [−0.06; 0.65]	4	144
Cycling	0.35 [−0.02; 0.90]	11	320
Other	0.40 [−0.26; 1.06]	3	118
nFMD			
Total	*p* *=* 0.16		
HIIT vs. Non-HIIT	0.38 [−0.15; 0.90]	6	187
Different control	*p* = 0.48		
HIIT vs. MICT	0.19 [−0.32; 0.69]	3	63
HIIT vs. UC	0.34 [−0.03; 0.71]	3	124
Protocol length	*p* = 0.36		
≤8 weeks	0.06 [−0.68; 0.80]	2	43
>8 weeks	0.53 [−0.17; 1.24]	4	144

CI, confidence interval; Bold, significant difference within a group; Bold, significant difference between groups; cfPWV, carotid-femoral pulse wave velocity; AIx, augmentation index; AIx@75HR, AIx normalized to a heart rate of 75 beats·min^−1^; FMD, flow-mediated dilation; nFMD, brachial flow-mediated dilation normalized.

Subgroup analysis was performed depending on the repetition of HIIT intervention. A subgroup analysis containing 57 studies (*n* = 1,982 subjects) revealed a notable difference in cfPWV between the repeated HIIT group and the control group [*I*^2^ = 64.5%; REM; SMD (95% CI) = −0.22 (−0.38; −0.06)] (Table 3).

Subgroup analysis depending on the presence of CVD revealed the same results. In subgroup C (*n* = 779 subjects), data from 19 studies showed that HIIT significantly reduced cfPWV levels [*I*^2^ = 32%; REM; SMD (95% CI) = −0.24 (−0.42; −0.06)] (Table 3). The subgroup analysis of non-CVD (NC) patients (*n* = 1,430 subjects) including 52 studies similarly revealed significant improvements in cfPWV after HIIT [*I*^2^ = 63.4%; REM; SMD (95% CI) = −0.19 (−0.37; −0.01)] (Table 3).

Subgroup analysis based on the HIIT protocol duration (≤8 weeks vs. >8 weeks) revealed homogeneous results. 34 studies (*n* = 1,314 participants) reported that HIIT protocol duration exceeded 8 weeks. Compared to the control group, participants showed significant improvements in cfPWV levels in the HIIT group [*I²*=76%; REM; SMD [95% CI] = −0.28 [−0.52; −0.05]; Table 3]. The analysis of the subgroup with ≤8 weeks (*n* = 895 participants) included 35 studies and similarly revealed a significant improvement in cfPWV after HIIT [*I*^2^ = 0%; REM; SMD [95% CI] = −0.13 [−0.27; −0.00]; Table 3].

Subgroup analyses based on HIIT training methods (treadmill, cycling, or other methods) yielded different results. 34 studies (*n* = 1,044 participants) reported that the training method was cycling. Compared with the control group, cfPWV levels showed significant improvement in the HIIT group *[I²*=0%; REM; SMD [95% CI] = −0.22 [−0.34; −0.10]; Table 3]. No significant effects were observed with treadmills or other methods.

#### 3.4.5 Aix analysis

Subgroup analyses were conducted using MICT or UC as the grouping criterion. Specifically, 9 studies in the UC subgroup (*n* = 391 participants) reported AIx levels in response to HIIT and UC, revealing significant differences between the two groups [*I²*=0%; FEM; SMD [95% CI] = −0.37 [−0.57; −0.17]; Table 3]. The results of the subgroup analysis based on the control group being MICT or UC showed significant differences between subgroups (*P* = 0.03).

Subgroup analyses based on HIIT training methods (treadmill, cycling, or other methods) yielded different results. 13 studies (*n* = 405 participants) reported that the HIIT training method was cycling. Compared to the control group, participants showed a significant improvement in AIx levels [*I²*=30.8%; FEM; SMD [95% CI] = −0.21 [−0.41; −0.01]; Table 3]. However, no significant effects were observed for treadmill or other training methods.

Subgroup analysis depending on the presence of CVD showed a notable difference between HIIT in the NC subgroup (*n* = 625 subjects) and the control group [*I*^2^ = 30.6%; REM; SMD (95% CI) = −0.24 (−0.43; −0.05)] (Table 3). Additionally, no notable differences were found in AIx levels according to subgroup analyses based on the repetition of HIIT intervention, and HIIT protocol length (≤8 weeks vs. >8 weeks).

#### 3.4.6 Aix@75hr analysis

Subgroup analysis was conducted depending on MICT or UC for the control group. Specifically, eight studies (*n* = 307 subjects) in the UC subgroup reported a notable difference in AIx@75HR levels between HIIT and UC [*I*^2^ = 34%; FEM; SMD (95% CI) = −0.36 (−0.59; −0.13)] (Table 3). The subgroup analysis based on MICT or UC as the control group demonstrated notable differences between subgroups (*P* *=* 0.01).

Subgroup analysis based on the presence or absence of CVD showed a significant difference between the HIIT group and the control group in the subgroup with CVD (*n* = 166 participants) [*I*^2^ = 37%; FEM; SMD [95% CI] = −0.35 [−0.66; −0.05]; Table 3].

Additionally, no notable differences were found in AIx@75HR levels according to subgroup analyses based on the repetition of HIIT intervention and HIIT protocol length (≤8 weeks vs. >8 weeks).

Subgroup analyses based on HIIT training methods (treadmill, cycling, others) revealed different results. Four studies (*n* = 86 participants) reported that the HIIT training method was treadmill. Compared to the control group, AIx@75HR levels showed significant improvement [*I²*=50%; FEM; SMD [95% CI] = −0.68 [−1.13; −0.24]; Table 3]. However, no significant effects were observed for cycling or other training methods. There were significant differences in the effects of treadmill, cycling, and other training methods on AIx@75HR (*P* = 0.04).

#### 3.4.7 FMD analysis

In the analysis of FMD, 18 studies that exclusively focused on non-CVD patients were included (*n* = 582 subjects). Subgroup analysis was conducted depending on MICT or UC for the control group. Specifically, HIIT was compared with UC in a subgroup analysis containing 6 studies (*n* = 233 subjects). The results indicated a notable difference in FMD between the two groups [*I*^2^ = 82.3%; REM; SMD (95% CI) = 0.92 (0.15; 1.69)] (Table 3). In addition, in the subgroup analysis of the repetition of HIIT intervention containing 17 studies (*n* = 542 subjects), repeated HIIT significantly increased FMD levels [*I*^2^ = 70.4%; REM; SMD (95% CI) = 0.37 (0.02; 0.72)] (Table 3). Additionally, in a subgroup analysis of the HIIT protocol length (≤8 weeks vs. >8 weeks) involving 10 studies (*n* = 318 participants), the >8 weeks subgroup showed a significant improvement in FMD levels [*I²*=2%; REM; SMD [95% CI] = 0.31 [0.08; 0.53]; Table 3]. Finally, no significant differences were observed between subgroups based on the mode of HIIT training (treadmill, cycling, or other methods).

#### 3.4.8 nFMD analysis

In the analysis of nFMD, six studies that exclusively focused on non-CVD patients were included (*n* = 187 subjects). The results demonstrated that there was no notable difference between the HIIT group and the control group [*I*^2^ = 67%, *P* = 0.01; REM; SMD [95% CI] = 0.38 [−0.15, 0.90]. *P* *=* 0.16] (Supplementary Appendix 7). Additionally, no notable differences were found in nFMD levels according to subgroup analysis based on interventions (MICT or UC) in the control group and HIIT protocol length (≤8 weeks vs. >8 weeks) (Supplementary Appendix 8).

### 3.5 Sensitivity analysis

To confirm the robustness of the results, sensitivity analyses were conducted for cfPWV, FMD, FIB, D-D, and nFMD, respectively, demonstrating that the synthesized results were reliable (Supplementary Appendices 9A–9E). In addition, sensitivity analyses were conducted for studies lacking sex information (Supplementary Appendices 9F–9P) and studies that combined other exercise interventions (Supplementary Appendices 9l–S9I). Most indicators were not affected by the lack of sex information or the combination of other exercise interventions.

### 3.6 Meta-regression

In this meta-regression analysis, three potential associations were explored: the relationship of training intensity, training duration, and mean age with PLT, FIB, D-D, cfPWV, AIx, AIx@75HR, FMD, and nFMD levels. The analysis results revealed that the *P*-values for the association between training duration and FIB, AIx, AIx@75HR, FMD, and nFMD levels were 0.15, 0.80, 0.22, 0.61, and 0.32, respectively, and did not reach statistical significance thresholds. It was worth noting that the *P*-value for the correlation between training duration and D-D level was <0.01, with a regression coefficient of −0.08. However, since only five studies were included, the results of this meta-regression should be interpreted with caution (Supplementary Appendix 10A). Additionally, the *P*-value for the correlation between training duration and AIx@75HR level was 0.04, with a regression coefficient of −0.02. This indicated that within the 20–50-min exercise duration, as time increased, the effect of HIIT on reducing AIx@75HR became greater (Supplementary Appendix 10B). Additionally, the *p*-value for the association between training duration and PLT levels was 0.02, with a regression coefficient of −0.07, within the 30–50 min exercise duration range. The effect of HIIT on reducing PLT levels increased as duration increased (Supplementary Appendix 10C). The *P*-value for the association between training duration and cfPWV levels was 0.02, with a regression coefficient of −0.03, within a 20–45-min exercise duration. The effect of HIIT on reducing cfPWV increased as duration increased (Supplementary Appendix 10D). Similarly, the *P*-values for the association between training intensity and PLT, FIB, D-D, AIx, AIx@75HR, FMD, and nFMD levels were 0.08, 0.10, 0.06, 0.44, 0.35, 0.95, and 0.96, respectively, and did not meet conventional statistical significance thresholds. This indicated that the current evidence cannot support a correlation between training intensity and various indicators. Notably, the *P* value for the association between training intensity and cfPWV level was 0.05, and the regression coefficient was 0.0001, within the intensity range of 80–10. The effect of HIIT on reducing cfPWV increased as intensity increased (Supplementary Appendix 10E). Finally, the *P*-values for the association between average age and FIB, D-D, cfPWV, AIx, FMD, and nFMD levels were 0.44, 0.14, 0.20, 0.38, 0.67, 0.09, and 0.44, respectively, and also did not reach statistical significance thresholds.

### 3.7 Publication bias analysis

In the funnel plots of PLT, FIB, cfPWV, AIx, AIx@75HR, and FMD levels, no significant asymmetric distribution was observed. Egger's test was employed to further evaluate publication bias. The results demonstrated that there was no notable publication bias in the analyses of the levels of PLT (*P* = 0.27), FIB (*P* = 0.06), cfPWV (*P* = 0.04), AIx (*P* = 0.29), AIx@75HR (*P* = 0.34) and FMD (*P* = 0.29) (Supplementary Appendices 11A–F). After performing an Egger publication bias test on cfPWV, we found significant publication bias in cfPWV. After adjustment using the trimming method, the results (Supplementary Appendix 11G) were inconsistent with previous findings, indicating that the overall results for cfPWV are unstable. Considering that the control groups in the included studies were MICT or UC, this may be an important factor for this phenomenon. Therefore, we conducted a subgroup analysis. We found that there was no significant difference in the effects of the two intervention methods on cfPWV compared to MICT, and the publication bias was not significant (Egger *p* = 0.18). Compared to the UC subgroup, HIIT significantly improved cfPWV, and the publication bias was not significant (Egger *p* > 0.05).

## 4 Discussion

The results of the meta-analysis suggest that HIIT has an ameliorative effect on cardiovascular risk in the overall population, mainly reflected in the following three aspects: (i) HIIT significantly improves PLT, FIB, cfPWV, AIx, AIx@75HR, and FMD and may be even more effective in patients with CVD; (ii) during HIIT exercise, repeated exercises are preferable, as the benefits from a single session are limited or even ineffective; (iii) Subgroup analysis found that long-term HIIT (>8 weeks) effectively improved atherosclerosis and endothelial function; (iv) based on the results of subgroup analyses, HIIT and MICT do not show any differences in all parameters; and (v) Subgroup analysis also found that training mode (treadmill/cycling) may affect vascular function-related indicators (cfPWV, AIx).

There is evidence that platelet overactivation (56), abnormally elevated FIB (57), cfPWV (58), and Aix (59), and abnormally reduced FMD (60) are associated with the incidence of CVD, such as the development of atherosclerotic plaques (61, 62). HIIT is found to notably improve PLT, FIB, cfPWV, AIx, AIx@75HR, and FMD. Additionally, the effects might be more significant in patients with CVD and may be achieved through multiple mechanisms. Firstly, HIIT reduces platelet activation and aggregation (63, 64) by increasing the levels of nitric oxide (NO) (65, 66) produced by endothelial cells and decreasing the levels of pro-inflammatory cytokines such as C-reactive protein (67, 68) and tumor necrosis factor *α* (69), thereby reducing the risk of thrombosis. Jia et al. demonstrate that strenuous exercise is associated with lower PLT. The reason for this decrease may be that the pattern and intensity of strenuous exercise are different from those of acute exercise (70). Secondly, in patients with CVD, HIIT may reduce plasma levels of fibrinogen by reducing chronic inflammation in the body and promoting the release of anti-inflammatory cytokines such as IL-10 (68, 71, 72). Furthermore, long duration (>8 weeks) and specific modes (treadmill/cycling) of HIIT can increase NO bioavailability and improve endothelial function through the endothelial nitric oxide synthase (eNOS) signaling pathway (73) and by eliciting greater blood flow and shear stress stimulation (74, 75), thereby improving indicators of vascular stiffness such as cfPWV, AIx, AIx@75HR, and FMD. AIx, and its heart rate-adjusted form AIx@75HR, serve as an indirect indicator of vascular stiffness (76). Our results also indicate that AIx@75HR after HIIT notably decreases with the increase in training time, which is similar to a previous study (77). Hence, HIIT is a potent stimulus for the release of NO induced by shear stress (78). Future studies could further explore the repetition of HIIT and its effectiveness in CVD patients.

Our results demonstrate that during HIIT exercise, engaging in repetitive exercises is preferable, as the benefits of a single exercise are limited or even ineffective. Repetitive exercise has been demonstrated to decrease the incidence of platelet overactivation (79). A study by Heber et al. indicates that 12 weeks of HIIT + MICT training can reduce platelet aggregation in patients with coronary heart disease (32). The possible mechanisms are that (i) repetitive release of cytokines, growth factors, or catecholamines may ultimately lead to fewer reactive platelets produced by megakaryocytes (80, 81); and (ii) the reduction of pro-inflammatory state may also have an effect ^21^. Further studies have shown that repetitive training also decreases plasma proteins, such as FIB and albumin, which results in a decrease in plasma viscosity and blood viscosity (82, 83). Notably, regular physical activity is inversely linked to plasma fibrinogen concentrations (83). A decrease in fibrinogen concentration observed following exercise training, especially HIIT, is attributed to the improvement of exercise-induced inflammation and anti-inflammatory properties (84). On the contrary, a single acute exercise may temporarily increase platelet reactivity, thereby promoting thrombosis (85, 86). Sobhani et al. indicate that PLT and FIB are further elevated after two single HIIT training regimens in patients who have undergone coronary artery bypass graft surgery (47), as evidenced by an increase in adenosine diphosphate-induced platelet aggregation. This is possibly due to the higher risk of inducing thrombosis with high-intensity exercise compared to MICT (52, 87). Wang et al. demonstrate the similar results (88). Single exercise-induced platelet aggregation may be linked to several mechanisms, including increased shear stress due to elevated blood flow during exercise (89), augmented catecholamine concentrations (particularly norepinephrine), activation of 2-adrenergic receptors on platelets (90, 91), and heightened oxidative stress (92). Moreover, the increase in platelet function after exercise can be ascribed to the activation of glycoprotein IIb/IIIa receptors and their agonists (93).

This subgroup analysis found that long-term HIIT (>8 weeks) effectively improved arterial stiffness and endothelial function but had no significant effect on coagulation markers (PLT, FIB, D-D). The improvements in cfPWV and FMD may be related to HIIT increasing endothelial NO bioavailability (73) and shear stress-mediated vascular remodeling (74, 75). In contrast, HIIT had no significant effect on coagulation markers (PLT, FIB, D-D) and AIx series markers, possibly because these markers are more significantly influenced by chronic inflammation (71, 72, 94) and genetics (95). Exercise interventions may have a higher threshold for their effects (70) or require combination with other interventions (32).

According to the results of the subgroup analyses in this study, there is no notable difference in the improvement of all parameters between HIIT and MICT, which can be explored from multiple perspectives. Firstly, a reason might cause the failure of detection of difference between HIIT and MICT is the relatively small trial number and sample size, which may disable sufficient narrowing of confidence interval. Secondly, we found that HIIT and MICT showed no significant differences in coagulation and vascular hardening indicators, which may be supported by the following mechanisms (1). The two exercise modes may exert similar effects on vascular stiffening and the coagulation system through common mechanisms [e.g., improving endothelial function (75, 96) and reducing systemic inflammation (97)], such as jointly upregulating NO synthesis, inhibiting vascular smooth muscle proliferation (74), or regulating the metabolic pathways of coagulation factors [e.g., FIB degradation (97)]. (2) Differences in exercise intensity may be offset by the balance of total exercise volume or energy expenditure, especially in long-term interventions. The convergence of the two modes on sympathetic activation or metabolic adaptation may weaken the differences (98, 99). Third, it may be due to the similar impact of the two exercise regimens on cardiovascular risk, such as improving cardiorespiratory function (29), endothelial function (23, 65, 100), left ventricular function (65), and overall myocardial function (101). This improvement has important clinical implications for the health, quality of life, and morbidity and mortality of CVD patients (102). Fourth, there may be differences in HIIT and MICT among different patient groups. However, due to limited existing evidence, it is not yet possible to confirm the existence of such a population group. Lastly, although HIIT is more efficient (103), the risk of injury and compliance issues cannot be ignored. Many articles have overlooked these issues, and we hope that future research will pay more attention to them.

Our subgroup analysis also found that the training mode (treadmill/cycling) influenced vascular function-related indicators (cfPWV, AIx) but had no significant effect on coagulation indicators (PLT, FIB, D-D). This may be related to the shear stress induced by lower limb cyclic exercise activating the eNOS pathway (73) and may also be associated with enhanced leg muscle strength to increase basal leg blood flow (104, 105). Additionally, the absence of changes in coagulation parameters (PLT, FIB, D-D) may be related to exercise intensity not reaching the procoagulant threshold or maintaining appropriate PLT and coagulation (70, 106). Clinically, exercise modes can be personalized based on the type of atherosclerosis.

Although the meta-regression results achieved significance (exercise duration and intensity), their clinical significance is limited due to their low regression coefficients. Further research may be needed to confirm these findings.

Recent studies have focused solely on meta-analyses of the effects of exercise on platelet function (107, 108). However, this study is the first systematic review and meta-analysis to gather comprehensive evidence. It aims to evaluate the impact of HIIT on two key cardiovascular risk factors, hemostasis levels and degree of vascular stiffness, in the overall population. PLT, FIB, cfPWV, AIx, AIx@75HR, and FMD are important biomarkers or functional testing indicators that affect the course of CVD. The study offers a thorough evaluation of how HIIT influences these factors. It further fills the current research gap and provides new evidence for optimizing cardiovascular health management and prevention strategies. There are inevitable limitations to this study. Firstly, due to the nature of HIIT itself, it is difficult to use a blinded design for all included studies. Secondly, HIIT intensity and duration are not consistent across the included studies, which may contribute to a degree of heterogeneity and bias. Factors such as exercise intensity need to be grouped for future assessment. Thirdly, there may be differences in HIIT and MICT among different patient groups. However, due to limited existing evidence, it is uncertain whether such differences can be found among subgroups of people with other diseases. Fourthly, different definitions of control groups across studies may compromise the reliability of results. Finally, publication bias tests were not feasible due to the limited sample size of data in some of the included studies. In addition, future studies can further screen for potential biomarkers closely associated with cardiovascular status, atherosclerosis progression, and aneurysm pathogenesis, such as the matrix metalloproteinase family (MMPs, including MMP-2, MMP-9), which mediate extracellular matrix remodeling.

Repeated training in HIIT might offer greater safety and effectiveness in enhancing cardiovascular health compared to just one exercise session. This finding suggests that medical and fitness professionals should encourage patients to adhere to repeated HIIT exercises within acceptable limits. In addition, future studies should investigate the long-term impacts of HIIT with different quantified exercise intensities and duration on cardiovascular health. The relative merits of HIIT vs. MICT should be further explored. In addition, studies should focus on the repetition of HIIT and its effectiveness in CVD patients.

## 5 Conclusion

In this systematic review and meta-analysis, the results demonstrate that HIIT can significantly improve PLT, FIB, cfPWV, AIx, AIx@75HR, and FMD in the overall population. Moreover, the effect may be even more prominent in CVD patients. Some characteristics, such as repetition, long duration, and specific patterns, are associated with greater benefits. There is no difference between HIIT and MICT in all parameters. It is crucial to interpret the results with caution due to the limited amount of available evidence, and further studies are needed to confirm these findings.

## Data Availability

The original contributions presented in the study are included in the article/Supplementary Material, further inquiries can be directed to the corresponding author.
