# Supplementary material for: Effect of HIIT on hemostasis and vascular stiffness: a systematic review and meta-analysis of randomized controlled trials
Source: Front Cardiovasc Med. 2025 Jul 8;12:1573100. doi: 10.3389/fcvm.2025.1573100 (PMC12279803; doi:10.3389/fcvm.2025.1573100)
Supplement: Supplementary file 1 [file Datasheet1.docx]

| **Supplemental materials** | **Page** |
| --- | --- |
| **Appendix 1.** Completed PRISMA-MA checklist. | 1-2 |
| **Appendix 2.** Search strategy of PubMed, Embase, Cochrane, and Web of Science. | 3-5 |
| **Appendix 3.** Risk of bias assessment. | 6-8 |
| **Appendix 4.** Cochrane risk bias evaluation chart. | 9-10 |
| **Appendix 5.** Characteristics of the included studies. | 11-26 |
| **Appendix 6.** List of included studies. | 27-29 |
| **Appendix 7.** Forest plot for the HIIT effect on PLT, FIB, D-D, cfPWV, AIx, AIx@75HR, FMD, nFMD. | 30-36 |
| **Appendix 8.** Forest plots for subgroup analyses of hemostasis and vascular stiffness indicators. | 37-65 |
| **Appendix 9.** Sensitivity analysis results. | 66-76 |
| **Appendix 10.** Meta-regression analysis results. | 77-78 |
| **Appendix 11.** The funnel plot graphics of PLT, FIB, cfPWV, AIx, AIx@75HR, and FMD in MA. | 79-81 |

**Appendix 1.** PRISMA MA checklist of items to include when reporting a systematic review involving a meta-analysis.

| **Section/topic** | **#** | **Checklist item** | **Reported on page #** |
| --- | --- | --- | --- |
| **TITLE** | | |  |
| Title | 1 | Identify the report as a systematic review, meta-analysis, or both. | 1 |
| **ABSTRACT** | | |  |
| Structured summary | 2 | Provide a structured summary including, as applicable: background; objectives; data sources; study eligibility criteria, participants, and interventions; study appraisal and synthesis methods; results; limitations; conclusions and implications of key findings; systematic review registration number. | 1-2 |
| **INTRODUCTION** | | |  |
| Rationale | 3 | Describe the rationale for the review in the context of what is already known. | 1-2 |
| Objectives | 4 | Provide an explicit statement of questions being addressed with reference to participants, interventions, comparisons, outcomes, and study design (PICOS). | 3-4 |
| **METHODS** | | |  |
| Protocol and registration | 5 | Indicate if a review protocol exists, if and where it can be accessed (e.g., Web address), and, if available, provide registration information including registration number. | 3 |
| Eligibility criteria | 6 | Specify study characteristics (e.g., PICOS, length of follow-up) and report characteristics (e.g., years considered, language, publication status) used as criteria for eligibility, giving rationale. | 3 |
| Information sources | 7 | Describe all information sources (e.g., databases with dates of coverage, contact with study authors to identify additional studies) in the search and date last searched. | 3 |
| Search | 8 | Present full electronic search strategy for at least one database, including any limits used, such that it could be repeated. | Table 1, Appendix 2 |
| Study selection | 9 | State the process for selecting studies (i.e., screening, eligibility, included in systematic review, and, if applicable, included in the meta-analysis). | 3 |
| Data collection process | 10 | Describe method of data extraction from reports (e.g., piloted forms, independently, in duplicate) and any processes for obtaining and confirming data from investigators. | 3 |
| Data items | 11 | List and define all variables for which data were sought (e.g., PICOS, funding sources) and any assumptions and simplifications made. | 3 |
| Risk of bias in individual studies | 12 | Describe methods used for assessing risk of bias of individual studies (including specification of whether this was done at the study or outcome level), and how this information is to be used in any data synthesis. | 3 |
| Summary measures | 13 | State the principal summary measures (e.g., risk ratio, difference in means). | 3 |
| Synthesis of results | 14 | Describe the methods of handling data and combining results of studies, if done, including measures of consistency (e.g., I^2^) for each meta-analysis. | 4 |

| **Section/topic** | **#** | **Checklist item** | **Reported on page #** |
| --- | --- | --- | --- |
| Risk of bias across studies | 15 | Specify any assessment of risk of bias that may affect the cumulative evidence (e.g., publication bias, selective reporting within studies). | 3 |
| Additional analyses | 16 | Describe methods of additional analyses (e.g., sensitivity or subgroup analyses, meta-regression), if done, indicating which were pre-specified. | 4 |
| **RESULTS** | | |  |
| Study selection | 17 | Give numbers of studies screened, assessed for eligibility, and included in the review, with reasons for exclusions at each stage, ideally with a flow diagram. | 4 |
| Study characteristics | 18 | For each study, present characteristics for which data were extracted (e.g., study size, PICOS, follow-up period) and provide the citations. | Appendix 3 |
| Risk of bias within studies | 19 | Present data on risk of bias of each study and, if available, any outcome level assessment (see item 12). | Appendix 5 |
| Results of individual studies | 20 | For all outcomes considered (benefits or harms), present, for each study: (a) simple summary data for each intervention group (b) effect estimates and confidence intervals, ideally with a forest plot. | 5-8 |
| Synthesis of results | 21 | Present results of each meta-analysis done, including confidence intervals and measures of consistency. | 5-8, Table 2, Table 3 |
| Risk of bias across studies | 22 | Present results of any assessment of risk of bias across studies (see Item 15). | 4 |
| Additional analysis | 23 | Give results of additional analyses, if done (e.g., sensitivity or subgroup analyses, meta-regression [see Item 16]). | 5-9, Table 2, Table 3 |
| **DISCUSSION** | | |  |
| Summary of evidence | 24 | Summarize the main findings including the strength of evidence for each main outcome; consider their relevance to key groups (e.g., healthcare providers, users, and policy makers). | 9-11 |
| Limitations | 25 | Discuss limitations at study and outcome level (e.g., risk of bias), and at review-level (e.g., incomplete retrieval of identified research, reporting bias). | 11 |
| Conclusions | 26 | Provide a general interpretation of the results in the context of other evidence, and implications for future research. | 11-12 |
| **FUNDING** | | |  |
| Funding | 27 | Describe sources of funding for the systematic review and other support (e.g., supply of data); role of funders for the systematic review. | 12 |

*From:*  Page MJ, McKenzie JE, Bossuyt PM, Boutron I, Hoffmann TC, Mulrow CD, et al. The PRISMA 2020 statement: an updated guideline for reporting systematic reviews. BMJ 2021;372:n71. doi: 10.1136/bmj.n71

**Appendix 2.** Search strategy of PubMed, Embase, Cochrane, and Web of Science.

| **Database** | **Search Terms** | | | |
| --- | --- | --- | --- | --- |
|  | High-Intensity Interval Training | **Hemostasis** | **Vascular Stiffness** | **Randomized controlled**  **trial** |
| PubMed [Title/Abstract] | High-Intensity Interval Training [Mesh Terms] OR high intensity intermittent exercise OR High Intensity Intermittent Exercises OR high intensity intermittent training OR high intensity interval exercise OR high intensity interval training OR High Intensity Interval Trainings OR HIIE OR HIIT OR intermittent high intensity training OR interval high intensity training OR Sprint Interval Training OR Sprint Interval Trainings OR Exercise, High-Intensity Intermittent | Hemostasis [MeSH Terms] OR Blood Platelets [MeSH Terms] OR Fibrinogen [MeSH Terms] OR fibrin fragment D [MeSH Terms] OR blood stasis OR haemostasis OR haemostatic mechanism OR Hemostases OR hemostasis OR hemostatic mechanism OR blood platelet OR blood platelets OR platelet OR Platelets OR thrombocyte OR Thrombocytes OR blood clotting factor i OR Blood Coagulation Factor I OR clottagen OR clotting factor I OR Coagulation Factor I OR factor i OR fibclot OR fibrinogen OR fibryga OR gamma Fibrinogen OR human fibrinogen OR crosslinked fibrin degradation product OR D dimer OR D dimer fibrin OR D dimer fragments OR dimer OR fibrin degradation product d dimer OR fibrin fragment D OR fibrin fragment D dimer OR fibrin fragment D1 dimer OR fibrin fragment DD | Vascular Stiffness [MeSH Terms] OR Carotid-Femoral Pulse Wave Velocity [MeSH Terms] OR aorta stiffness OR aortic stiffening OR "aortic stiffness OR Aortic Stiffnesses OR aortic wall stiffening OR aortic wall stiffness OR arterial stiffening OR arterial stiffness OR Arterial Stiffnesses OR arterial wall stiffening OR arterial wall stiffness OR artery stiffening OR artery stiffness OR artery wall stiffening OR artery wall stiffness OR "vascular stiffness OR Vascular Stiffnesses OR Carotid Femoral Pulse Wave Velocity OR Carotid Femoral Pulse Wave Velocities OR PWV OR pulse wave velocity | Randomized controlled trial OR randomized OR placebo |
| Embase [Title/Abstract] | high intensity interval training [Emtree term] OR high intensity intermittent exercise OR High Intensity Intermittent Exercises OR high intensity intermittent training OR high intensity interval exercise OR High Intensity Interval Trainings OR HIIE OR HIIT OR intermittent high intensity training OR interval high intensity training OR Sprint Interval Training OR Sprint Interval Trainings | blood stasis [Emtree term] OR haemostasis OR haemostatic mechanism OR Hemostases OR hemostasis OR hemostatic mechanism OR blood platelet OR blood platelets OR platelet OR Platelets OR thrombocyte OR Thrombocytes OR blood clotting factor i OR Blood Coagulation Factor I OR clottagen OR clotting factor I OR Coagulation Factor I OR factor i OR fibclot OR fibrinogen OR fibryga OR gamma Fibrinogen OR human fibrinogen OR crosslinked fibrin degradation product OR D dimer OR D dimer fibrin OR D dimer fragments OR dimer OR fibrin degradation product d dimer OR fibrin fragment D OR fibrin fragment D dimer OR fibrin fragment D1 dimer OR fibrin fragment DD | arterial stiffness [Emtree term] OR aortic stiffening OR aortic stiffness OR Aortic Stiffnesses OR aortic wall stiffening OR aortic wall stiffness OR arterial stiffening OR arterial stiffness OR Arterial Stiffnesses OR arterial wall stiffening OR arterial wall stiffness OR artery stiffening OR artery stiffness OR artery wall stiffening OR artery wall stiffness OR vascular stiffness OR Vascular Stiffnesses OR Carotid Femoral Pulse Wave Velocity OR Carotid Femoral Pulse Wave Velocities OR PWV OR pulse wave velocity | randomized controlled trial OR randomized OR placebo |
| Cochrane [Title/Abstract/ keywords] | High-Intensity Interval Training [Mesh Terms] OR high intensity intermittent exercise OR High Intensity Intermittent Exercises OR high intensity intermittent training OR high intensity interval exercise OR high intensity interval training OR High Intensity Interval Trainings OR HIIE OR HIIT OR intermittent high intensity training OR interval high intensity training OR Sprint Interval Training OR Sprint Interval Trainings OR Exercise, High-Intensity Intermittent | Hemostasis [MeSH Terms] OR Blood Platelets [MeSH Terms] OR Fibrinogen [MeSH Terms] OR fibrin fragment D [MeSH Terms] OR blood stasis OR haemostasis OR haemostatic mechanism OR Hemostases OR hemostasis OR hemostatic mechanism OR blood platelet OR blood platelets OR platelet OR Platelets OR thrombocyte OR Thrombocytes OR blood clotting factor i OR Blood Coagulation Factor I OR clottagen OR clotting factor I OR Coagulation Factor I OR factor i OR fibclot OR fibrinogen OR fibryga OR gamma Fibrinogen OR human fibrinogen OR crosslinked fibrin degradation product OR D dimer OR D dimer fibrin OR D dimer fragments OR dimer OR fibrin degradation product d dimer OR fibrin fragment D OR fibrin fragment D dimer OR fibrin fragment D1 dimer OR fibrin fragment DD | Vascular Stiffness [MeSH Terms] OR Carotid-Femoral Pulse Wave Velocity [MeSH Terms] OR aorta stiffness OR aortic stiffening OR "aortic stiffness OR Aortic Stiffnesses OR aortic wall stiffening OR aortic wall stiffness OR arterial stiffening OR arterial stiffness OR Arterial Stiffnesses OR arterial wall stiffening OR arterial wall stiffness OR artery stiffening OR artery stiffness OR artery wall stiffening OR artery wall stiffness OR "vascular stiffness OR Vascular Stiffnesses OR Carotid Femoral Pulse Wave Velocity OR Carotid Femoral Pulse Wave Velocities OR PWV OR pulse wave velocity | — |
| Web of Science | high intensity intermittent exercise OR High Intensity Intermittent Exercises OR high intensity intermittent training OR high intensity interval exercise OR high intensity interval training OR High Intensity Interval Trainings OR HIIE OR HIIT OR intermittent high intensity training OR interval high intensity training OR Sprint Interval Training OR Sprint Interval Trainings | blood stasis OR haemostasis OR haemostatic mechanism OR Hemostases OR hemostasis OR hemostatic mechanism OR blood platelet OR blood platelets OR platelet OR Platelets OR thrombocyte OR Thrombocytes OR blood clotting factor i OR Blood Coagulation Factor I OR clottagen OR clotting factor I OR Coagulation Factor I OR factor i OR fibclot OR fibrinogen OR fibryga OR gamma Fibrinogen OR human fibrinogen OR crosslinked fibrin degradation product OR D dimer OR D dimer fibrin OR D dimer fragments OR dimer OR fibrin degradation product d dimer OR fibrin fragment D OR fibrin fragment D dimer OR fibrin fragment D1 dimer OR fibrin fragment DD | aorta stiffness OR aortic stiffening OR aortic stiffness OR Aortic Stiffnesses OR aortic wall stiffening OR aortic wall stiffness OR arterial stiffening OR arterial stiffness OR Arterial Stiffnesses OR arterial wall stiffening OR arterial wall stiffness OR artery stiffening OR artery stiffness OR artery wall stiffening OR artery wall stiffness OR vascular stiffness OR Vascular Stiffnesses OR Carotid Femoral Pulse Wave Velocity OR Carotid Femoral Pulse Wave Velocities OR PWV OR pulse wave velocity | randomized controlled trial OR randomized OR placebo |

**Appendix 3.** Risk of bias assessment.

| **Study** | **Allocation generation** | **Concealment of allocation** | **blinding of participates and personnel** | **Blinding of outcome**  **assessment** | **Dropout rate %** | **Selective reporting** | **Other bias** |
| --- | --- | --- | --- | --- | --- | --- | --- |
| Taylor 2022 | Low | Unclear | High | Low | 4% | Low | Low |
| Ahmadizad 2017 | Unclear | Unclear | High | Unclear | Not reported | Low | Low |
| Avazpour 2023 | Unclear | Unclear | High | Unclear | Not reported | Low | Low |
| Chrysohoou 2015 | Low | Unclear | High | Unclear | 0% | Low | Low |
| Ciolac 2010 | Unclear | Unclear | High | Low | Low | Unclear | Low |
| Dall 2015 | Low | Low | High | Unclear | 15% | Low | Low |
| Gevaert 2023 | Low | Unclear | High | Unclear | 2% | Low | Low |
| Guimara˜es 2010 | Low | Low | High | Low | Low | Low | Low |
| Hearon 2022 | Low | Unclear | High | Low | Low | Low | Low |
| Heber 2020 | Low | Low | High | Low | 18% | Low | Low |
| Košuta 2024 | Low | Low | High | Low | 7% | Unclear | Low |
| Kristiansen 2022 | Low | Low | High | Unclear | 16% | Low | Low |
| Marçal 2022 | Low | Low | High | Unclear | 18% | Low | Low |
| Mora-Rodriguez 2018 | Unclear | Unclear | High | Unclear | 8% | Unclear | Low |
| Novakovi´c 2018 | Low | Low | High | High | 1% | Unclear | Low |
| Sobhani 2018 | Low | Low | High | Unclear | 13% | Unclear | Low |
| Soltani 2020 | Unclear | Unclear | High | Unclear | Not reported | Low | Low |
| Tanriverdi 2023 | Low | Low | High | Low | 0% | Low | Low |
| Valizadeh 2017 | Unclear | Unclear | High | Unclear | Not reported | Unclear | Low |
| Aghaei Bahmanbeglou 2019 | Unclear | Unclear | High | Unclear | 1% | Unclear | Low |
| Sosner 2019 | Low | Low | High | Unclear | 0% | Unclear | Low |
| Alis 2015 | Unclear | Unclear | High | Unclear | Not reported | Unclear | Low |
| Ghasemi 2019 | Low | Unclear | High | Unclear | 0% | Unclear | Low |
| Sheykhlouvand 2018 | Unclear | Unclear | High | Low | Not reported | Unclear | Low |
| Whittaker 2013 | Low | Unclear | High | Low | Not reported | Unclear | Low |
| Wu 2017 | Unclear | Unclear | High | Unclear | Not reported | Unclear | Low |
| Adams 2017 | Low | Low | High | Low | 3% | Unclear | Low |
| Battillo 2023 | Low | Unclear | High | Unclear | Not reported | Unclear | Low |
| Bellia 2017 | Unclear | Unclear | High | Unclear | Low | Unclear | Low |
| Beltrami 2020 | Unclear | Unclear | High | Unclear | Not reported | Unclear | Low |
| Clark 2020 | Low | Low | High | Unclear | 24% | Low | Low |
| Cocks 2013 | Unclear | Unclear | High | Low | Not reported | Unclear | Low |
| Cocks 2016 | Low | Unclear | High | Unclear | Not reported | Unclear | Low |
| Cooke 2022 | Low | Unclear | High | Unclear | ITT | Low | Low |
| Davoodi 2022 | Low | Unclear | High | Low | Low | Low | Low |
| Deiseroth 2019 | Low | Unclear | High | Low | 14% | Low | Low |
| Eichner 2018 | Low | Unclear | High | Unclear | Not reported | High | Low |
| Fernandes 2020 | Low | Unclear | High | Low | 22% | Unclear | Low |
| Heydari 2013 | Low | Low | High | Low | 17.3% | Low | Low |
| Hanssen 2017 | Low | Unclear | High | Unclear | Low | Low | Low |
| Hanssen 2018 | Low | Unclear | High | Unclear | Low | Unclear | Low |
| Ho 2020 | Unclear | Unclear | High | Unclear | 0% | Unclear | Low |
| Kim 2017 | Low | Low | High | Low | 18% | Unclear | Low |
| Magalhães 2019 | Low | Low | High | Unclear | ITT | Unclear | Low |
| Magalhães 2021 | Low | Low | High | Unclear | Not reported | Unclear | Low |
| Okamoto 2019 | Unclear | Unclear | High | Low | Low | Unclear | Low |
| Petrick 2021 | Low | Low | High | Low | Not reported | Low | Low |
| Rakobowchuk 2008 | Low | Unclear | High | Unclear | Not reported | Unclear | Low |
| Ramírez-Vélez 2019 | Low | Low | High | Low | 5% | Low | Low |
| Ramírez-Vélez 2020 | Low | Low | High | Unclear | 19% | Unclear | Low |
| Scott 2019a | Unclear | Unclear | High | Unclear | 29% | Unclear | Low |
| Scott 2019b | Unclear | Unclear | High | Unclear | Not reported | Low | Low |
| Soltani 2021 | Low | Unclear | High | Unclear | 12% | Unclear | Low |
| Tang 2022 | Unclear | Unclear | High | Unclear | 16% | Low | Low |
| Tangchaisuriya 2022 | Low | Unclear | High | Unclear | 0% | Unclear | Low |
| Way 2020 | Low | Low | High | Low | 9% | Low | Low |
| Hanssen 2015 | Unclear | Unclear | High | Unclear | 33% | Unclear | Low |
| Marcal 2021 | Low | Unclear | High | Unclear | Not reported | Unclear | Low |
| Perissiou 2018 | Unclear | Unclear | High | Unclear | Not reported | Unclear | Low |
| Sapp 2020 | Unclear | Unclear | High | Unclear | 0% | Unclear | Low |
| Siasos 2016a | Unclear | Unclear | High | Unclear | Not reported | Unclear | Low |
| Siasos 2016b | Unclear | Unclear | High | Unclear | Not reported | Unclear | Low |
| Viana 2018 | Low | Low | High | Low | Not reported | Unclear | Low |
| Melo 2024 | Low | Low | High | Unclear | ITT | Low | Low |
| Cox 2024 | Low | Low | High | Low | Not reported | Low | Low |
| Huang 2025 | Unclear | Unclear | High | Unclear | 10% | Low | Low |
| Moncion 2024 | Low | Low | High | Low | ITT | Low | Low |
| Zarekar 2024 | Unclear | Unclear | High | Unclear | Not reported | Low | Low |

**Appendix 4.** Cochrane risk bias evaluation chart.


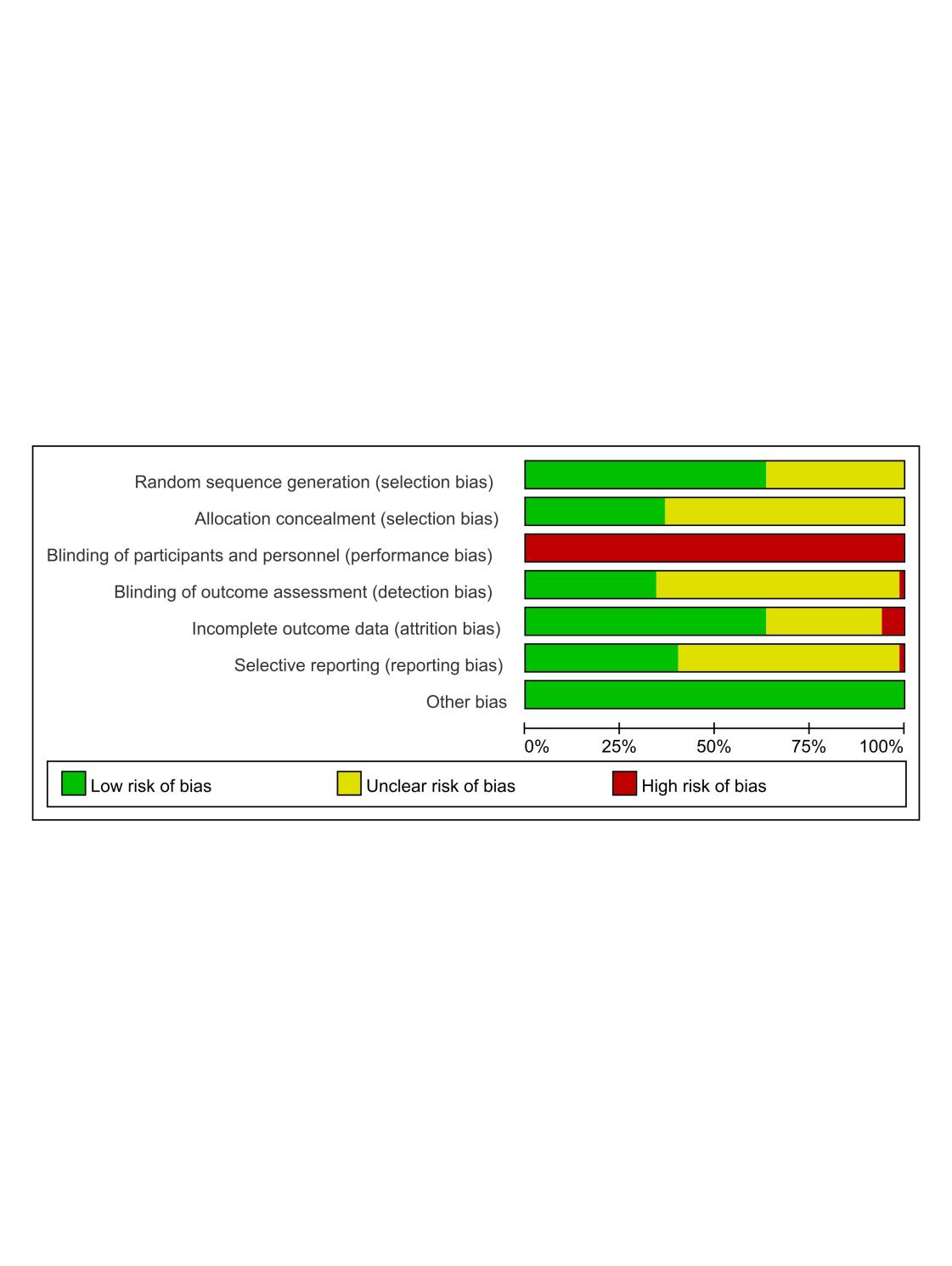


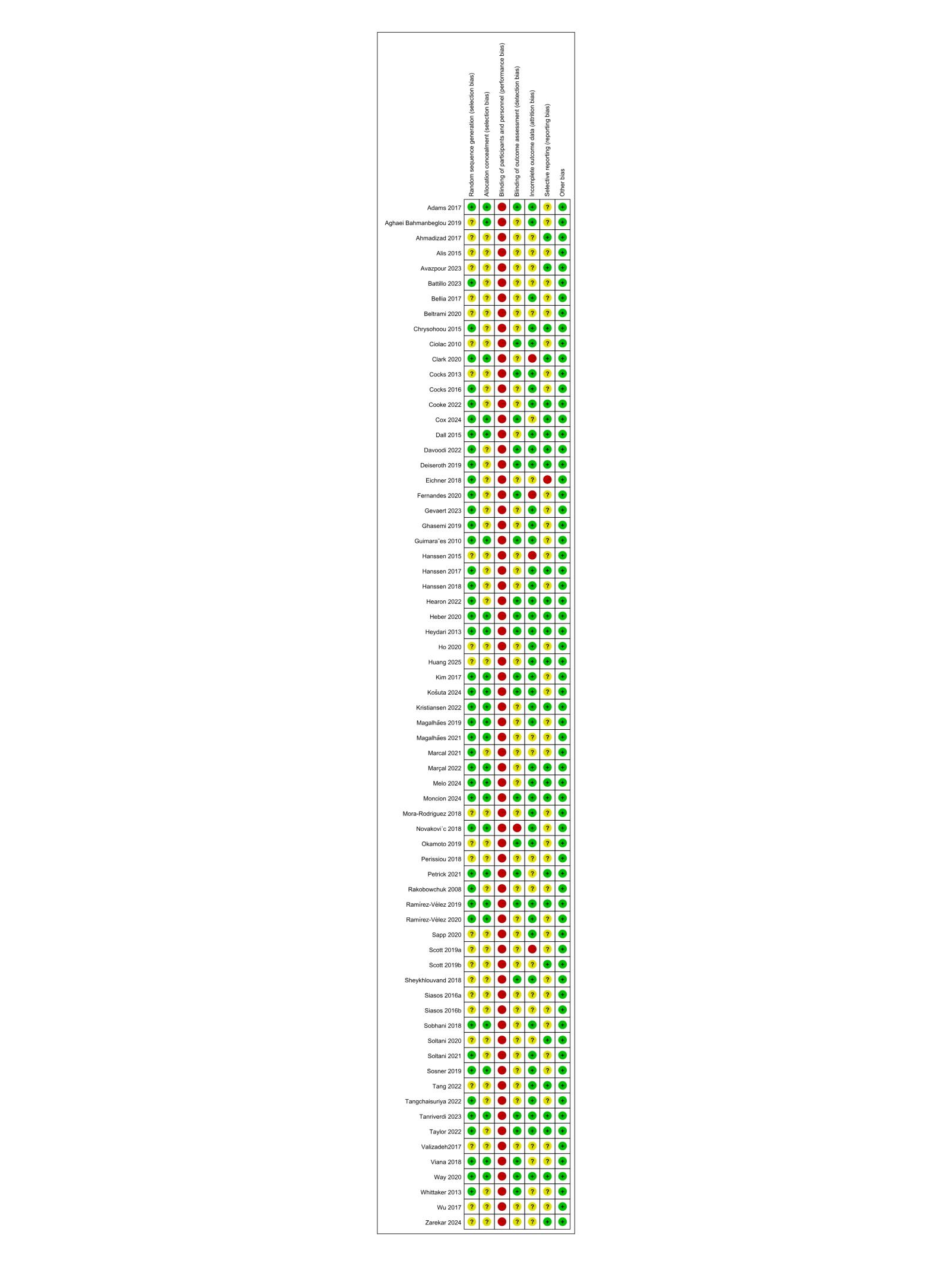


**Appendix 5.** Characteristics of the included studies.

| Author and  published  year | Country | Patients  group | Control |  | HIIT |  | Intensity | Intervention duration (minutes per session, times per week, total weeks) | Outcomes |
| --- | --- | --- | --- | --- | --- | --- | --- | --- | --- |
|  |  |  | Sample size (M/F) | Age | Sample size (M/F) | Age |  |  |  |
| Ciolac 2010^1^ | Brazil | healthy FH+ women | MICT: 16(5/3), UC: 12(4/4) | MICT: 26.6±4.9, UC: 25.3±3.7 | 16(2/9) | 24.4±3.8 | Mode: walking/ running on a treadmill.  Warm-up: 2 min walking at the anaerobic threshold (50–60% of VO_2max_). HIIT: 1 min CRP 80-90% VO_2max_, alternate walking/running. Cool down: no report | 40 min/day, 3 times/week, 16 weeks | ① |
| Guimara˜es 2010^2^ | Brazil | HBP | MICT: 16(9/7), UC: 11(9/2) | MICT: 50±8, UC: 47±6 | 16(12/4) | 45±9 | Mode: treadmill.  Warm-up: 10 min stretching exercises to reach 60% of reserve heart rate. HIIT: alternate between 50% (2 min) and 80% (1 min) of reserve heart rate while using the treadmill. Cool down: 10 min exercises. | 40 min/day, 3 times/week, 16 weeks | ① |
| Chrysohoou 2015^3^ | Greece | CHF | UC: 39(28/11) | UC: 56 ± 11 | 33(29/4) | 63 ± 9 | Mode: cycling.  Warm-up: no report. HIIT: cycling(80%WR_peak_-100%WR_peak_) and rest (1:1). Cool down: no report  RE: fitness equipment at 30%-90% 1RM | 45 min/day, 3 times/week, 12 weeks | ① |
| Dall 2015^4^ | Denmark | HTx | 16(12/4) | 51.9 (33–70) | 16(12/4) | 51.9 (33–70) | Mode: ergometer bikes.  Warm-up: 10 min 50% VO_2peak_ cycling. HIIT: alternating intervals of 4, 2 and 1 minute’s duration at >80% of VO_2peak_, each separated by a 2 min active rest period (appr. 60% of VO_2peak_), during cycling. Cool down: 10 min 60% VO_2peak_ cycling | 30 min/day, 3 times/week, 12 weeks | ② |
| Ahmadizad 2017^5^ | Iran | CABG, PCI | MICT: 10(-), UC: 10(-) | MICT: 62.0±4.2, UC: 59.0±4.5 | 10(-) | 60.6±2.3 | Mode: motorized treadmill.  Warm-up: 5 min cycling. HIIT: treadmill 90% VO_2peak_ and 30% VO_2peak_ active recovery. Cool down: 10 min 60% VO_2peak_ cycling. | 30 min, acute exercise | ③ |
| Valizadeh 2017^6^ | Iran | HBP | UC: 10(-) | UC: 62.50±2.99 | 10(-) | 58±4.94 | Mode: matrix model treadmill.  Warm-up: 5-10 min balance drills. HIIT: 80% - 95% MHRR and 50% - 70% MHRR active recovery. Cool down: 5-10 min | 30-45 min/day, 3 times/week, 12 weeks | ③④⑤ |
| Mora-Rodriguez 2018^7^ | Spain | metabolic syndrome | UC: 23(-) | UC: 53.5±8.9 | 23(-) | 53.5±8.9 | Mode: cycle ergometer.  Warm-up: 10 min. HIIT: pedaling at 90% of HR_MAX_ and 3 min active recovery periods at 70% HR_MAX_. Cool down: no report | 45 min/day, 3 times/week, 6 months | ①③④⑥ |
| Novakovi´c 2018^8^ | Slovenia | ToF | MICT: 9(5/4), UC: 9(5/4) | MICT: 40.1±10.4, UC: 38.4±8.9 | 9(7/2) | 36.2±6.8 | Mode: cycling.  Warm-up: 5 min 50% HR_peak_ cycling and/or speed walking. HIIT: 8 ∗(1 min 80% HR_peak_ and 3 minute 60% HR_peak_). Cool-down: 5 min 50% HR_peak_ cycling and/or speed walking. | 42 min/day, 3 times/week, 14 weeks | ①④⑤⑦ |
| Sobhani 2018^9^ | Iran | CABG | MICT: 10(-) | MICT: 53.9±3.45 | HIIT-1: 10(-), HIIT-2: 10(-) | HIIT-1: 53.7±3.4, HIIT-2: 54.1±4.01 | Mode: treadmill.  Warm-up: 5-min 40% HR_MAX_ walking or running and stretching movements. HIIT-1: 10∗(2 min 95% HR_MAX_ running and 2 min 35% HR_MAX_ active recovery). HIIT-2: 7∗(4 min 85% of HR_MAX_ running and 2 min 45% HR_MAX_ active recovery). Cool down: no report | HIIT-1: 40 min, acute exercise HIIT-2: 42 min,, acute exercise | ③④ |
| Heber 2020^10^ | Austria | CHD | MICT: 42(-) | MICT: 61.7±9.8 | 40(-) | 60±9.4 | Mode: bicycle ergometers.  HIIT+MICT:  Warm-up: 5 min at 40% of P_max_. HIIT: 15∗(1 min 100% of P_max_ bicycle ergometers and 1 min 20% of P_max_ active recovery). Cool-down: 10 min at 30% of P_max_.  MICT: 45 min at 60% of of P_max_ bicycle | 45 min/day, 2 times/week, 12 weeks | ③ |
| Soltani 2020^11^ | Iran | HBP | UC: 10(-) | UC: 47.0 ± 3.0 | SDHIIT: 10(-), LDHIIT: 10(-) | SDHIIT: 48±5, LDHIIT: 48.8±5 | Mode: motorized treadmill.  Warm-up: 5 min stretching exercises, walking and jogging. SDHIIT: 27∗(30s 80%-100% VO_2peak_ exercise and 30s 10%-20% VO_2peak_ active recovery). LDHIIT: 4∗(4 min 75%-90% VO_2peak_ exercise and 4 min 15%-30% VO_2peak_ active recovery). Cool down: 5 min. | SDHIIT: 37 min/day, 3 times/week, 8 weeks LDHIIT: 37 min/day, 3 times/week, 8 weeks | ④ |
| Taylor 2022^12^ | Australia | CAD | MICT: 26(24/2) | MICT: 63 ± 8 | 28(26/2) | 64 ± 8 | Mode: outdoor walking or gym (e.g., bike, treadmill, elliptical).  Warm-up: no report. HIIT: 4∗(4 min (RPE) 15–18 and 3 min active recovery). Cool down: no report. | 31 min/day, 3 times/week, 4 weeks | ①②⑥⑦ |
| Hearon 2022^13^ | USA | HF(stage A) | UC-Placebo: 13(7/6) UC-n-3 FA: 14(4/10) | UC-Placebo: 49±6 UC-n-3 FA: 47±9 | HIIT-Placebo: 16(8/8) HIIT-n-3 FA: 13(5/8) | HIIT-Placebo: 50±6 HIIT-n-3 FA: 50±6 | Mode: cycle ergometer.  Warm-up: no report. HIIT: (5-8)∗((0.5-2) min >95% HR_peak_ cycling and 2 min < base pace active recovery). Cool down: no report. | 30 min/day, 2 times/week, 1 year | ①⑥ |
| Kristiansen 2022^14^ | UK | CHD | UC: 78(64/14) | UC: 66.4±9.3 | 64(54/10) | 67.0±9.5 | Mode: electronically braked cycle ergometer.  Warm-up: 6 min rowing of 30-70Wmax. HIIT: 8∗(1.5 min 100Wmax and 1.5 min active recovery). Cool down: 4 min 50 W_max_. | 30 min/day, 3 times/week, 12 weeks | ④⑤ |
| Marçal 2022^15^ | Brazil | HBP | 20(9/11) | 67 ± 7 | 20(9/11) | 67 ± 7 | Mode: swimming pool.  Warm-up: 4 min RPE 9 walking. HIIT: 7∗(1 min RPE 15-17 jogging/running and 2 min RPE 9-11 active recovery). Cool down: no report. | 25 min, acute exercise | ① |
| Avazpour 2023^16^ | Iran | CAD | UC: 11(-) | UC: 57.8±3.4 | HIIT: 12(-), HIIT+MICT: 13(-) | HIIT: 54.6±3.7, HIIT+AE: 56.5±2.01 | Mode: running on a treadmill.  HIIT+MICT:  Warm-up: 5 min stretching and light running. First 4 weeks of AE: 20-25 min 50-70% VO_2peak_ running. Next 4 weeks: MICT: 25-30 min 50-70% VO_2peak_ running, and HIIT: 20-25 min 70-100% VO_2peak_ running and 15 s-1 min active recovery. Cool down: 5 min.  Warm up: 5 min stretching and light running. HIIT: 15–25 min 70–100% VO_2peak_ and 15 s-1 min active recovery. Cool down: 5 min. | HIIT+MICT: First 4 weeks: MICT: 20-25 min/day, 2-3 times/week; Next 4 weeks: MICT: 25-30 min/day, 2 times/week, and HIIT: 20-25 min/day, 1 times/week. HIIT: 15–25 min/day, 3 times/week, 8 weeks | ④ |
| Gevaert 2023^17^ | Belgium | HFpEF | MICT: 53(-), UC: 53(-) | 70 | 53(-) | 70 | Mode: cardiac rehabilitation centers (the bicycle and the treadmill).  Warm-up: 10 min 35%-50% heart rate reserve. HIIT: 4∗(4 min 80%-90% heart rate reserve and 3 min 35%-50% heart rate reserve). Cool down: no report. | 38 min/day, 3 times/week, 3 months | ①② |
| Tanriverdi 2023^18^ | Turkey | HFpEF | UC: 17(13/4) | UC: 62.5±8.1 | 17(13/4) | 63.7±7.6 | Mode: Threshold pressure loading device.  Warm-up: no report. HIIT: 7∗(2 min 70%-100% MIP training and 1 min 30% MIP active recovery). Cool down: no report. | 21 min/day, 3 times/week, 8 weeks | ① |
| Košuta 2024^19^ | Slovenia | CAD | MICT: 58(43/15) | MICT: 57±9.0 | 59(51/8) | 55±11.5 | Mode: stationary bike.  Warm-up: 5 min 50% VO_2peak_. HIIT: 7∗(1.5 min 90% VO_2peak_ cycling and 3 min 65% VO_2peak_ active recovery). Cool down: 5 min 50% VO_2peak_. | 42 min, acute exercise | ④⑤ |
| Aghaei Bahmanbeglou 2019^20^ | Iran | Stage 1 Hypertension | UC: 10(-) | UC: 47.0 ± 3.0 | SDHIIT: 10(-), LDHIIT: 10(-) | SDHIIT: 48±5, LDHIIT: 48.8±5 | Mode: programmable treadmill.  Warm-up: 5 min stretching exercises, walking and jogging. SDHIIT: 27∗(30s 80%-100% VO_2peak_ exercise and 30s 10%-20% VO_2peak_ active recovery). LDHIIT: 4∗(4min 75%-90% VO_2peak_ exercise and 4min 15%-30% VO_2peak_ active recovery). Cool down: 5 min. | SDHIIT: 37 min/day, 3 times/week, 8 weeks LDHIIT: 37 min/day, 3 times/week, 8 weeks | ① |
| Sosner 2019^21^ | France | HBP | MICT: 14(8/6) | MICT: 65 ± 6 | HIIT-immersed: 14(5/9), HIIT-dryland: 14(9/5) | HIIT-immersed: 63 ± 9, HIIT-dryland: 65 ± 8 | Mode: cycle ergometer.  Warm-up: 5 min 50% PPO. HIIT-immersed: 40∗(15 s 100% PPO cycling and 15 s passive recovery). Cool down: 5 min 50% PPO. Warm-up: 5 min 40 rpm pedalling. HIIT-dryland: 4­∗(15 s 100% PPO cycling and 15 s passive recovery). Cool down: 5 minute passive recovery period in a sitting position. | 34 min/day, 3 times/week, 2 weeks | ① |
| Alis 2015^22^ | Spain | healthy male | 10(10/0) | 25.80±3.39 | (10/0) | 25.80±3.39 | Mode: treadmill.  Warm-up: 2 min 60% HR_max_. HIIT: 5∗(2 min 90% HR_max_ running and 2 min 60% HR_max_ active recovery). Cool down: no report. | 24 min, acute exercise | ④ |
| Ghasemi 2019^23^ | Iran | overweight women | UC: 10(0/10) | UC: 21.06±2.65 | HIIT + green tea: 10(0/10), HIIT + placebo: 10(0/10) | HIIT + green tea: 22.47±3.32, HIIT + placebo: 23.58±2.23 | Mode: 40 m shuttle run.  Warm-up: no report. HIIT: 16∗(30 s 85%-95% HR_max_ and 30 s active recovery). Cool down: no report | 28 min/day, 3 times/week, 10 weeks | ④ |
| Sheykhlouvand 2018^24^ | Iran | professional canoe polo athletes | MICT: 7(7/0) | 24±3 | variable intensity HIIT: 7(7/0), variable volume HIIT: 7(7/0) | 24±3 | Mode: padding.  Warm-up: 5 min low-intensity paddling. VIHIIT: 6∗(1 min 100-130% VO_2peak_ paddling and 3min active recovery). VVHIIT: (6-9)∗(1 min 100% VO_2peak_ paddling and 3min active recovery). Cool down: no report. | 40-50 min/day, 3 times/week, 3 weeks | ③ |
| Whittaker 2013^25^ | Australia | nonsmoking male subjects | UC-Placebo: 3(3/0), UC-CAF, caffeine: 3(3/0) | 12total: 22.7±2.5 | HIIT + Placebo: 3(3/0), HIIT + CAF: 3(3/0) | 12total: 22.7±2.5 | Mode: electronically braked cycle ergometer.  Warm-up: 10 min 50% VO_2peak_. HIIT: 8∗(5 min 80% VO_2peak_ and 1 min 50% VO_2peak_ active recovery ). Cool down: no report. | 140 ± 105 min/week, acute exercise | ③ |
| Wu 2017^26^ | China | healthy sedentary males | MICT: 15(15/0), UC: 15(15/0) | MICT: 22.1±0.5, UC: 21.9±0.7 | 15(15/0) | 22.2±0.4 | Mode: stationary bicycle ergometer.  Warm-up: 3 min 30% VO_2max_. HIIT: 5∗(3 min 80% VO_2max_ and 3 min 40% VO_2max_ active recovery). Cool down: 3 min 30% VO_2max_. | 36 min/day, 5 times/week, 6 weeks | ③ |
| Adams 2017^27^ | Canada | TCS | UC: 28(28/0) | UC: 43.3±9.9 | 35(35/0) | 44±11.6 | Mode: uphill treadmill walking/running.  Warm-up: 5 min uphill treadmill walking/running at ±5% VT. HIIT: 4∗(4 min 75%-95 VO_2peak_ and 3 min 5% VT active recovery ). Cool down: 5 min. | 35 min/day, 3 times/week, 12 weeks | ①⑧ |
| Battillo 2023^28^ | USA | Older Adults with Prediabetes | MICT: 14(3/11) | MICT: 62.1±2.2 | 14(3/11) | 60.4±2.0 | Mode: cycle ergometry.  Warm-up: no report. HIIT: 10∗(3 min 90% HR_peak_ and 3 min 50% HR_peak_). Cool down: no report. | 60 min/day, 7 times/week, 2 weeks | ② |
| Bellia 2017^29^ | Italy | T2D | UC: 11(7/4) | UC: 56.3±6.4 | 11(9/2) | 58.8±7.9 | Mode: treadmill.  Warm-up: 10 min 40-60% HR_max_. HIIT: 4∗(4 min 75-80% HR_max_ treadmill and 3 min 45-50% HRmax active recovery). Cool down: 10 min 40-50% HRmax. | 48 min/day, 3 times/week, 12 weeks | ①⑥ |
| Beltrami 2020^30^ | Switzerland | young and healthy participants | MICT: 13(-), UC: 11(-) | MICT: 27±6, UC: 27±6 | 11(-) | 25±3 | Mode: the SpiroTiger device.  Warm-up: 4 min of quiet sitting. HIIT: 6∗(1 min intense breathing with the SpiroTiger device followed by 1 min of rest). Cool down: no report. | 16 min/day, 3 times/week, 4 weeks | ① |
| Clark 2020^31^ | Australia | males with overweight or obesity | MICT: 12(12/0) | MICT; 26 ± 8 | 16(16/0) | 30 ± 6 | Mode: manual-resistance cycle ergometers or electronically-braked cycle ergometers.  Warm-up: 3 min 65% HR_max_. HIIT: 10∗(1 min 90%-100% HRmax and 1 min 15% W_max_ active recovery). Cool down: 2 min 15% W_max_. | 25 min/day, 3 times/week, 6 weeks | ①② |
| Cocks 2013^32^ | UK | sedentary males | MICT: 8(8/0) | MICT; 21 ± 2.8 | 8(8/0) | 22 ± 2.8 | Mode: electromagnetically braked cycle  ergometer.  Warm-up: no report. HIIT: （4-6）­∗(0.5 min 500 workload and 4.5 min active recovery). Cool down: no report. | 30-90 min/day, 3 times/week, 6 weeks | ①⑥ |
| Cocks 2016^33^ | UK | obese men | MICT: 8(8/0) | 26±5.66 | 8(8/0) | 24±5.66 | Mode: electromagnetically braked cycle  ergometer.  Warm-up: 2min 50 W. HIIT: (4-7)­∗(0.5 min 200% W_max_ and 2 min 30 W cycling active recovery ). Cool down: no report. | 12-20 min/day, 3 times/week, 4 weeks | ①⑥ |
| Cooke 2022^34^ | Australia | Individuals with Overweight and Obesity | UC(IF): 12(2/10) | 37 ± 5.9 | HIIT: 11(1/10), HIIT+IF: 11(3/8) | HIIT: 32 ± 8.3, HIIT+IF: 39 ± 6.8 | Mode: magnetically braked bicycle ergometer.  Warm-up: 3 min 50 watts of resistance. HIIT: (4-6)­∗(20 s 150% VO_2peak_ and 40s 50 watts active recovery bicycling ). Cool down: 3 min 50 watts of resistance. | 11 min/day, 3 times/week, 16 weeks | ① |
| Davoodi 2022^35^ | Iran | T2D | MICT: 16(10/6), UC: 15(8/7) | MICT: 54.93 ± 5.56, UC: 53.66 ± 6.05 | 16(9/7) | 52.50 ± 7.22 | Mode: bicycle ergometer.  Warm-up: 10 min 40% HR_max_. HIIT: 12∗(1.5 min 85–90% HR_max_ biking and 2 min 55–60% HR_max_ active recovery). Cool down: 10 min 40% HR_max_. | 62 min/day, 3 times/week, 12 weeks | ⑦ |
| Deiseroth 2019^36^ | Switzerland | Sedentary elderly at risk | UC: 30(11/19) | UC: 57 ± 6 | 38(20/18) | 58 ± 5 | Mode: nordic walking.  Warm-up: 16 min. HIIT: 4­∗(4 min 80-90% HR_max_ nordic walking and 3 min 60-70% HR_max_ active recovery). Cool down: 16 min. | 60 min/day, 3 times/week, 12 weeks | ① |
| Eichner 2018^37^ | USA | in adults with prediabetes | MICT: 17(4/13) | MICT: 61.3±9.0 | 14(3/11) | 60.4±7.7 | Mode: cycle ergometer.  Warm-up: no report. HIIT: 10­∗(3 min 90% peak HR cycling and 3 min 50% peak HR active recovery). Cool down: no report. | 60 min/day, 6 times/week, 2 weeks | ①② |
| Fernandes 2020^38^ | Brazil | Individuals With Parkinson’s Disease | MICT: 9 (6/3) | MICT: 69.8 ± 7.7 | 12 (7/5) | 67.6 ± 8.9 | Mode: jogging/running.  Warm-up: 4 min walking at 9 level of RPE. HIIT: 7­∗(1 minute of jogging/running at 15 to 17 level with 2 minutes of walking at 9 to 11 level of RPE). Cool down: no report. | 25 min/day, 3 times/week, 12 weeks | ① |
| Hanssen 2017^39^ | Switzerland | episodic migraineurs | MICT: 12(2/10), UC: 12(2/10) | MICT: 36.5 ± 8.7, UC: 37.3 ± 11.9 | 13(3/10) | 36.2 ± 10.7 | Mode: treadmill.  Warm-up : 400 m easy running followed by two skipping exercises. HIIT: 4­∗(1 min 90-95% HR_max_ treadmilling and 3 min 70% HR_max_ active recovery). Cool down: 400 m and stretching exercises. | 45 min/day, 2 times/week, 12 weeks | ①②⑥ |
| Hanssen 2018^40^ | Switzerland | Patients suffering from Unipolar Depression | MICT: 15(4/11) | MICT: 38.1±12.2 | 19(5/14) | 37.5±10.1 | Mode: bicycle.  Warm-up: 5 min. HIIT: 25 ­∗(0.5 min 80%VO_2max_ and 0.5 min complete rest). Cool down: 5 min. | 35 min/day, 3 times/week, 4 weeks | ①②⑥ |
| Heydari 2013^41^ | Australia | young males | UC: 18(18/0) | 24.9±4.3 | 20(20/0) | 24.9±4.3 | Mode: cycle ergometer.  Warm-up: 5 min. HIIT:60∗(8 s 80-90 % HR_max_ cycling and 12 s active recovery). Cool down: 5 min. | 30 min/day, 3 times/week, 12 weeks | ② |
| Ho 2020^42^ | Australia | postmenopausal women | UC: 30(0/30) | UC: 53.2±3.07 | 30(0/30) | 53.9±3.39 | Mode: cycle ergometer.  Warm-up: 5 min 50-60 RPM. HIIT: 40­∗(20 s 120 RPM cycling and 10 s active recovery). Cool down: 5 min 50-60 RPM. | 30 min/day, 3 times/week, 8 weeks | ② |
| Kim 2017^43^ | USA | sedentary older adults, free of overt major clinical disease | MICT: 18(8/10), UC: 14(4/10) | MICT: 65±7.21, UC: 63±6.63 | 17(4/13) | 65±3.74 | Mode: an all-extremity non–weight-bearing air-braked ergometer  Warm-up: 10 min 70% HR_peak_. HIIT: 4­∗4 min 90% HR_peak_ and 3­∗3 min 70% HR_peak_ active recovery. Cool down: 5 min 70% HR_peak_. | 40 min/day, 4 times/week, 8 weeks | ① |
| Magalhães 2019^44^ | Portugal | T2D | MICT+RT: 16(7/9), UC: 22(11/11) | MICT+RT: 60.4±6.8, UC: 60.8±7.5 | 13(9/4) | 58.9±7.5 | Mode:cycling.  HIIT+RT:  Warm-up: no report. HIIT: 17∗(1 min 90% HRR and 1 min 40-60% HRR active recovery). Cool down: no report.  RT:1 set of 10–12 repetitions of upper and lower limbs exercises. | 34 min/day, 3 times/week, 1 year | ① |
| Magalhães 2021^45^ | Portugal | T2D | MICT+RT: 20(8/12), UC: 22(11/11) | MICT+RT: 57.5 ± 8.9, UC: 60.8 ± 7.5 | 20(9/11) | 59.6 ± 7.0 | Mode:cycling.  HIIT+RT:  Warm-up: no report. HIIT: 17∗(1 min 90% HRR and 1 min 40-60% HRR active recovery). Cool down: no report.  RT:1 set of 10–12 repetitions of upper and lower limbs exercises. | 34 min/day, 3 times/week, 1 year | ① |
| Okamoto 2019^46^ | Japan | older adults | MICT: 24(14/10) | MICT: 73±1 | 22(11/11) | 72±1 | Mode: wore a equipped with the accelerometer and a Pedometer.  Warm-up: no report. HIIT: 5­∗(3 min >70% VO_2peak_ walking and 3 min 40% VO_2peak_ active recovery). Cool down: no report. | Step per day (steps): 4955 ± 205, 30 min/day, 3-4 times/week, 20 weeks | ① |
| Petrick 2021^47^ | Canada | overweight/obese males | MICT: 11(11/0) | 37.4±15.1 | 12 (12/0) | 37.4±15.1 | Mode: cycle ergometers.  Warm-up:3 min 50 W. HIIT: 6­∗(0.5 min 75-90% HR_max_ and 2 min 50 W active recovery). Cool down: 2 min 50 W. | 20 min/day, 3 times/week, 6 weeks | ①⑦⑧ |
| Rakobowchuk 2008^48^ | Canada | healthy humans | MICT: 10(5/5) | 23.0 ± 2.4 | 10(5/5) | 23.6 ± 3.2 | Mode: cycle ergometers.  Warm-up: no report. HIIT: 6­∗(0.5 min Wingate tests and 4 min 30 W active recovery). Cool down: no report. | 27 min/day, 3 times/week, 6 weeks | ⑦⑧ |
| Ramírez-Vélez 2019^49^ | Colombia | physically inactive adults | MICT: 9(-) | 18–45 yrs | 11(-) | 18–45 yrs | Mdoe: walking on a treadmill.  Warm-up: 10 min 60-70% HR. HIIT: 4­∗(4 min85-95% HR and 4 min 75-85% active recovery). Cool down: 4 min 60-70% HR. | 42 min/day, 3 times/week, 12 weeks | ①②⑦⑧ |
| Ramírez-Vélez 2020^50^ | Colombia | Overweight Adults | UC: 16(-) | 40.78±7.06 | HIIT-1:14(-), HIIT-2: 15(-) | 40.78±7.06 | Mdoe: treadmill.  Warm-up: 5 min. HIIT-1: 4­∗(4 min 85-95% HR_max_ and 4 min 65% HR_max_ active recovery). Cool down: 4 min 65% HR_max_.  Warm-up: 10 min of walking and light. HIIT-2: 20min (60s recovery and 50-70% 1RM).  Cool down: 10 min of relaxation and stretching exercises. | 41 min/day, 3 times/week, 12 weeks | ①②⑦⑧ |
| Scott 2019a^51^ | UK | adults with elevated CVD risk | MICT: 13(4/9) | MICT: 38 ± 9 | HIIT-home: 9(4/5), HIIT-lab: 10(5/5) | HIIT-home: 32 ± 8, HIIT-lab: 37 ± 13 | Mode: electromagnetically-braked cycle ergometer.  Warm-up: no report. HIIT-home: 8­∗(1 min ≥80% HRmax body weight exercises and 1 min active recovery). HIIT-lab: 8­∗(1 min 100% Wmax cycling and 1 min active recovery). Cool down: no report. | 16 min/day, 3 times/week, 12 weeks | ①⑦ |
| Scott 2019b^52^ | UK | T1D | MICT: 7(5/2) | MICT: 29 ± 13.23 | 7(5/2) | 29 ± 7.94 | Mode: cycle ergometer.  Warm-up: 3 min low intensity. HIIT: 10∗(1 min 100% VO_2_peak cycling and 1 min 50W active recovery). Cool down: no report. | 23 min/day, 3 times/week, 6 weeks | ① |
| Soltani 2021^53^ | Iran | sedentary adult men. | MICT: 15(15/0), UC: 15(15/0) | MICT: 42.5 ± 6.2, UC: 41.5 ± 5.6 | 15(15/0) | 42.2 ± 5.3 | Mode: running.  HIIT: at 85% of VO_2_max for a 40-min duration. | 25-40 min/day, 3 times/week, 12 weeks | ① |
| Tang 2022^54^ | China | inactive adults | MICT: 13(4/9) | MICT: 39.2 ± 6.0 | 13(3/10) | 42.2 ± 5.7 | Mode: swimming.  Warm-up: 15 min 50-55% HRmax on land and 10 min 50-55% HRmax aquatic exercise. HIIT: 12­∗(0.5 min 95% HR_max_ and 1 min 80% HR_max_ active recovery). Cool down: 10 minute. | 53 min/day, 3 times/week, 6 weeks | ⑥⑦ |
| Tangchaisuriya 2022^55^ | Thailand | male masters road cyclists | MICT: 16(16/0) | MICT: 41.5 ± 4.5 | HIIT: 17(17/0), BFRIT: 17(17/0) | HIIT: 41.7 ± 3.8, BFRIT: 39.6 ± 3.8 | Mode: cycling.  Warm-up: 6 min of cycling at 25% PPO, 2 min at 50% PPO, and 2 min at 60% PPO. HIIT: 4∗(4 min 80% PPO and 2 min 30% PPO active recovery). Cool down: 10 min 25% PPO cycling. BFRIT: 2*(4 min 80% PPO and 2 min 30% PPO active recovery, 4 min 60% PPO and 2 min 30% PPO active recovery), with BFR cuffs during exercise, cadence 85-90 rpm. | 45 min/day, 2 times/week, 12 weeks | ⑦ |
| Way 2020^56^ | Australia | inactive adults with obesity and T2D | MICT: 12(6/6), UC: 11(7/4) | MICT: 54.8 ± 2.4, UC: 51.9 ± 1.4 | 12 (7/5) | 56.9 ± 2.1 | Mode: cycling ergometer.  Warm-up: 10 min 50% VO2_peak_. HIIT: 1*(4 min 90% VO_2peak_). Cool down: 5 min 50% VO_2peak_. | 19 min/day, 3 times/week, 12 weeks | ①②⑥ |
| Hanssen 2015^57^ | Switzerland | healthy young men | MICT: 7(7/0) | 19-32 yrs | 7(7/0) | 19-32 yrs | Mode: treadmill.  Warm-up: 10 min 70% HRmax. HIIT: 4­∗(4 min 90-95% HR_max_ and 3 min 70% HR_max_ active recovery). Cool down: 3 min 70% HR_max_. | 40 min, acute exercise | ②⑥ |
| Marcal 2021^58^ | Brazil | sedentary or insufficiently active individuals | 12(3/9) | 21士2 | 12(3/9) | 21士2 | Mode: treadmill.  Warm-up: 4 min 50% HRR walking. HIIThr: 7∗(1 min 85% HRR jogging/running and 2 min 50% HRR active recovery). Cool down: no report. Warm-up: 4 min 9 RPE. HIITrpe: 7∗(1 min 15-17RPE jogging/running and 2 min 9-11 RPE active recovery). Cool down: no report. | 25 min, acute exercise | ① |
| Perissiou 2018^59^ | Australia | older adults | 17(14/3) | 71 | 17(14/3) | 71 | Mode: cycling.  Warm-up: no report. HIIT: 12­∗(1 min 70% PPO cycling and 1 min 10% PPO active recovery). Cool down: no report. | 24 min, acute exercise | ①⑥ |
| Sapp 2020^60^ | USA | healthy young men | MICT: 10(10/0) | 22 ± 2 | 10(10/0) | 22 ± 2 | Mode:a leg cycle ergometer.  Warm-up: 6 min 60% PPO. HIIT: 4­∗(3 min 85% PPO and 4 min 40% PPO active recovery). Cool down: no report. | 34 min, acute exercise | ①②⑥ |
| Siasos 2016a^61^ | Athens | healthy men | MICT: 20(20/0) | 22.6 ± 3.3 | 20(20/0) | 22.6 ± 3.3 | Mode: cycling ergometer.  Warm-up: 3 min without resistance exercise. HIIT: 30­∗(0.5 min 100% max aerobic work and 0.5 min active recovery). Cool down: no report. | 33 min, acute exercise | ② |
| Siasos 2016b^62^ | Athens | healthy men | MICT: 20(20/0) | 22.6 ± 3.3 | 20(20/0) | 22.6 ± 3.3 | Mode: cycling ergometer.  Warm-up: 3 min without resistance exercise. HIIT: 30­∗(0.5 min 100% max aerobic work and 0.5 min active recovery). Cool down: no report. | 33 min, acute exercise | ①⑦ |
| Viana 2018^63^ | Brazil | T2D | 11(2/9) | 52.3 ± 3.0 | 11(2/9) | 52.3 ± 3.0 | Mode: treadmill.  Warm-up: 4 min 50% HRR walking. HIIT-hr: 7∗(1 min 85% HRR jogging/running and 2 min 50% HRR active recovery). Cool down: no report. Warm-up: 4 min 9 RPE. HIIT-rpe: 7­∗(1 min 15-17RPE jogging/running and 2 min 9-11 RPE active recovery). Cool down: no report. | 25 min, acute exercise | ① |
| Melo 2024^64^ | Portugal | adults with intellectual and developmental disability | MICT: 9(5/4) | 29±13 | 8(6/2) | 26±8 | Mode: at home (such as jumping jacks, standing box, side shift with floor touch, high knees, half burpee, and hook box were used) or at the gym (cycle ergometers).  HIIT+RE:  Warm-up: 5 min. HIIT: 15­∗(20s 95% HR_max_ and 45s 45% HR_max_ active recovery). Cool down: 3 min.  RE: at 2 x 12 repetitions at RPE OMNI-RES 8 | 25 min/day, 3 times/week, 8 weeks | ① |
| Cox 2024^65^ | Australia | T2D | MICT: 23(14/9), UC: 23(14/9) | MICT: 60.1±7.3, UC: 59.4±10.2 | 23(14/9) | 59.0±8.8 | Mode: treadmill, upright bike, recumbent bike.  Warm-up: 3 min 60% HR_peak_. HIIT: 4 min at 85-95% HR_peak_. 8­∗(1 min 90% HR_peak_ and 1 min rest). Cool down: 3 min 60% HR_peak_. | 26 min/day, 3 times/week, 8 weeks | ①②⑥⑦ |
| Huang 2025^66^ | China | Sedentary young individuals | MICT: 15(9/6), UC: 16(9/7) | MICT: 21.80±1.78, UC: 22.19±1.52 | 16(8/8) | 21.44±1.93 | Mode: cycling ergometer.  Warm-up: 2 min 50% VO_2max_. HIIT: 10­∗(60s 90% VO_2max_ and 60s 50% VO_2max_ recovery). Cool down: 3 min 50% VO_2max_. | 25 min/day, 3 times/week, 8 weeks | ①②⑦ |
| Moncion 2024^67^ | Canada | After stroke | MICT: 40(23/17) | MICT: 64.4±9.7 | 42(27/15) | 65.4±8.9 | Mode: treadmill.  Warm-up: no report. HIIT: 10×(60s 80–100% HRR and 60s 30% HRR recovery). Cool down: no report. | 19 min/day, 3 times/week, 12 weeks | ① |
| Zarekar 2024^68^ | Iran | lymphoma and multiple myeloma patients | UC: 10(5/5) | UC: 33.1±9.6 | 10(6/4) | 32.2±11.6 | Mode: cycling.  Warm-up: 5 min 10-20% peak power. HIIT: 12­∗(60s 100% peak power and 60s active rest at 20% of peak power). Cool down: no report. | 29min, acute exercise | ③ |

M: male, F: female, FH+: hypertensive parents, cfPWV: carotid-femoral pulse wave velocity, HBP: hypertension, CHF: chronic heart failure, HTx: heart transplantation, AIx: augmentation index, CABG: coronary artery bypass grafting, PCI: percutaneous coronary interventions, PLT: platelet count, FIB: fibrinogen, D-D: D-dimer, VO_2max_: maximal oxygen consumption, WR_peak_: peak work rate, VO_2peak_: peak oxygen uptake, MICT: moderate intensity continuous training, MHRR: maximal heart rate reserve, HR_MAX_: maximal heart rate, AIx@75HR: AIx normalized to a heart rate of 75 beats·min^−1^, ToF: tetralogy of fallot, FMD = flow-mediated dilation, CHD: coronary heart disease, P_max_: maximal power output, CAD: coronary artery disease, HF: heart failure, RPE: rating of perceived exertion, HFpEF: heart failure with preserved ejection fraction, MIP, maximal inspiratory pressure, TCS: testicular cancer survivors, nFMD: brachial flow-mediated dilation normalized, RPM: revolutions per minute, RT: resistance exercise, PPO: peak power output, T2DM: type 2 diabetes mellitus, T1DM: type 1 diabetes mellitus, AE: aerobic exercise, HIIT: high-intensity interval training, MHR: maximum heart rate, HRR: heart rate reserve, HR peak: peak heart rate, MVV: maximal voluntary ventilation, VC: vital capacity, ①cfPWV, ②AIx, ③PLT, ④FIB, ⑤D-D, ⑥AIx@75HR, ⑦FMD, ⑧nFMD

| **Study** | **Details of interventions in the control group (UC)** | **Details of interventions in the control group (MICT)** |
| --- | --- | --- |
|  |  |  |
| Ciolac 2010 | Without exercise training | Warm-up: no report. MICT: 40 min of walking at 60–70% of VO_2MAX_. Cool down: no report. |
| Guimara˜es 2010 | Maintain their daily activities without exercise training | Warm-up: no report. MICT: 40 min on a treadmill at 60% of reserve heart rate. Cool down: no report. |
| Chrysohoou 2015 | Received standard heart failure management by the attending physician in the Heart Failure Unit, with no structured exercise protocol provided | - |
| Dall 2015 | - | Warm-up: no report. MICT: 45 min of biking at 60–70% of VO_2peak_. Cool down: no report. |
| Ahmadizad 2017 | Remained seated with no activity for the same period of time | Warm-up: 5 min. MICT:30 min of treadmill at 60–70% of VO_2peak_. Cool down: 5 min. |
| Valizadeh 2017 | Medical care | - |
| Mora-Rodriguez 2018 | Remain sedentary | - |
| Novakovi´c 2018 | Continue with regular physical activities. | Warm-up: 8 min 50% HR_peak_. MICT: 26 min of cycling at 70% HR_peak_. Cool down: 5 min 50% HR_peak_. |
| Sobhani 2018 | - | Warm-up: no report. MICT: 40 minutes of running on a treadmill with 65% of HR_max_. Cool down: no report. |
| Heber 2020 | - | Warm-up: no report. MICT: 45 min at 60% of of P_max_ bicycle. Cool down: no report. |
| Soltani 2020 | Not to engage in any regular exercise training | - |
| Taylor 2022 | - | Warm-up: no report. MICT: 40 min exercise at 65–75%HR_peak_. Cool down: no report. |
| Hearon 2022 | Permitted to perform any combination of yoga, balance, or strength training | - |
| Kristiansen 2022 | Standard care | - |
| Marçal 2022 | 30 min of sitting on a submerse chair resting (attached to a fixed platform) | Warm-up: 4 min RPE 9 walking. MICT: 26 min of walking at 11-13 RPE. Cool down: no report. |
| Avazpour 2023 | Without exercise training | - |
| Gevaert 2023 | - | Warm-up: no report. MICT: 40 min in cardiac rehabilitation centers at 35-50% of reserve heart rate. Cool down: no report. |
| Tanriverdi 2023 | The inspiratory load was fixed at 0 cmH_2_O (unloaded), with 7 cycles per session (2-min training + 1-min rest per cycle; total 21 min). | - |
| Košuta 2024 | - | Warm-up: 5 min 50% HR_peak_. MICT: 32 min of cycling at 75% HR_peak_. Cool down: 3 min 50% HR_peak_. |
| Aghaei Bahmanbeglou 2019 | Participated in no regular exercise training | - |
| Sosner 2019 | - | Warm-up: 5 min cycling at 50W . MICT: 24 min of cycling at 50% PPO. Cool down: 5 min recovery in a sitting position. |
| Alis 2015 | - | Warm-up: no report. MICT: 60 min of running at 55% VO_2max_. Cool down: no report. |
| Ghasemi 2019 | Green tea | - |
| Sheykhlouvand 2018 | - | Warm-up: no report. MICT: 60 min of paddling at 75% VO_2peak_. Cool down: no report. |
| Whittaker 2013 | Rest PL: seated rest for 90 minutes, followed by placebo administration.  Rest CAF: seated rest for 90 minutes, followed by caffeine administration. | - |
| Wu 2017 | Without any exercise | Warm-up: 3 min 30% VO_2_max. MICT: 30 min of cycling at 60% VO_2max_. Cool down: 3 min 30% VO_2max_. |
| Adams 2017 | Usual care (maintain other low-intensity to moderate-intensity exercise) | - |
| Battillo 2023 | - | Warm-up: no report. MICT: 60 min of cycling at 70% HR_peak_. Cool down: no report. |
| Bellia 2017 | SOC (unsupervised physical activity) | - |
| Beltrami 2020 | A 15-minute quiet sitting session with no respiratory interventions | Warm-up: no report. MICT: 15 min of continuous normocapnic hyperpnea (60% MVV) via SpiroTiger, fixed tidal volume (50-60% VC) and breathing frequency with feedback. Cool down: no report. |
| Clark 2020 | - | Warm-up: no report. MICT: 30 min of cycling at 65-75% HR_max_. Cool down: no report. |
| Cocks 2013 | - | Warm-up: no report. MICT: 40-60 min of cycling at 65 VO_2peak_. Cool down: no report. |
| Cocks 2016 | - | Warm-up: no report. MICT: 40-60 min of cycling at 65 VO_2peak_. Cool down: no report. |
| Cooke 2022 | 5:2 IF(restricted energy intake for two non-consecutive days per week) | - |
| Davoodi 2022 | Usual activities without any regular training | Warm-up: no report. MICT: 42 min of cycling at 70 HR_MAX_. Cool down: no report. |
| Deiseroth 2019 | Receiving standard PA recommendations only | - |
| Eichner 2018 | - | Warm-up: no report. MICT: 60 min of cycling at 70 HR_peak_. Cool down: no report. |
| Fernandes 2020 | - | Warm-up: 4 min walking at 9 level of RPE. MICT: 26 minute of jogging/running at 11 to 14 level of RPE). Cool down: no report. |
| Hanssen 2017 | Requested to maintain their habitual daily physical activity profile and received additional standard physical activity recommendations. | Warm-up: 400 m easy running followed by two skipping exercises. MICT: 45 min of cycling at 70 HR_peak_. Cool down: 400 m and stretching exercises. |
| Hanssen 2018 | - | Warm-up: 5 min. MICT: 20 min of cycling at 60%VO_2max_. Cool down: 5 min. |
| Heydari 2013 | Maintain their normal daily routine | - |
| Ho 2020 | Maintain their previous levels of physical activity | - |
| Kim 2017 | Without exercise training | Warm-up: 10 min 70% HR_peak_. MICT: 47min at 70% HR_peak_. Cool down: 5 min 70% HR_peak_. |
| Magalhães 2019 | Non-exercise intervention (standard counseling with guidance on general physical activity) | MICT+RT: Warm-up: no report. MICT: 40-60 min of cycling at 40-60% HRR. Cool down: no report.RT:1 set of 10–12 repetitions of upper and lower limbs exercises. |
| Magalhães 2021 | Non-exercise intervention (standard counseling with guidance on general physical activity) | MICT+RT: Warm-up: no report. MICT: 40-60 min of cycling at 40-60% HRR. Cool down: no report.RT:1 set of 10–12 repetitions of upper and lower limbs exercises. |
| Okamoto 2019 | - | Warm-up: no report. MICT: walking 8000 steps at 50% VO_2peak_. Cool down: no report. |
| Petrick 2021 | - | Warm-up: no report. MICT: 40min of cycling at 58% W_peak_. Cool down: no report. |
| Rakobowchuk 2008 | - | Warm-up: no report. MICT: 60min of cycling at 65% VO_2peak_. Cool down: no report. |
| Ramírez-Vélez 2019 | - | Warm-up: 10 min 60-70% HR. HIIT: 35 min of cycling at 60-70% HR . Cool down: 4 min 60-70% HR. |
| Ramírez-Vélez 2020 | Without exercise training | - |
| Scott 2019a | - | Warm-up: no report. MICT: 50min of (swimming, cycling or walking/running) at 65% HRmax. Cool down: no report. |
| Scott 2019b | - | Warm-up: no report. MICT: 50min of cycling at 50% VO_2peak_. Cool down: no report. |
| Soltani 2021 | Maintain daily routines | Warm-up: no report. MICT: 45-60min of running at 50-60% VO_2max._ Cool down: no report. |
| Tang 2022 | - | Warm-up: 15 min 50-55% HRmax on land and 10 min 50-55% HRmax aquatic exercise. MICT: 30 min at 70-75% HR_max_. Cool down: 10 minute. |
| Tangchaisuriya 2022 | - | Warm-up: 6 min of cycling at 25% PPO, 2 min at 50% PPO, and 2 min at 60% PPO. MICT: 75min of cycling at 65-70% PPO. Cool down: 10 min 25% PPO cycling. |
| Way 2020 | Static stretching of the muscle | Warm-up: 10 min 50% VO_2peak_. MICT: 45 min of cycling at 60% VO_2peak_. Cool down: 5 min 50% VO_2peak_. |
| Hanssen 2015 | - | 80% HR anaerobic threshold |
| Marcal 2021 | 25 min of resting quietly | - |
| Perissiou 2018 | Without exercise training | Warm-up: no report. MICT: 24min of cycling at 70% PPO. Cool down: no report. |
| Sapp 2020 | - | Warm-up: no report. MICT: 30min of cycling at 60% PPO. Cool down: no report. |
| Siasos 2016a | - | Warm-up: 3 min without resistance exercise. MICT: 30 min of cycling at 50% max aerobic work. Cool down: no report. |
| Siasos 2016b | - | Warm-up: 3 min without resistance exercise. MICT: 30 min of cycling at 50% max aerobic work. Cool down: no report. |
| Viana 2018 | 30 min of resting quietly in the seated position | Warm-up: 4 min of walking at 9 RPE. MICT: 26 min of walking/jogging at 11-14 level of RPE. Cool down: no report. |
| Melo 2024 | - | Warm-up: 5 min. MICT: 3­∗(5-10 min 55-85% HRR). Cool down: 3 min.  RE: at 2 x 12 repetitions at RPE OMNI-RES 8. |
| Cox 2024 | Maintain their usual physical activity and dietary habits | MICT+RE: 52min.  MICT: 22 min and 30 s of aerobic exercise at 55–69% of HR_peak_.  RE: 30 min of resistance-based exercises at a moderate intensity (RPE 11–13; fairly light to somewhat hard) |
| Huang 2025 | Maintain usual sedentary behavior | Warm-up: 2 min 20% VO_2max_. MICT: 35 min of cycling at 60% VO_2max_. Cool down: 3 min 20% VO_2max_. |
| Moncion 2024 | - | Warm-up: no report. . MICT: 20–30 min 40–60% HRR (progressed by 10% HRR and 5 min every 4 weeks). Cool down: no report. |
| Zarekar 2024 | Seated for the same duration without any physical activity | - |

**Appendix 6.** List of included studies**.**

1. Ciolac, E. G. et al. Effects of high-intensity aerobic interval training vs. moderate exercise on hemodynamic, metabolic and neuro-humoral abnormalities of young normotensive women at high familial risk for hypertension. Hypertens. Res. 33, 836–843 (2010).

2. Guimarães, G. V. et al. Effects of continuous vs. interval exercise training on blood pressure and arterial stiffness in treated hypertension. Hypertens. Res. 33, 627–632 (2010).

3. Chrysohoou, C. et al. Cardiovascular effects of high-intensity interval aerobic training combined with strength exercise in patients with chronic heart failure. A randomized phase III clinical trial. Int. J. Cardiol. 179, 269–274 (2015).

4. Dall, C. H. et al. Effect of moderate- versus high-intensity exercise on vascular function, biomarkers and quality of life in heart transplant recipients: A randomized, crossover trial. J. Heart Lung Transplant. 34, 1033–1041 (2015).

5. Ahmadizad, S. et al. Platelet activation and function in response to high intensity interval exercise and moderate continuous exercise in CABG and PCI patients. Clin. Hemorheol. Microcirc. 64, 911–919 (2017).

6. Valizadeh, R., Nikbakht, H., Ghazalian, F., Abednatanzi, H. & Costill, D. L. The Effect of High Intensity Interval Training on the Response of Coagulation and Fibrinolytic Factors of Hypertensive Patients to One Bout Submaximal Endurance Exercise.

7. Mora‐Rodriguez, R. et al. Effects of aerobic interval training on arterial stiffness and microvascular function in patients with metabolic syndrome. J. Clin. Hypertens. 20, 11–18 (2018).

8. Novaković, M. et al. Exercise training in adults with repaired tetralogy of Fallot: A randomized controlled pilot study of continuous versus interval training. Int. J. Cardiol. 255, 37–44 (2018).

9. Sobhani, V., Taghizadeh, M. & Moshkani-Farahani, M. Platelet indices and function response to two types of high intensity interval exercise and comparison with moderate intensity continuous exercise among men after coronary artery bypass graft: A randomized trial. ARYA Atheroscler. 14, (2018).

10. Heber, S. et al. Effects of high-intensity interval training on platelet function in cardiac rehabilitation: a randomised controlled trial. Heart 106, 69–79 (2020).

11. Soltani, M., Aghaei Bahmanbeglou, N. & Ahmadizad, S. High-intensity interval training irrespective of its intensity improves markers of blood fluidity in hypertensive patients. Clin. Exp. Hypertens. 42, 309–314 (2020).

12. Taylor, J. L. et al. Comparison of high intensity interval training with standard cardiac rehabilitation on vascular function. Scand. J. Med. Sci. Sports 32, 512–520 (2022).

13. Hearon, C. M. et al. 1 Year HIIT and Omega-3 Fatty Acids to Improve Cardiometabolic Risk in Stage-A Heart Failure. JACC Heart Fail. 10, 238–249 (2022).

14. Kristiansen, J. et al. Haemostasis and fibrinolysis after regular high-intensity interval training in patients with coronary artery disease: a randomised controlled trial. Open Heart 9, e002127 (2022).

15. Roque Marçal, I. et al. Acute high-intensity interval exercise versus moderate-intensity continuous exercise in heated water-based on hemodynamic, cardiac autonomic, and vascular responses in older individuals with hypertension. Clin. Exp. Hypertens. 44, 427–435 (2022).

16. Avazpour, S., Amini, A., Shirvani, H. & Arabzadeh, E. Exercise modulation in inflammation and metabolic hormonal disorders of COVID-19 to decrease risk factors in coronary heart disease. Horm. Mol. Biol. Clin. Investig. 44, 199–206 (2023).

17. Gevaert, A. B. et al. Effect of Training on Vascular Function and Repair in Heart Failure With Preserved Ejection Fraction. JACC Heart Fail. 11, 454–464 (2023).

18. Tanriverdi, A. et al. Effects of high intensity interval-based inspiratory muscle training in patients with heart failure: A single-blind randomized controlled trial. Heart Lung 62, 1–8 (2023).

19. Košuta, D., Novaković, M., Božič Mijovski, M. & Jug, B. Acute effects of high intensity interval training versus moderate intensity continuous training on haemostasis in patients with coronary artery disease. Sci. Rep. 14, 1963 (2024).

20. Aghaei Bahmanbeglou, N., Ebrahim, K., Maleki, M., Nikpajouh, A. & Ahmadizad, S. Short-Duration High-Intensity Interval Exercise Training Is More Effective Than Long Duration for Blood Pressure and Arterial Stiffness But Not for Inflammatory Markers and Lipid Profiles in Patients With Stage 1 Hypertension. J. Cardiopulm. Rehabil. Prev. 39, 50–55 (2019).

21. Sosner, P. et al. Ambulatory blood pressure reduction following 2 weeks of high-intensity interval training on an immersed ergocycle. Arch. Cardiovasc. Dis. 112, 680–690 (2019).

22. Alis, R., Ibañez-Sania, S., Basterra, J., Sanchis-Gomar, F. & Romagnoli, M. Effects of an acute high-intensity interval training protocol on plasma viscosity. J. Sports Med. Phys. Fitness 55, 647–653 (2015).

23. Ghasemi, E. & Nayebifar, S. Benefits of 10 weeks of high-intensity interval training and green tea supplementation on cardiovascular risk factors and VO 2max in overweight women. J. Res. Med. Sci. 24, 79 (2019).

24. Sheykhlouvand, M. et al. Low-Volume High-Intensity Interval Versus Continuous Endurance Training: Effects on Hematological and Cardiorespiratory System Adaptations in Professional Canoe Polo Athletes. J. Strength Cond. Res. 32, 1852–1860 (2018).

25. Whittaker, J. P., Linden, M. D. & Coffey, V. G. Effect of Aerobic Interval Training and Caffeine on Blood Platelet Function. Med. Sci. Sports Exerc. 45, 342–350 (2013).

26. Wu, L.-H., Chang, S.-C., Fu, T.-C., Huang, C.-H. & Wang, J.-S. High-intensity Interval Training Improves Mitochondrial Function and Suppresses Thrombin Generation in Platelets undergoing Hypoxic Stress. Sci. Rep. 7, 4191 (2017).

27. Adams, S. C. et al. Effects of high‐intensity aerobic interval training on cardiovascular disease risk in testicular cancer survivors: A phase 2 randomized controlled trial. Cancer 123, 4057–4065 (2017).

28. Battillo, D. J. & Malin, S. K. Relation of Aortic Waveforms with Gut Hormones following Continuous and Interval Exercise among Older Adults with Prediabetes. Metabolites 13, 137 (2023).

29. Bellia, A. et al. Exercise individualized by TRIMPi method reduces arterial stiffness in early onset type 2 diabetic patients: A randomized controlled trial with aerobic interval training. Int. J. Cardiol. 248, 314–319 (2017).

30. Beltrami, F. G., Mzee, D. & Spengler, C. M. No Evidence That Hyperpnea-Based Respiratory Muscle Training Affects Indexes of Cardiovascular Health in Young Healthy Adults. Front. Physiol. 11, 530218 (2020).

31. Clark, T. et al. High-intensity interval training for reducing blood pressure: a randomized trial vs. moderate-intensity continuous training in males with overweight or obesity. Hypertens. Res. 43, 396–403 (2020).

32. Cocks, M. et al. Sprint interval and endurance training are equally effective in increasing muscle microvascular density and eNOS content in sedentary males. J. Physiol. 591, 641–656 (2013).

33. Cocks, M. et al. Sprint interval and moderate‐intensity continuous training have equal benefits on aerobic capacity, insulin sensitivity, muscle capillarisation and endothelial eNOS/NAD(P)Hoxidase protein ratio in obese men. J. Physiol. 594, 2307–2321 (2016).

34. Tchounwou, P. B. Environmental Research and Public Health. Int. J. Environ. Res. Public. Health 1, 1–2 (2004).

35. Davoodi, M. et al. Improved blood pressure and flow‐mediated dilatation via increased plasma adropin and nitrate/nitrite induced by high‐intensity interval training in patients with type 2 diabetes. Exp. Physiol. 107, 813–824 (2022).

36. Deiseroth, A. et al. Exercise and Arterial Stiffness in the Elderly: A Combined Cross-Sectional and Randomized Controlled Trial (EXAMIN AGE). Front. Physiol. 10, 1119 (2019).

37. Eichner, N. Z. M. et al. Postprandial augmentation index is reduced in adults with prediabetes following continuous and interval exercise training. Exp. Physiol. 104, 264–271 (2019).

38. Fernandes, B. et al. High-Intensity Interval Versus Moderate-Intensity Continuous Training in Individuals With Parkinson’s Disease: Hemodynamic and Functional Adaptation. J. Phys. Act. Health 17, 85–91 (2020).

39. Hanssen, H. et al. Superior Effects of High-Intensity Interval Training vs. Moderate Continuous Training on Arterial Stiffness in Episodic Migraine: A Randomized Controlled Trial. Front. Physiol. 8, 1086 (2017).

40. Hanssen, H. et al. Effects of Endurance Exercise Modalities on Arterial Stiffness in Patients Suffering from Unipolar Depression: A Randomized Controlled Trial. Front. Psychiatry 8, 311 (2018).

41. Heydari, M. et al. High-intensity intermittent exercise and cardiovascular and autonomic function. Clin Auton Res. 23, 57–65 (2013).

42. Ho, T. Y. et al. The effect of interval sprinting exercise on vascular function and aerobic fitness of post‐menopausal women. Scand. J. Med. Sci. Sports 30, 312–321 (2020).

43. Kim, H.-K. et al. All-Extremity Exercise Training Improves Arterial Stiffness in Older Adults. Med. Sci. Sports Exerc. 49, 1404–1411 (2017).

44. Magalhães, J. P. et al. Effects of combined training with different intensities on vascular health in patients with type 2 diabetes: a 1-year randomized controlled trial. Cardiovasc. Diabetol. 18, 34 (2019).

45. Magalhães, J. P. et al. Interindividual Variability in Fat Mass Response to a 1-Year Randomized Controlled Trial With Different Exercise Intensities in Type 2 Diabetes: Implications on Glycemic Control and Vascular Function. Front. Physiol. 12, 698971 (2021).

46. Okamoto, T., Hashimoto, Y. & Kobayashi, R. Effects of interval walking training compared to normal walking training on cognitive function and arterial function in older adults: a randomized controlled trial. Aging Clin. Exp. Res. 31, 1451–1459 (2019).

47. Petrick, H. L. et al. Endurance and Sprint Training Improve Glycemia and V˙O2peak but only Frequent Endurance Benefits Blood Pressure and Lipidemia. Med. Sci. Sports Exerc. 53, 1194–1205 (2021).

48. Rakobowchuk, M. et al. Sprint interval and traditional endurance training induce similar improvements in peripheral arterial stiffness and flow-mediated dilation in healthy humans. Am. J. Physiol.-Regul. Integr. Comp. Physiol. 295, R236–R242 (2008).

49. Ramírez-Vélez, R. et al. Effectiveness of HIIT compared to moderate continuous training in improving vascular parameters in inactive adults. Lipids Health Dis. 18, 42 (2019).

50. Ramírez-Vélez, R. et al. The Effect of 12 Weeks of Different Exercise Training Modalities or Nutritional Guidance on Cardiometabolic Risk Factors, Vascular Parameters, and Physical Fitness in Overweight Adults: Cardiometabolic High-Intensity Interval Training-Resistance Training Randomized Controlled Study. J. Strength Cond. Res. 34, 2178–2188 (2020).

51. Scott, S. N. et al. Home‐hit improves muscle capillarisation and eNOS/NAD(P)Hoxidase protein ratio in obese individuals with elevated cardiovascular disease risk. J. Physiol. 597, 4203–4225 (2019).

52. Scott, S. N. et al. High-Intensity Interval Training Improves Aerobic Capacity Without a Detrimental Decline in Blood Glucose in People With Type 1 Diabetes. J. Clin. Endocrinol. Metab. 104, 604–612 (2019).

53. Soltani, M. et al. Effect of Intensity on Changes in Cardiac Autonomic Control of Heart Rate and Arterial Stiffness After Equated Continuous Running Training Programs. Front. Physiol. 12, 758299 (2021).

54. Tang, S. et al. Effects of aquatic high-intensity interval training and moderate-intensity continuous training on central hemodynamic parameters, endothelial function and aerobic fitness in inactive adults. J. Exerc. Sci. Fit. 20, 256–262 (2022).

55. Tangchaisuriya, P., Chuensiri, N., Tanaka, H. & Suksom, D. Physiological Adaptations to High-Intensity Interval Training Combined with Blood Flow Restriction in Masters Road Cyclists. Med. Sci. Sports Exerc. 54, 830–840 (2022).

56. Way, K. L. et al. The effect of low-volume high-intensity interval training on cardiovascular health outcomes in type 2 diabetes: A randomised controlled trial. Int. J. Cardiol. 320, 148–154 (2020).

57. Hanssen, H. et al. Acute effects of interval versus continuous endurance training on pulse wave reflection in healthy young men. Atherosclerosis 238, 399–406 (2015).

58. Marçal, I. R. et al. Prescribing high-intensity interval exercise by rating of perceived exertion in young individuals. J. Sports Med. Phys. Fitness 61, 797–802 (2021).

59. Perissiou, M. et al. Effects of exercise intensity and cardiorespiratory fitness on the acute response of arterial stiffness to exercise in older adults. Eur. J. Appl. Physiol. 118, 1673–1688 (2018).

60. Sapp, R. M. et al. Changes in circulating microRNA and arterial stiffness following high‐intensity interval and moderate intensity continuous exercise. Physiol. Rep. 8, (2020).

61. Siasos, G. et al. The Acute Impact of Different Types of Aerobic Exercise on Arterial Wave Reflections and Inflammation. Cardiology 135, 81–86 (2016).

62. Siasos, G. et al. Acute effects of different types of aerobic exercise on endothelial function and arterial stiffness. Eur. J. Prev. Cardiol. 23, 1565–1572 (2016).

63. Viana, A. A., Fernandes, B., Alvarez, C., Guimarães, G. V. & Ciolac, E. G. Prescribing high-intensity interval exercise by RPE in individuals with type 2 diabetes: metabolic and hemodynamic responses. Appl. Physiol. Nutr. Metab. 44, 348–356 (2019).

64. Melo, X. et al. Home- vs gym-based exercise delivery modes of two multicomponent intensity training regimes on cardiorespiratory fitness and arterial stiffness in adults with intellectual and developmental disability during the COVID-19 pandemic – a randomized controlled trial. J. Intellect. Disabil. 17446295241242507 (2024) doi:10.1177/17446295241242507.

65. Cox, E. R. et al. Effect of low-volume combined aerobic and resistance high-intensity interval training on vascular health in people with type 2 diabetes: a randomised controlled trial. Eur. J. Appl. Physiol. (2024) doi:10.1007/s00421-024-05473-8.

66. Huang, J. et al. Comparative Effects of Different Exercise Types on Cardiovascular Health and Executive Function in Sedentary Young Individuals. Med. Sci. Sports Exerc. 57, 1110–1122 (2025).

67. Moncion, K. et al. Cardiorespiratory Fitness Benefits of High-Intensity Interval Training After Stroke: A Randomized Controlled Trial. Stroke 55, 2202–2211 (2024).

68. Zarekar, T., Hajifathali, A. & Ahmadizad, S. The effect of High Intensity Interval Exercise on Platelet Engraftment in Autologous Bone Marrow Transplantation (BMT). Int. J. Hematol.-Oncol. Stem Cell Res. (2024) doi:10.18502/ijhoscr.v18i3.16104..

**Appendix 7.** Forest plots for hemostasis and vascular stiffness indicators.


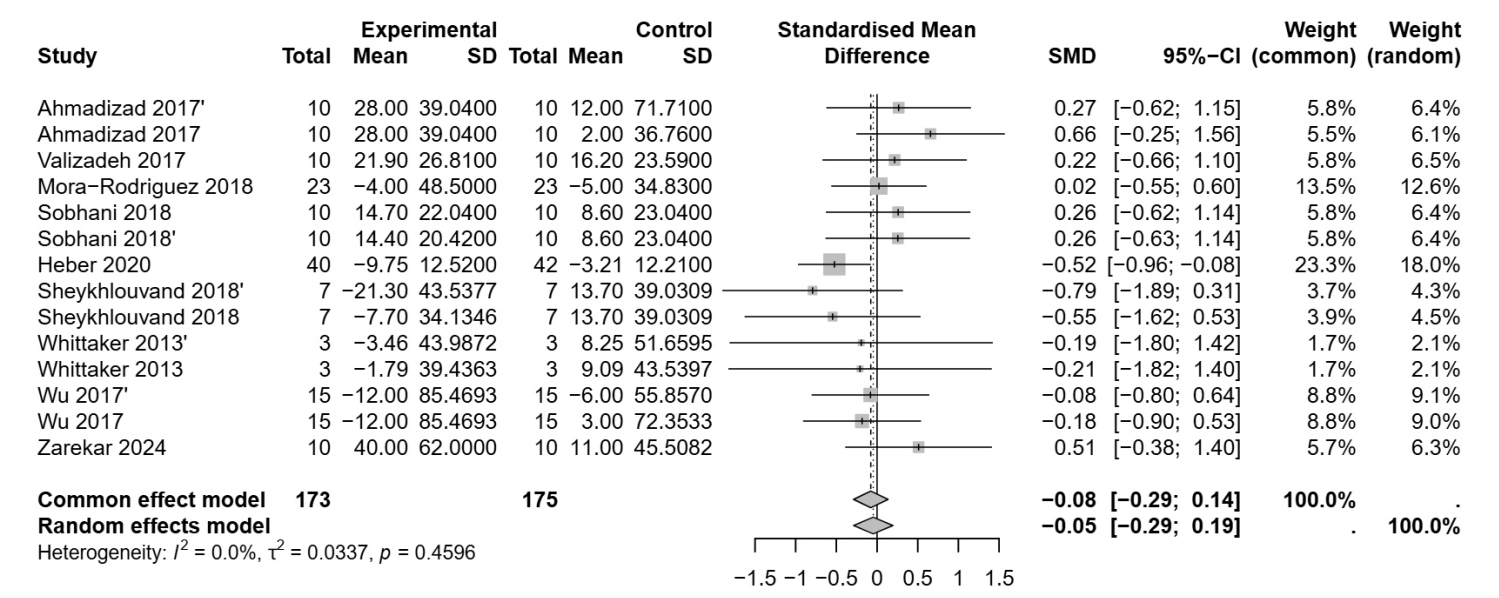


**Appendix 7A** forest plot of PLT.


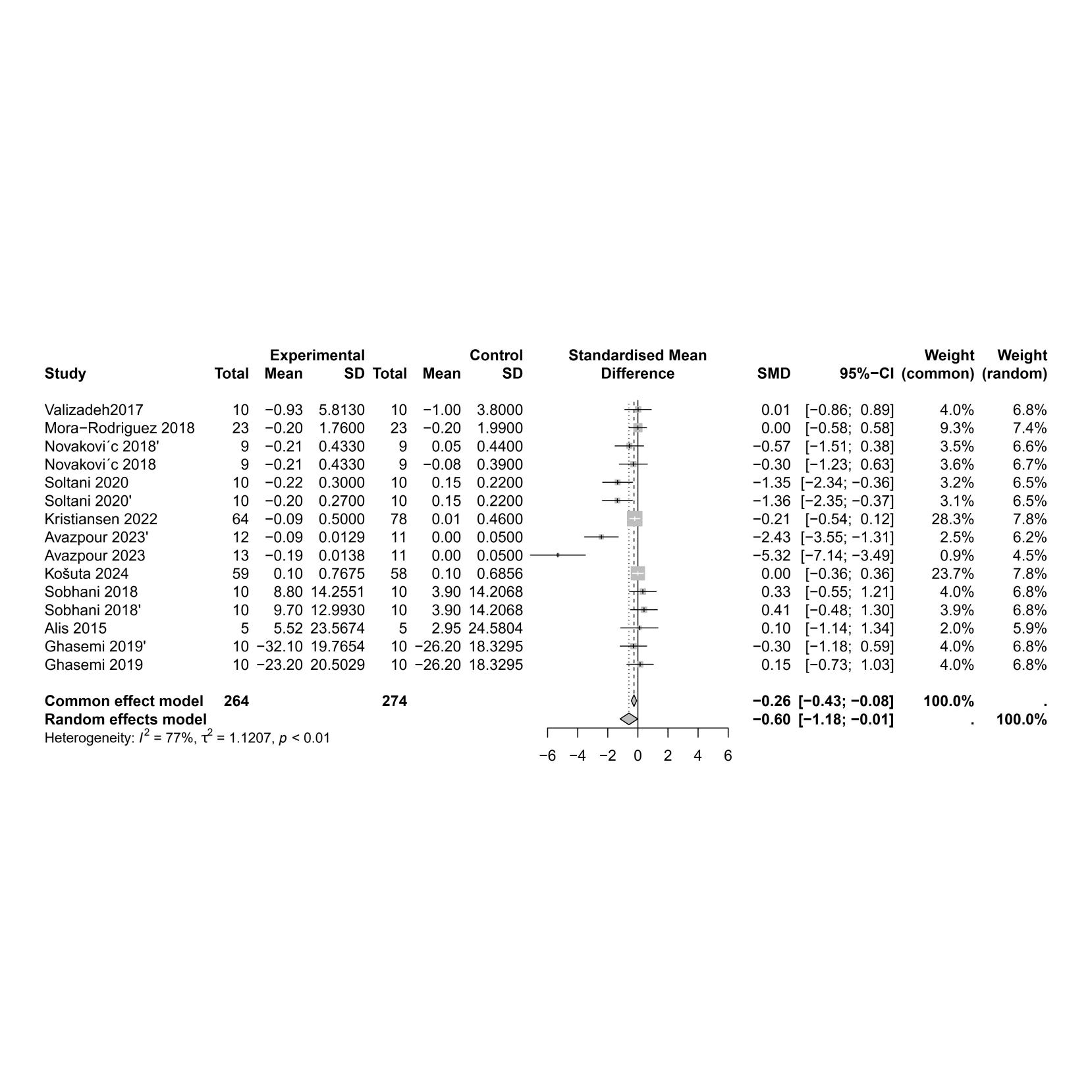


**Appendix 7B** forest plot of FIB.


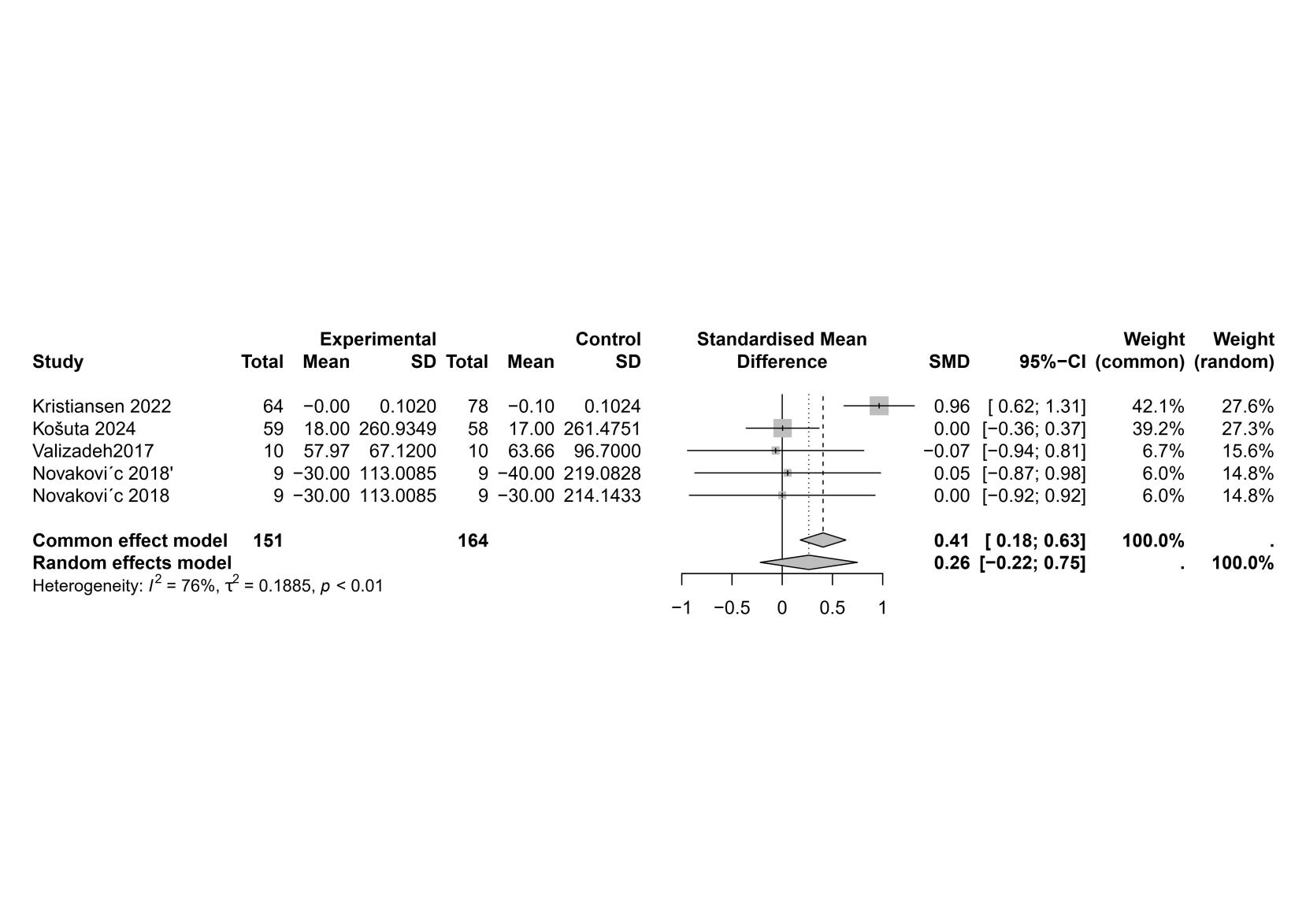


**Appendix 7C** forest plot of D-D.


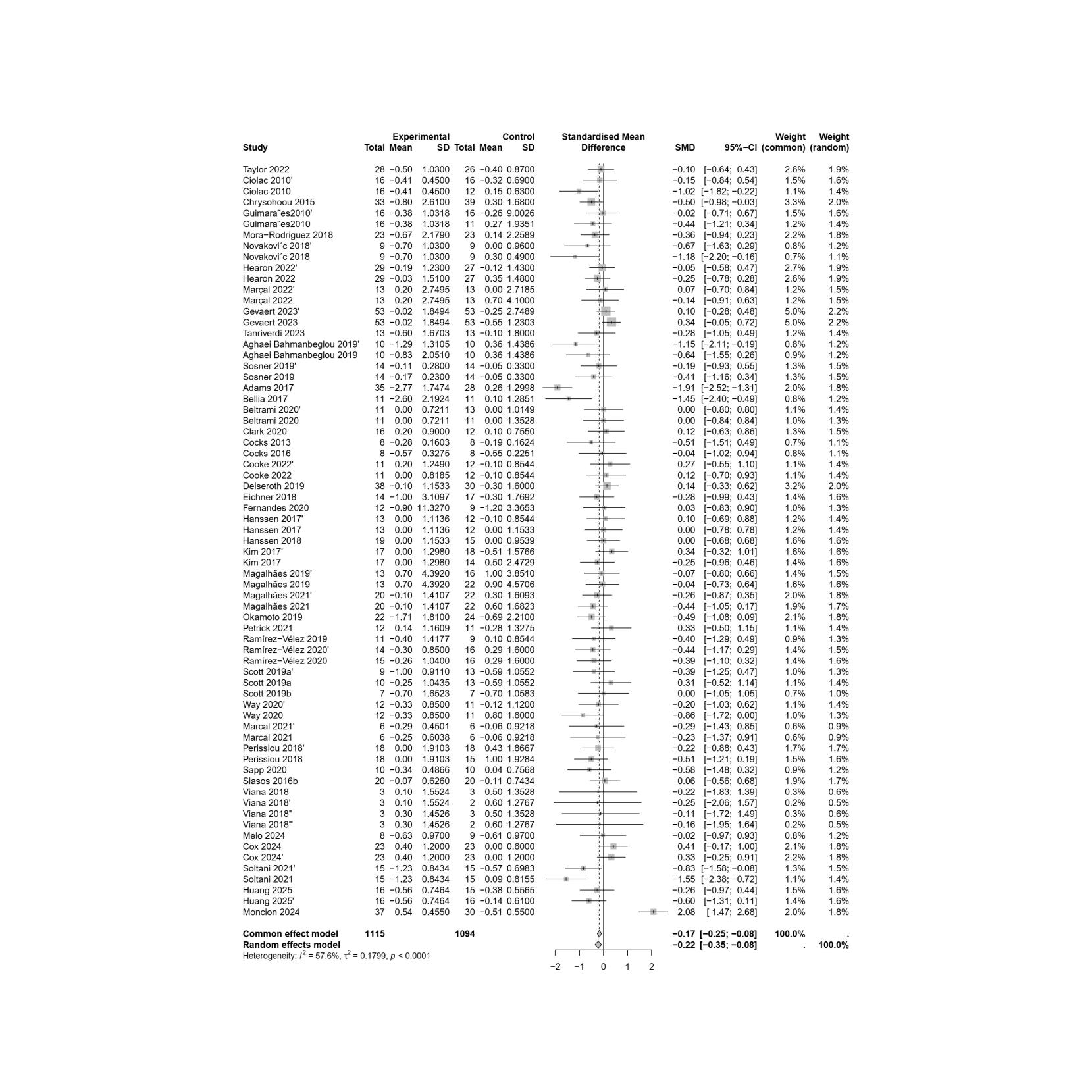


**Appendix 7D** forest plot of cfPWV.


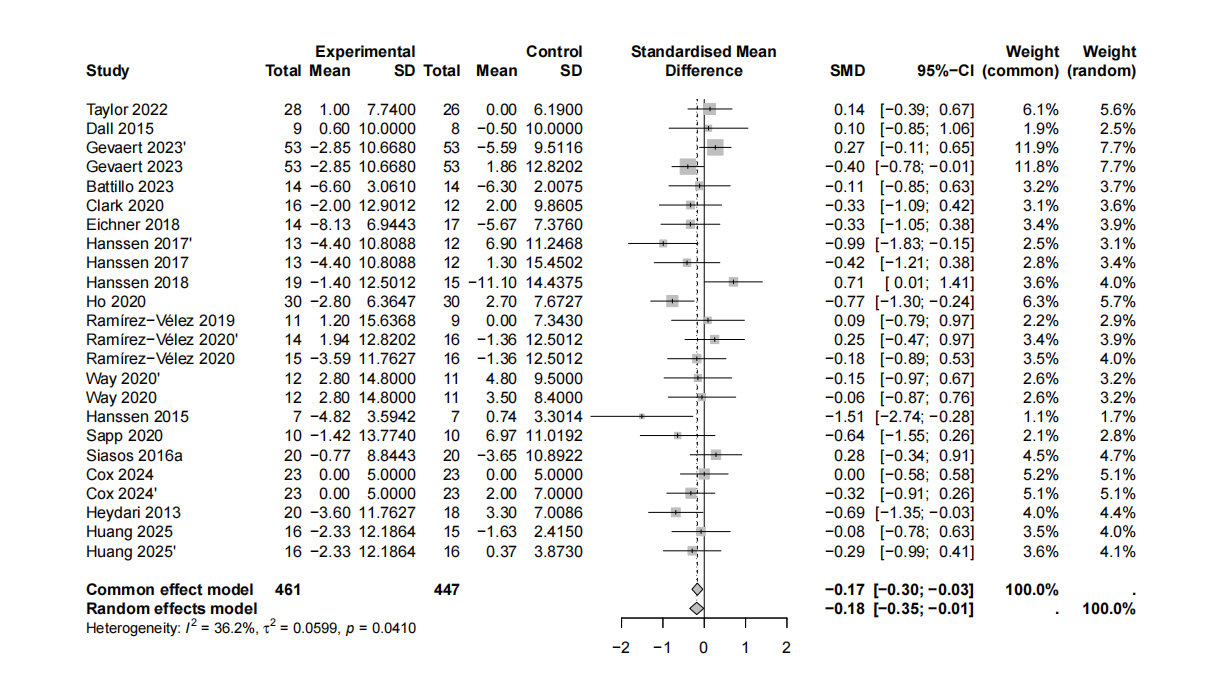


**Appendix 7E** forest plot of AIx.


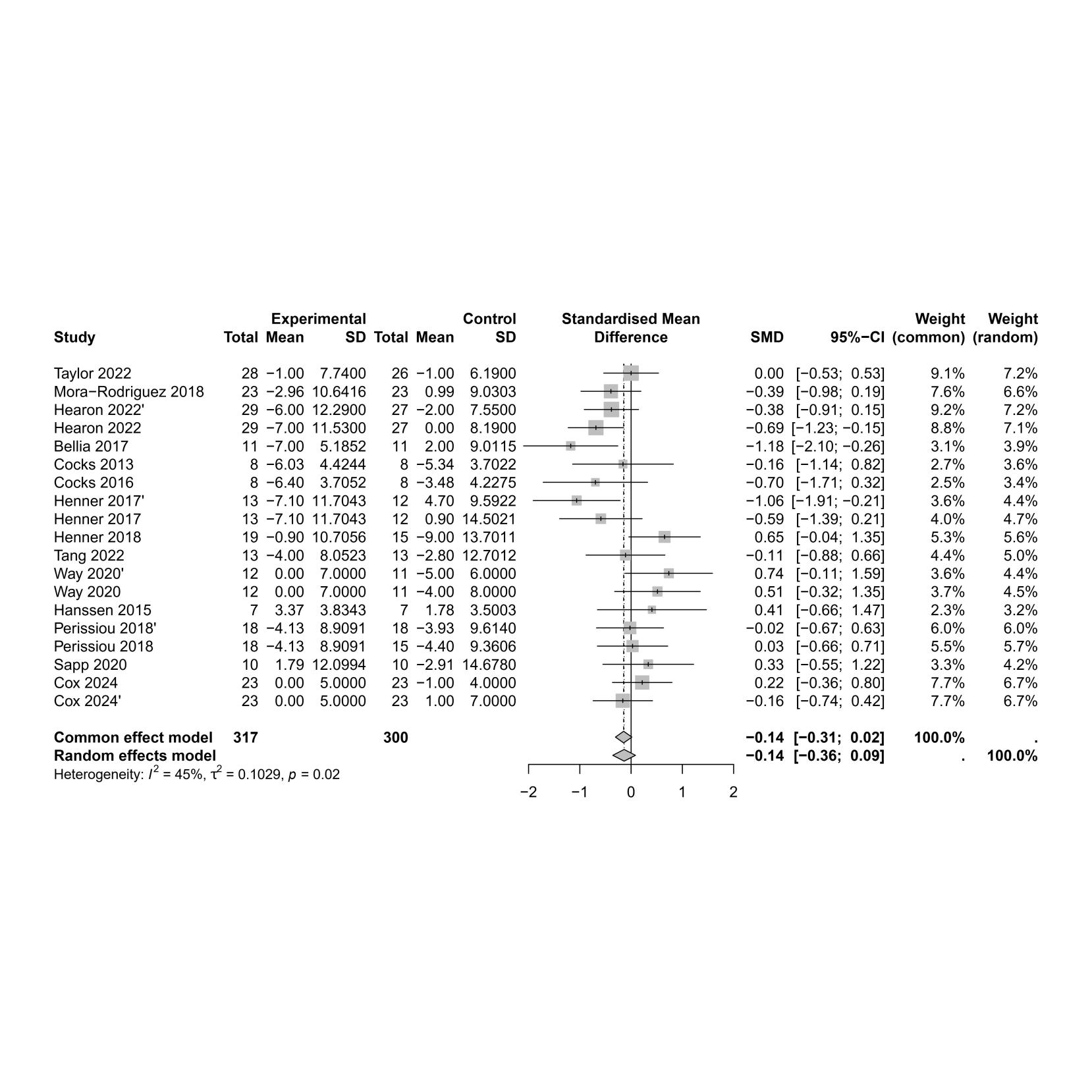


**Appendix 7F** forest plot of AIx@75HR.


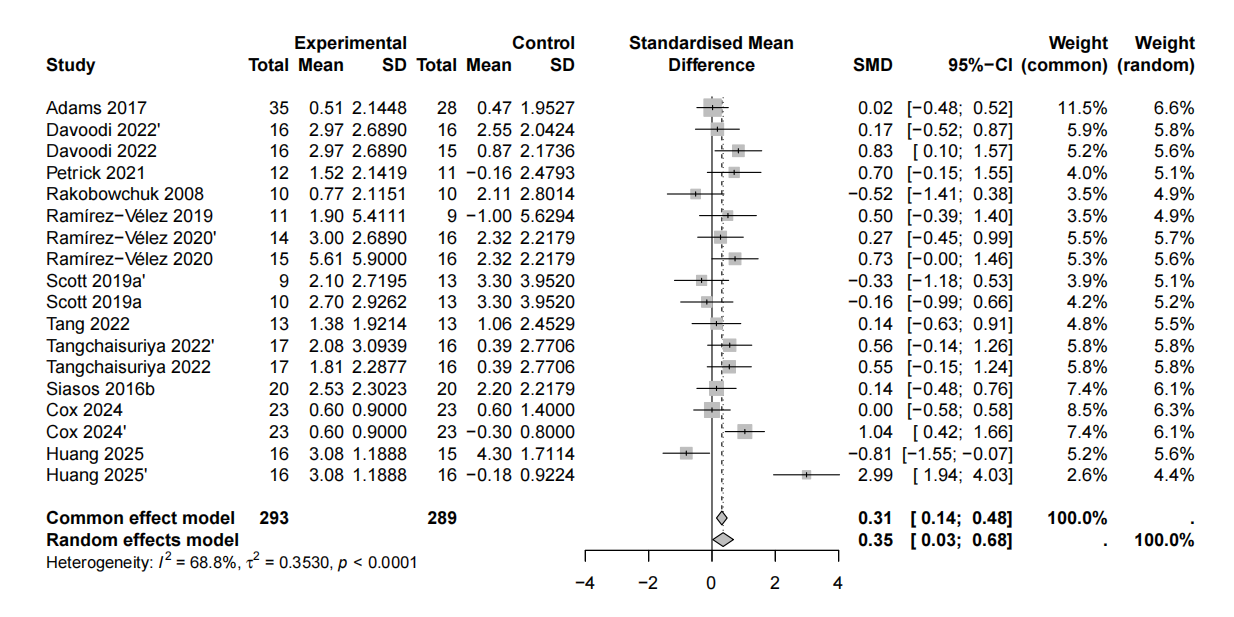


**Appendix 7G** forest plot of FMD.


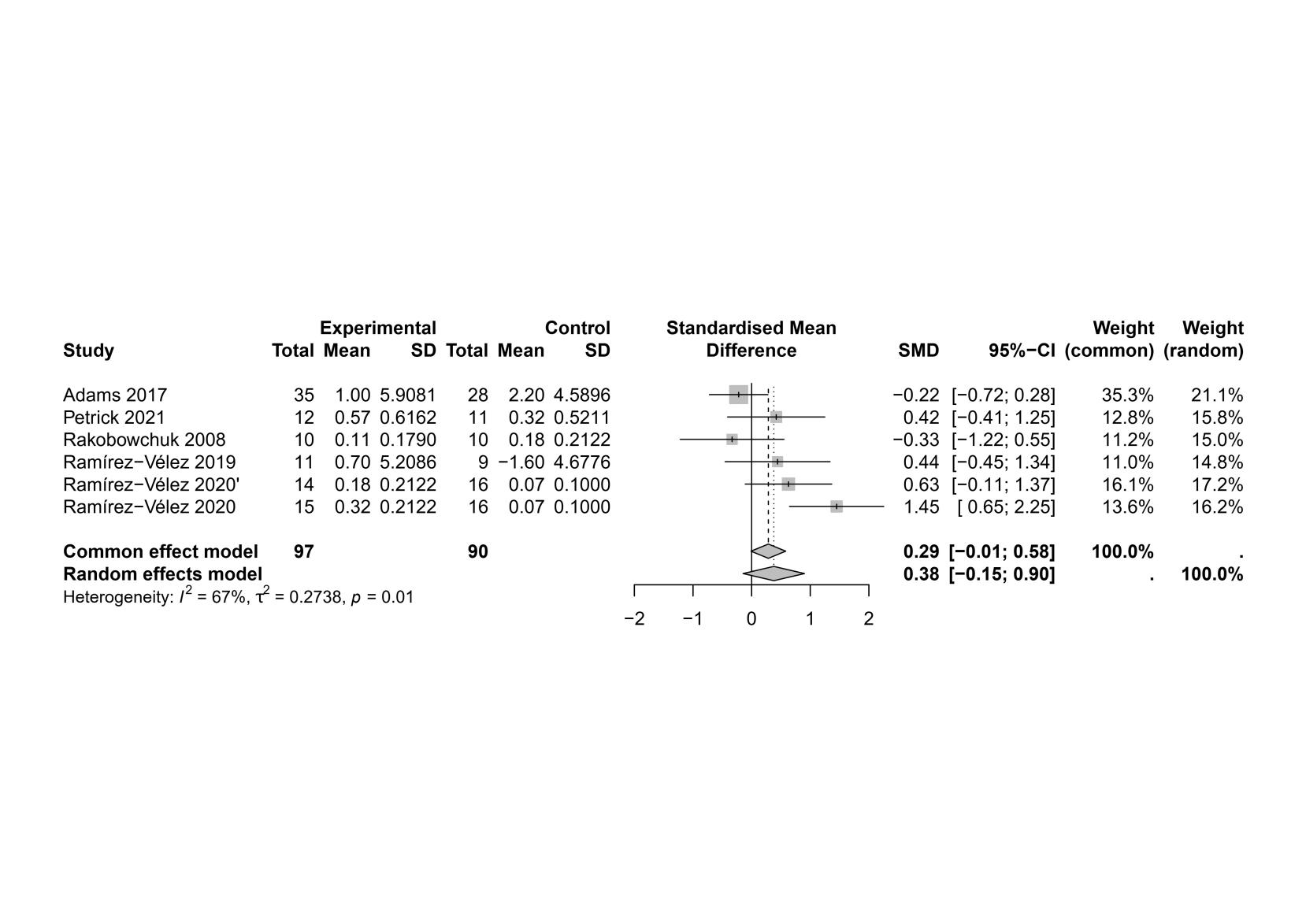


**Appendix 7H** forest plot of nFMD.

**Appendix 8.** Forest plots for subgroup analyses of hemostasis and vascular stiffness indicators.


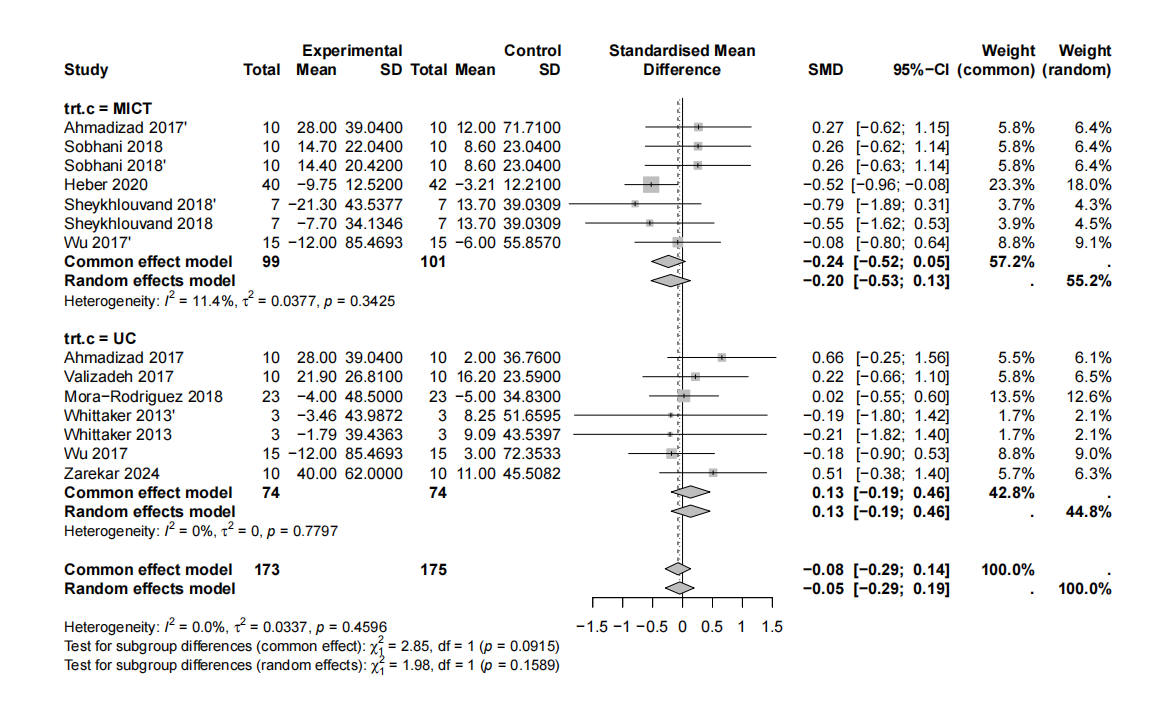


**Appendix 8A** Subgroup analysis of the effect of HIIT on PLT comparing with MICT or UC.


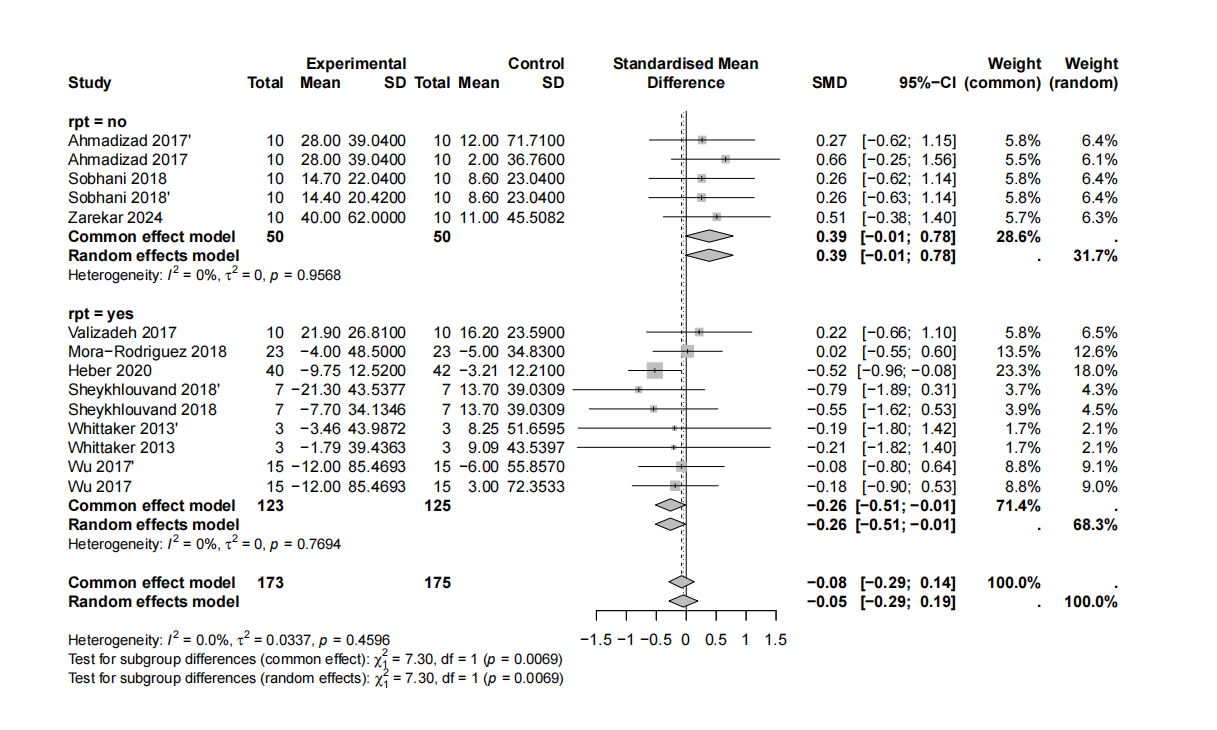


**Appendix 8B** Subgroup analysis of the effect of repeated/single HIIT on PLT.


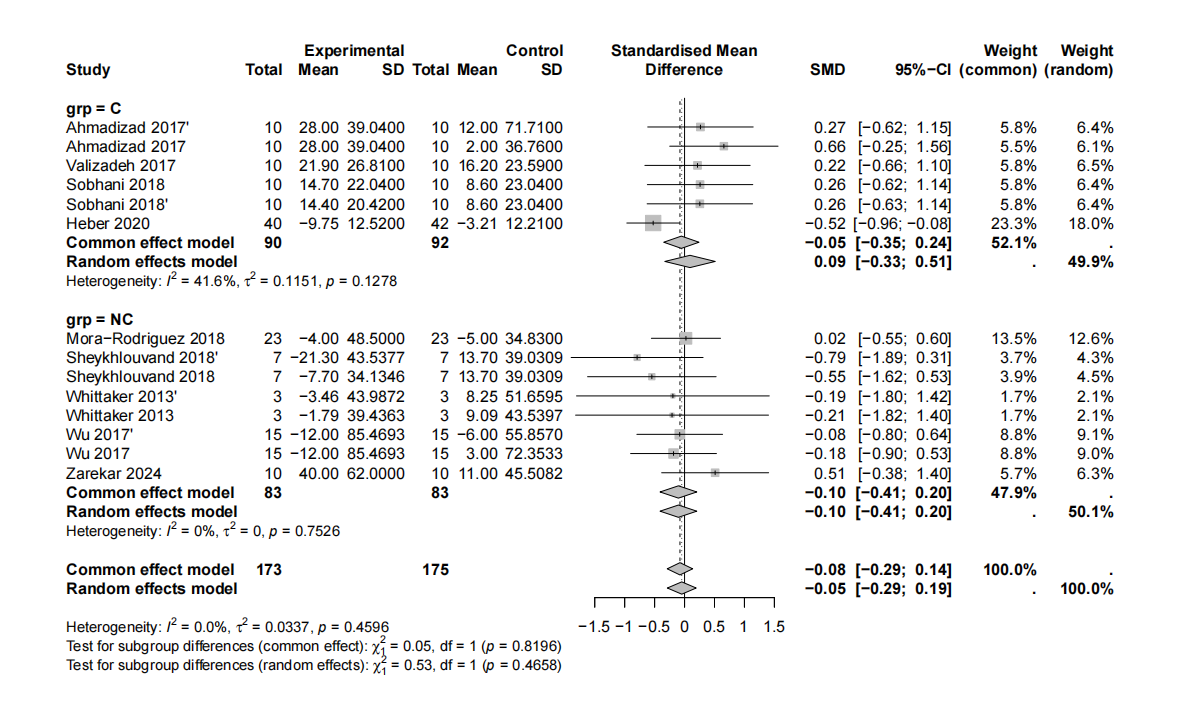


**Appendix 8C** Subgroup analysis of the effect of HIIT on PLT in patients with/without CVD.


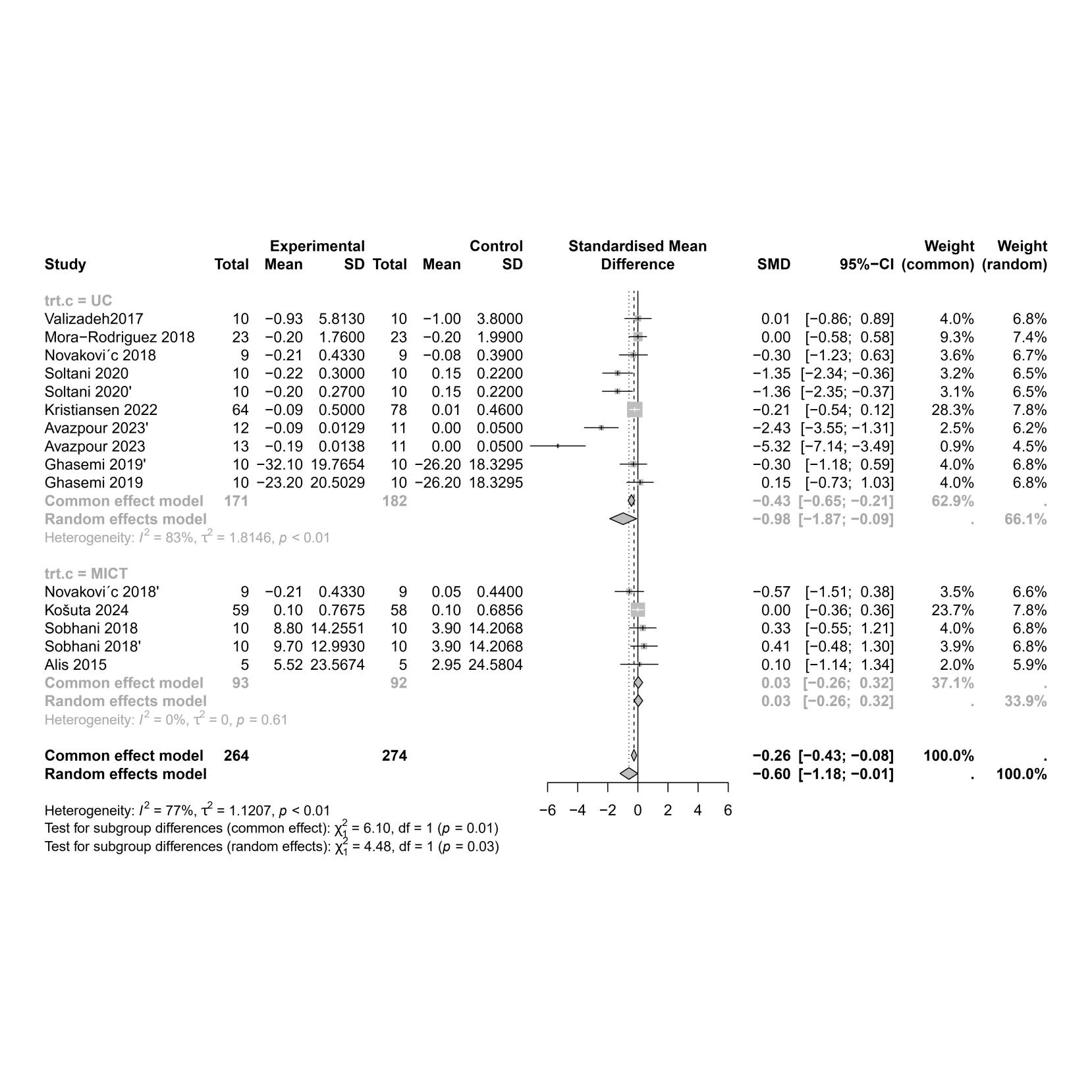


**Appendix 8D** Subgroup analysis of the effect of HIIT on FIB comparing with MICT or UC.


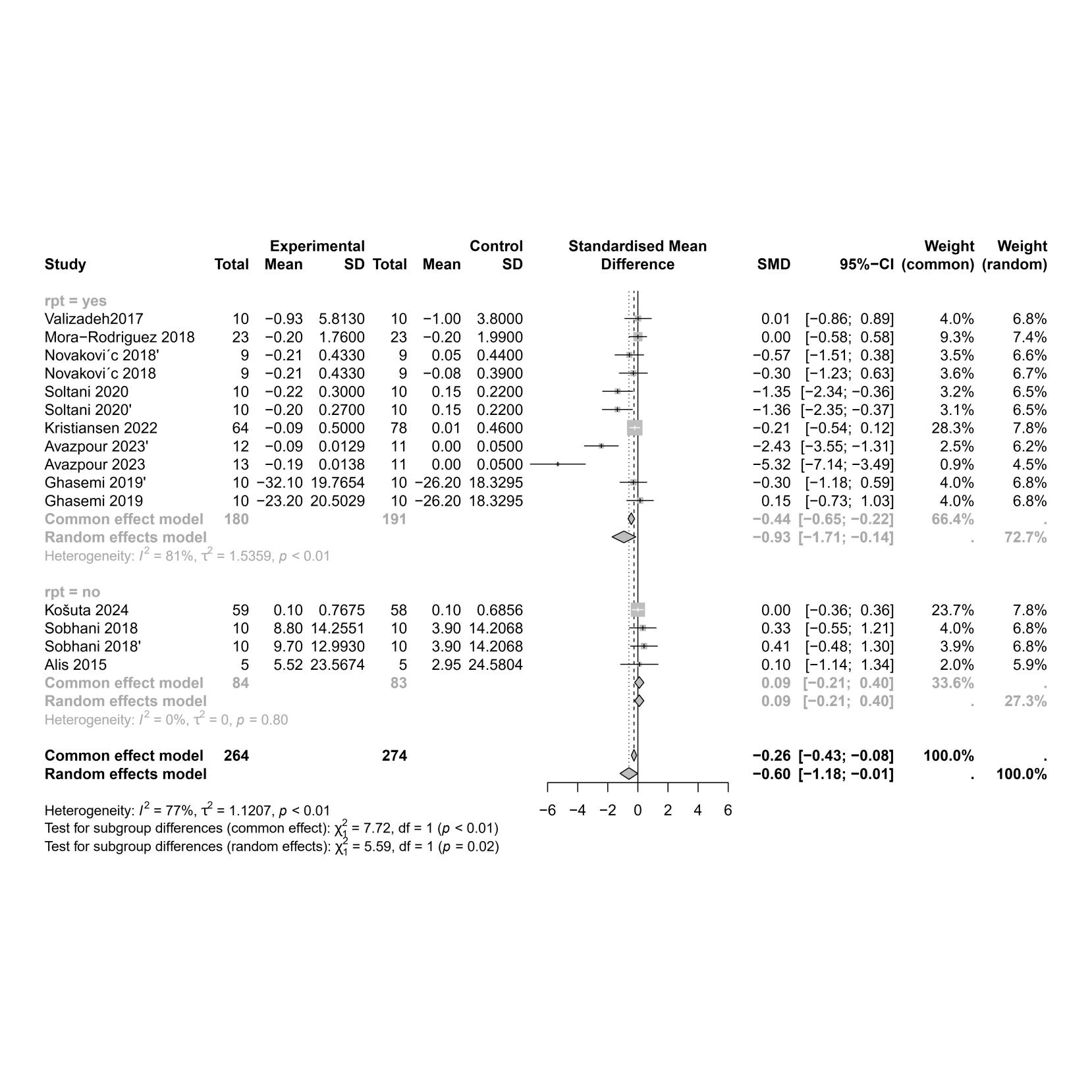


**Appendix 8E** Subgroup analysis of the effect of repeated/single HIIT on FIB.


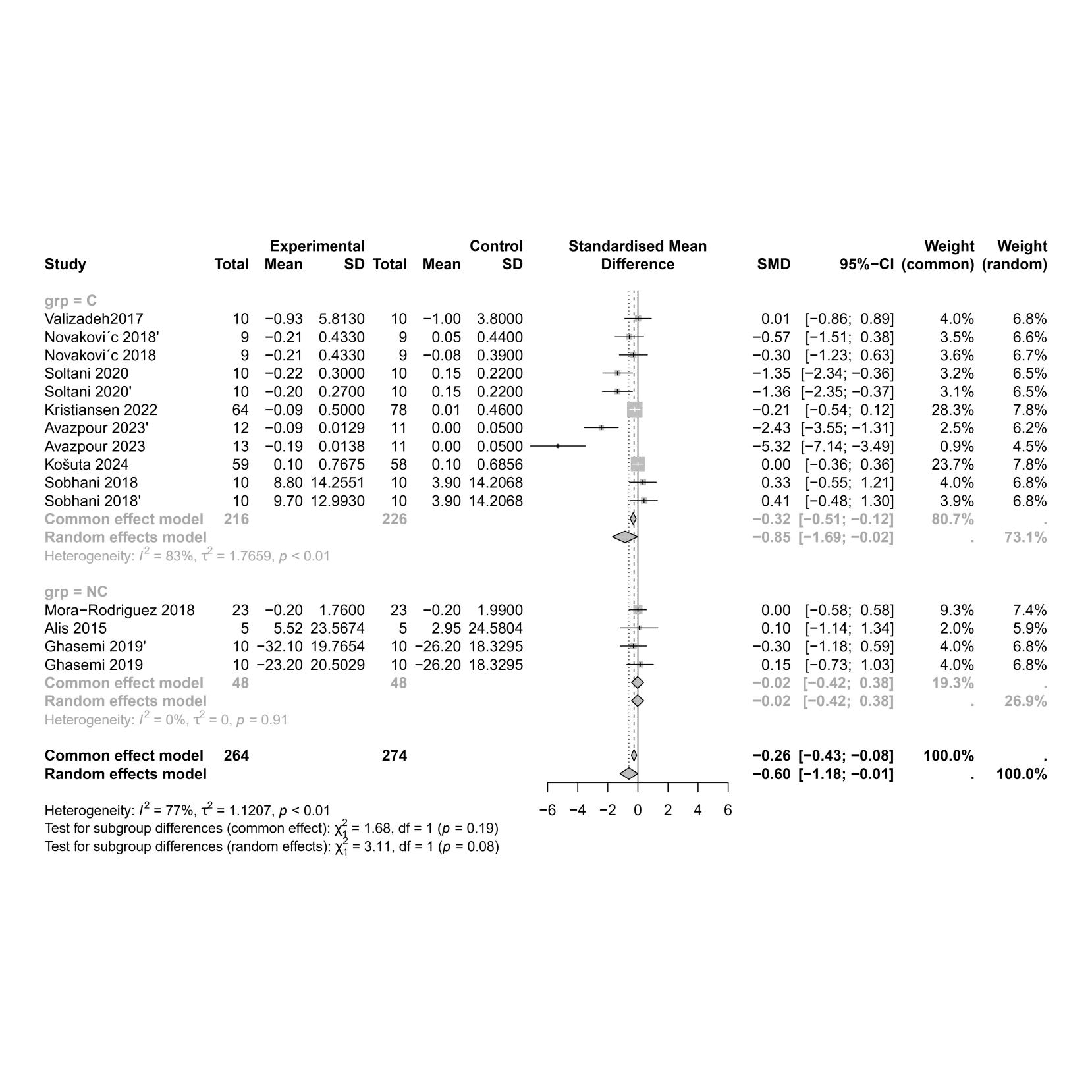


**Appendix 8F** Subgroup analysis of the effect of HIIT on FIB in patients with/without CVD.


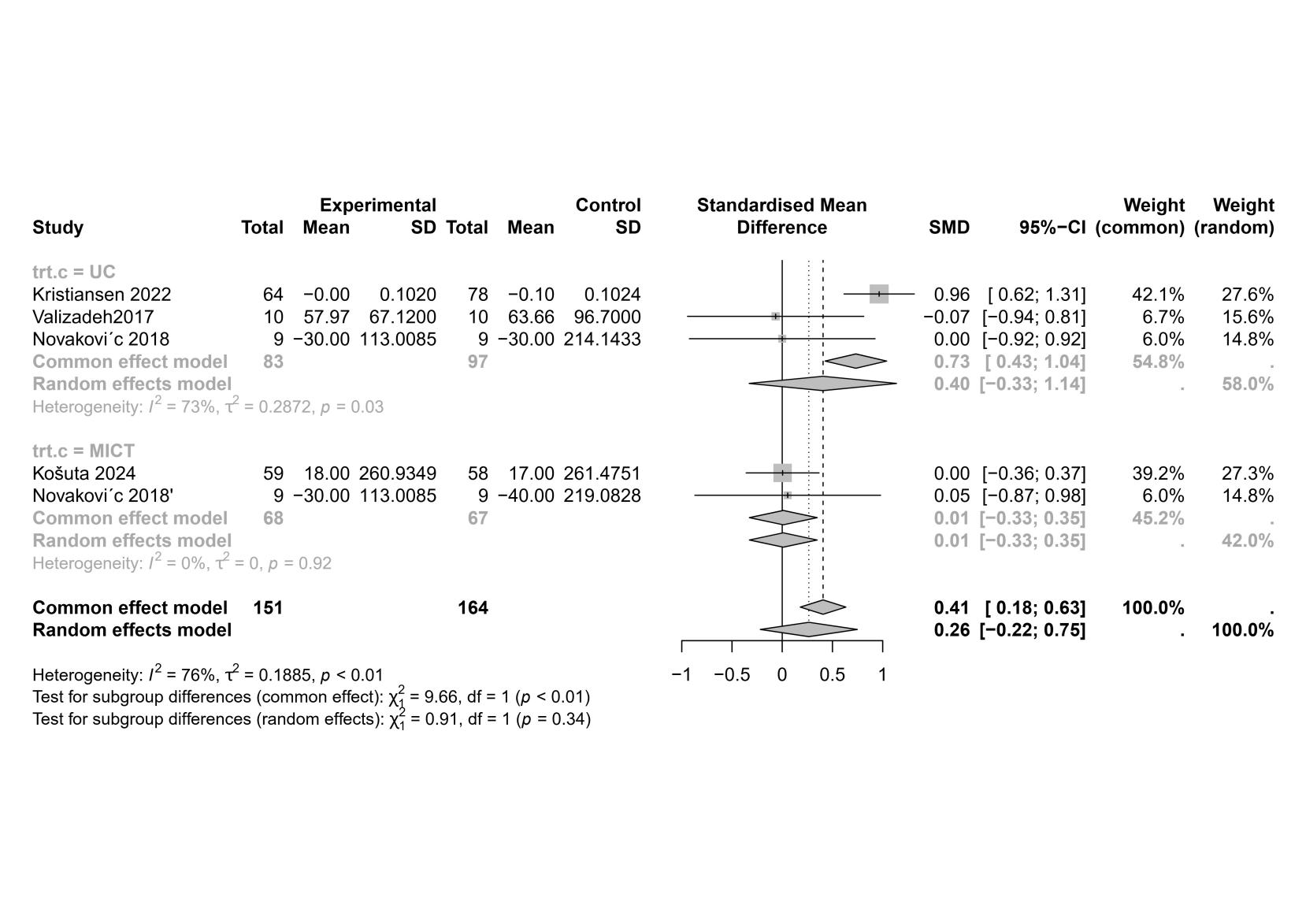


**Appendix 8G** Subgroup analysis of the effect of HIIT on D-D comparing with MICT or UC.


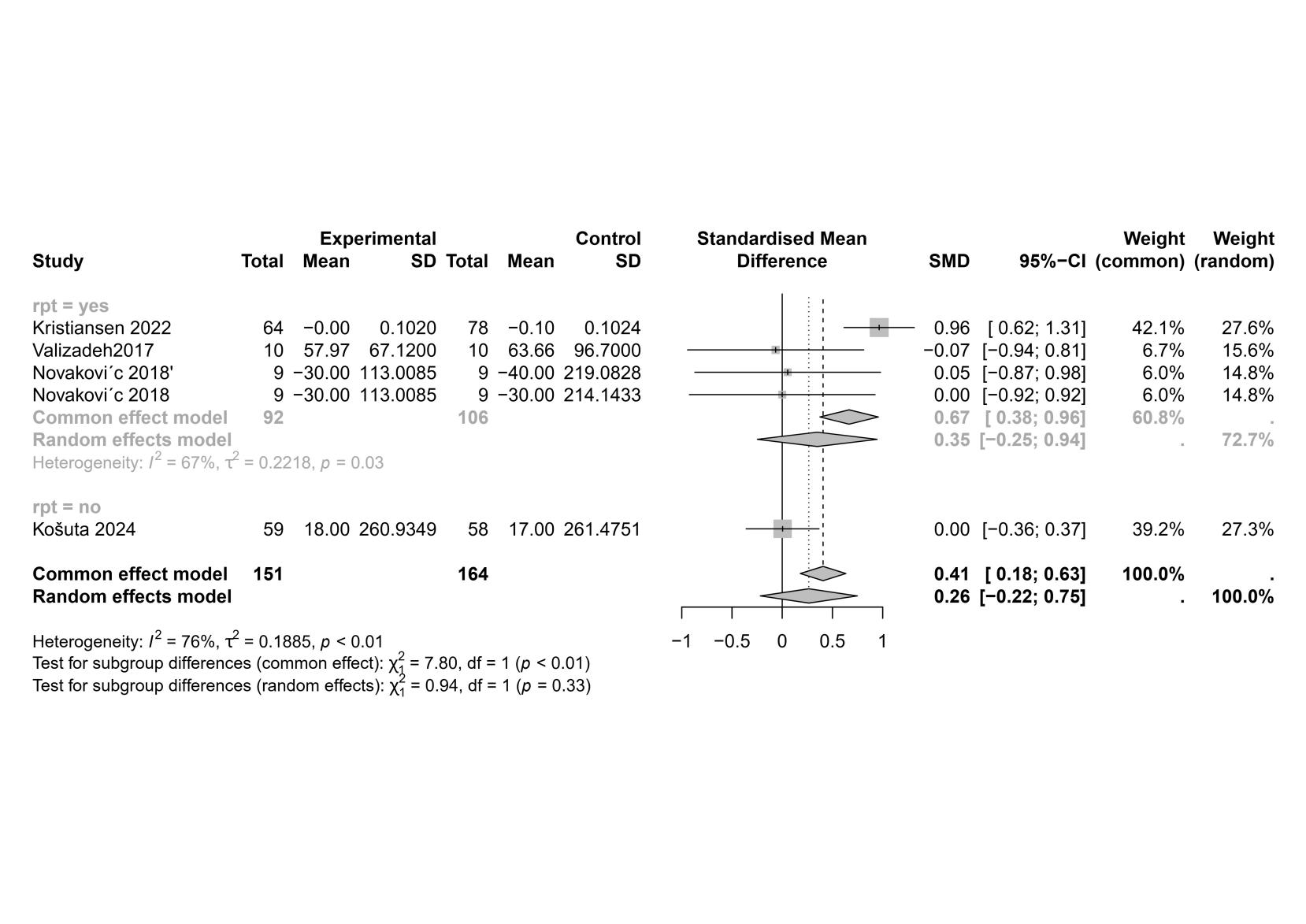


**Appendix 8H** Subgroup analysis of the effect of repeated/single HIIT on D-D.


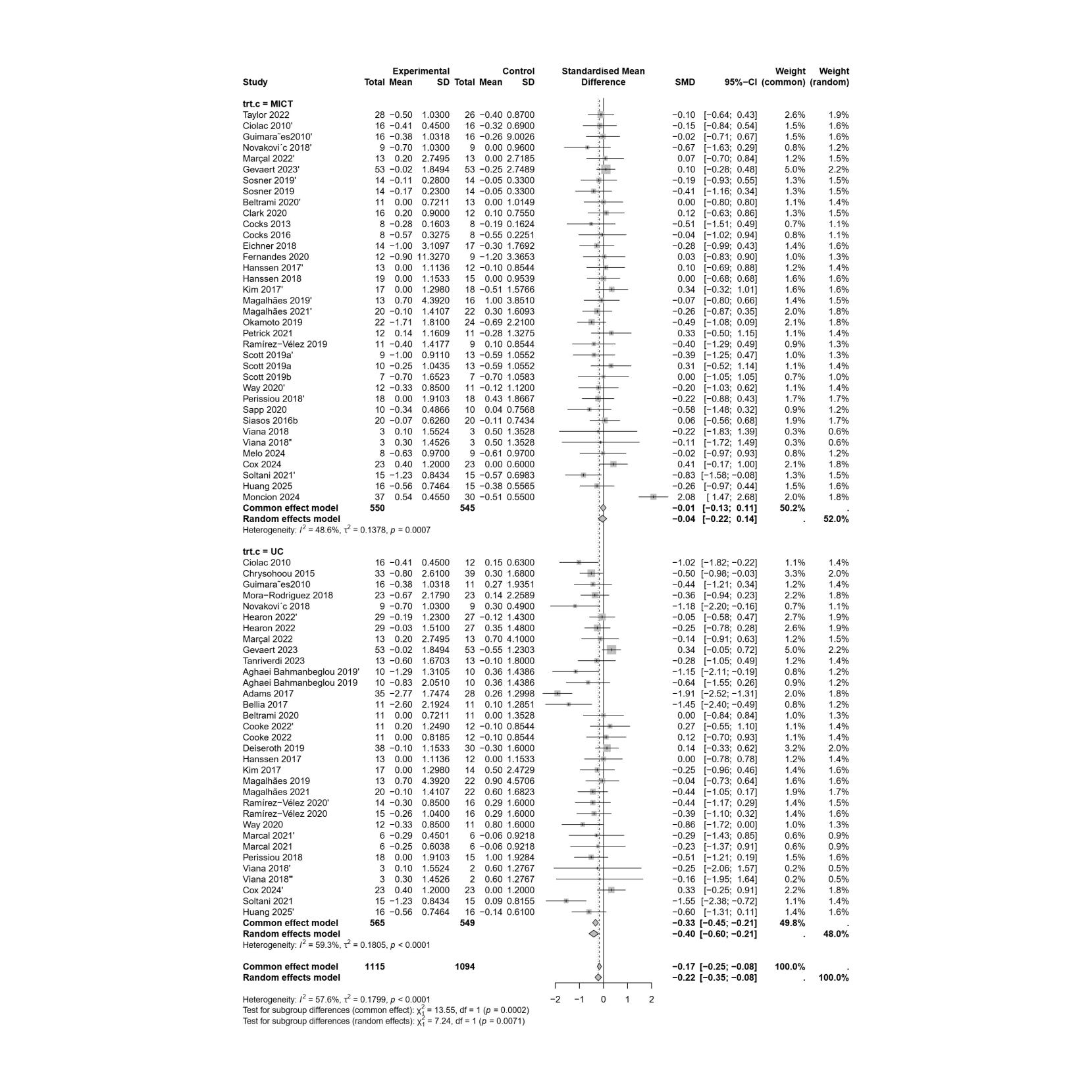


**Appendix 8I** Subgroup analysis of the effect of HIIT on cfPWV comparing with MICT or UC.


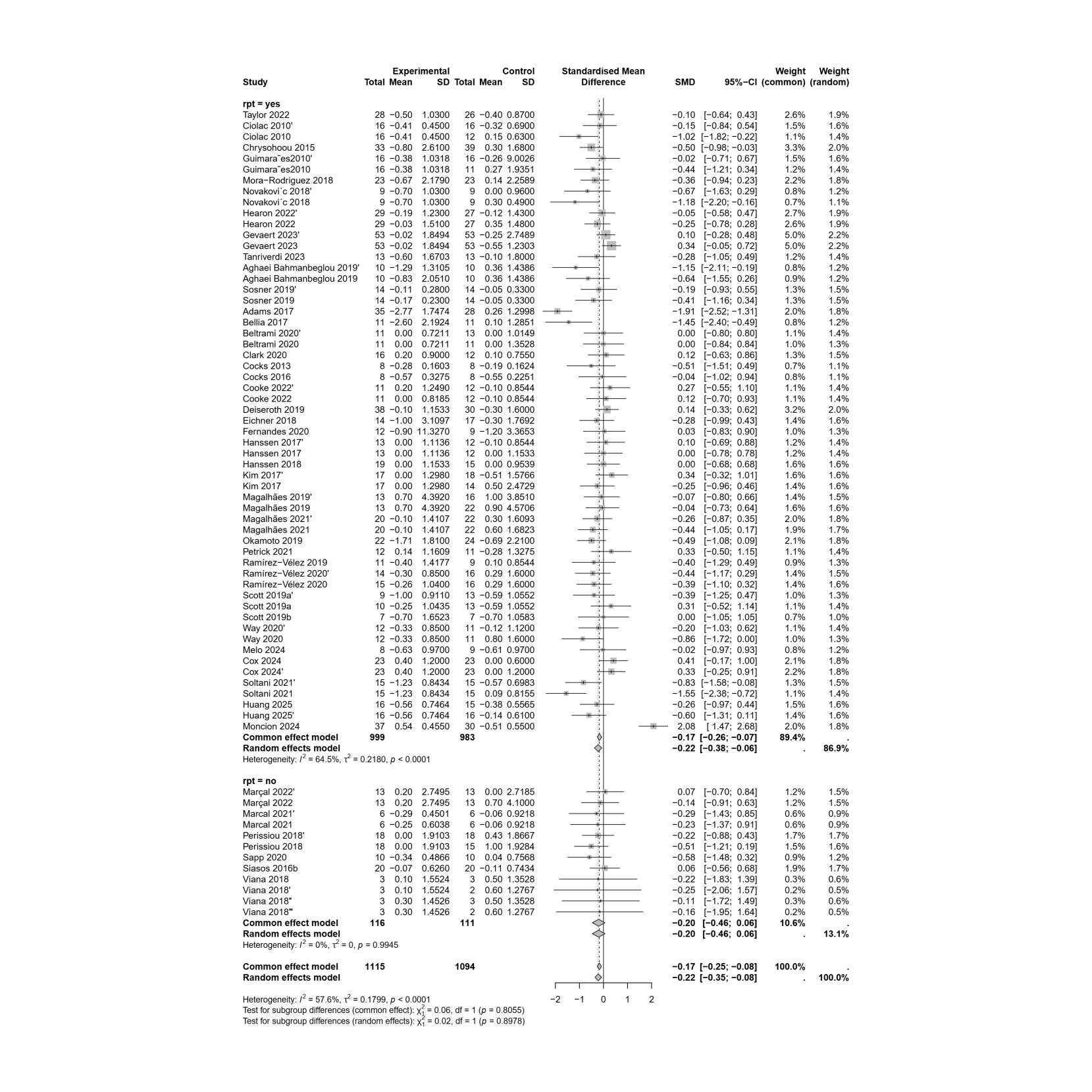


**Appendix 8J** Subgroup analysis of the effect of repeated/single HIIT on cfPWV.


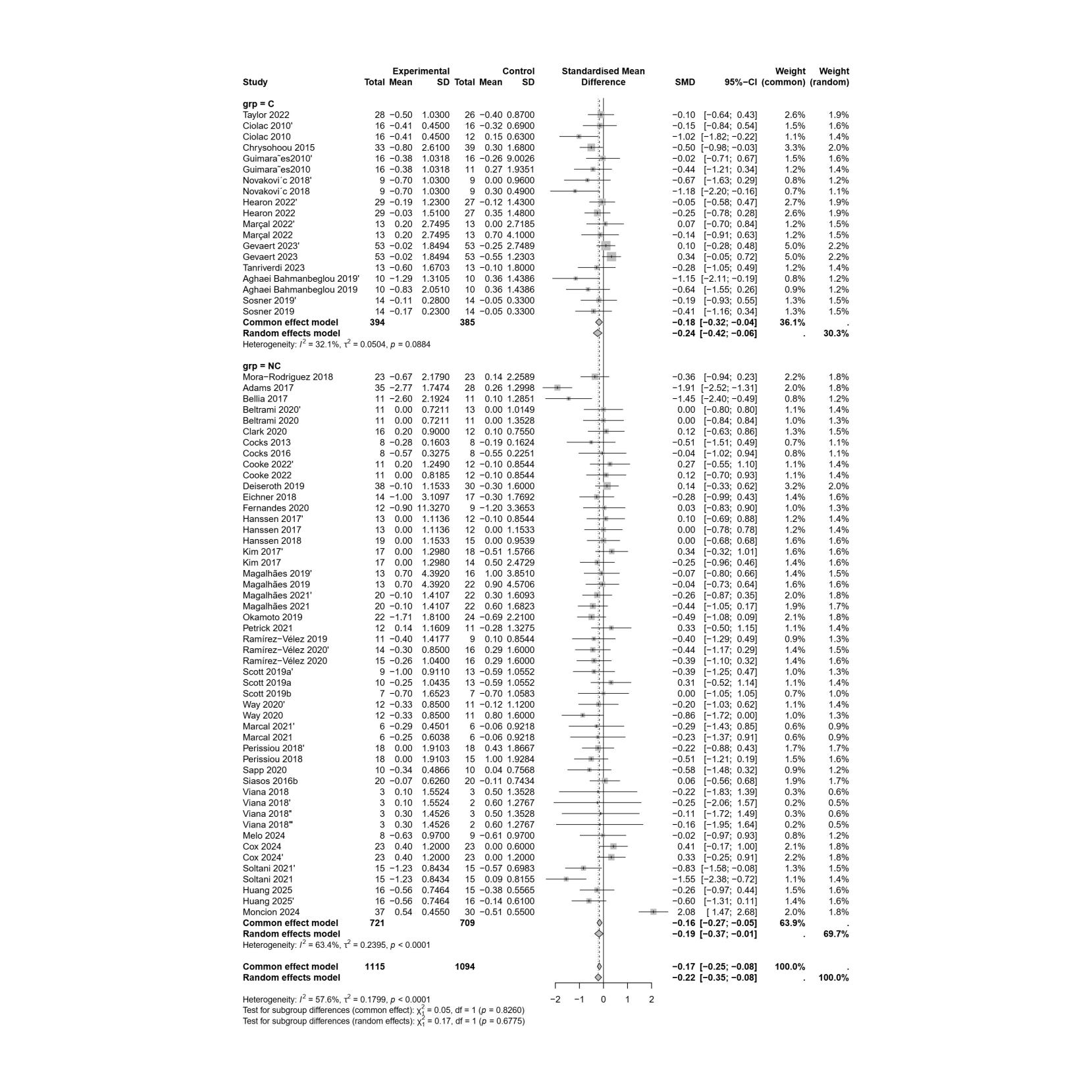


**Appendix 8K** Subgroup analysis of the effect of HIIT on cfPWV in patients with/without CVD.


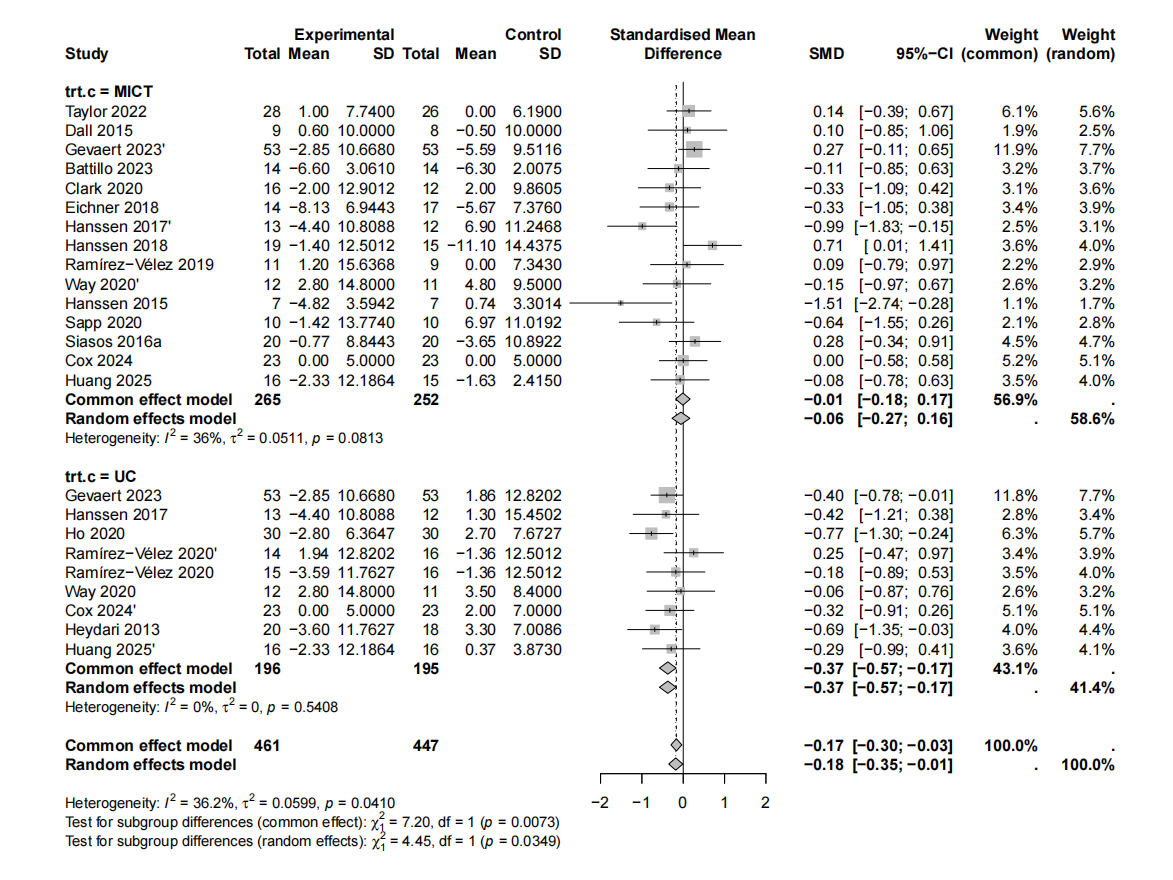


**Appendix 8L** Subgroup analysis of the effect of HIIT on AIx comparing with MICT or UC.


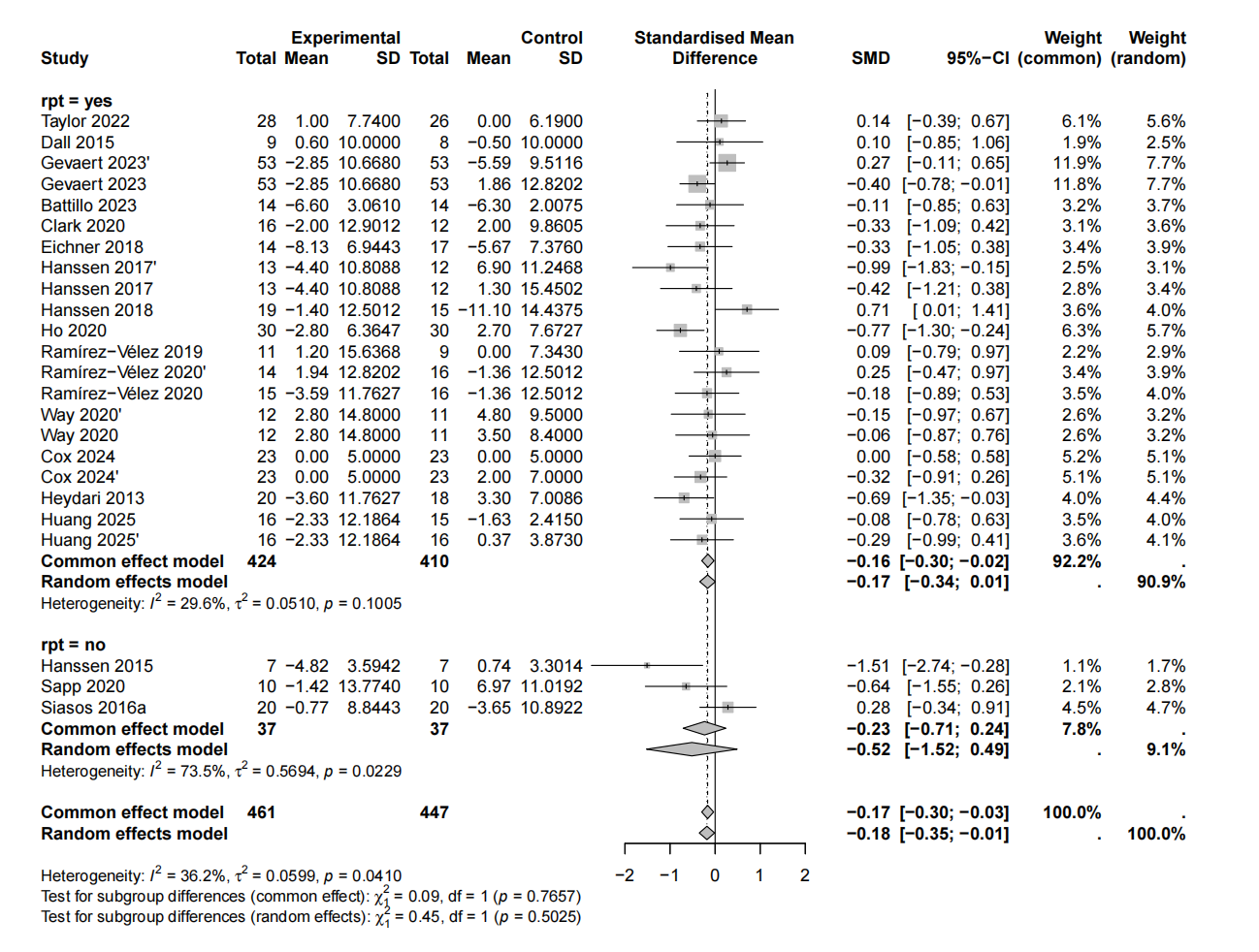


**Appendix 8M** Subgroup analysis of the effect of repeated/single HIIT on AIx.


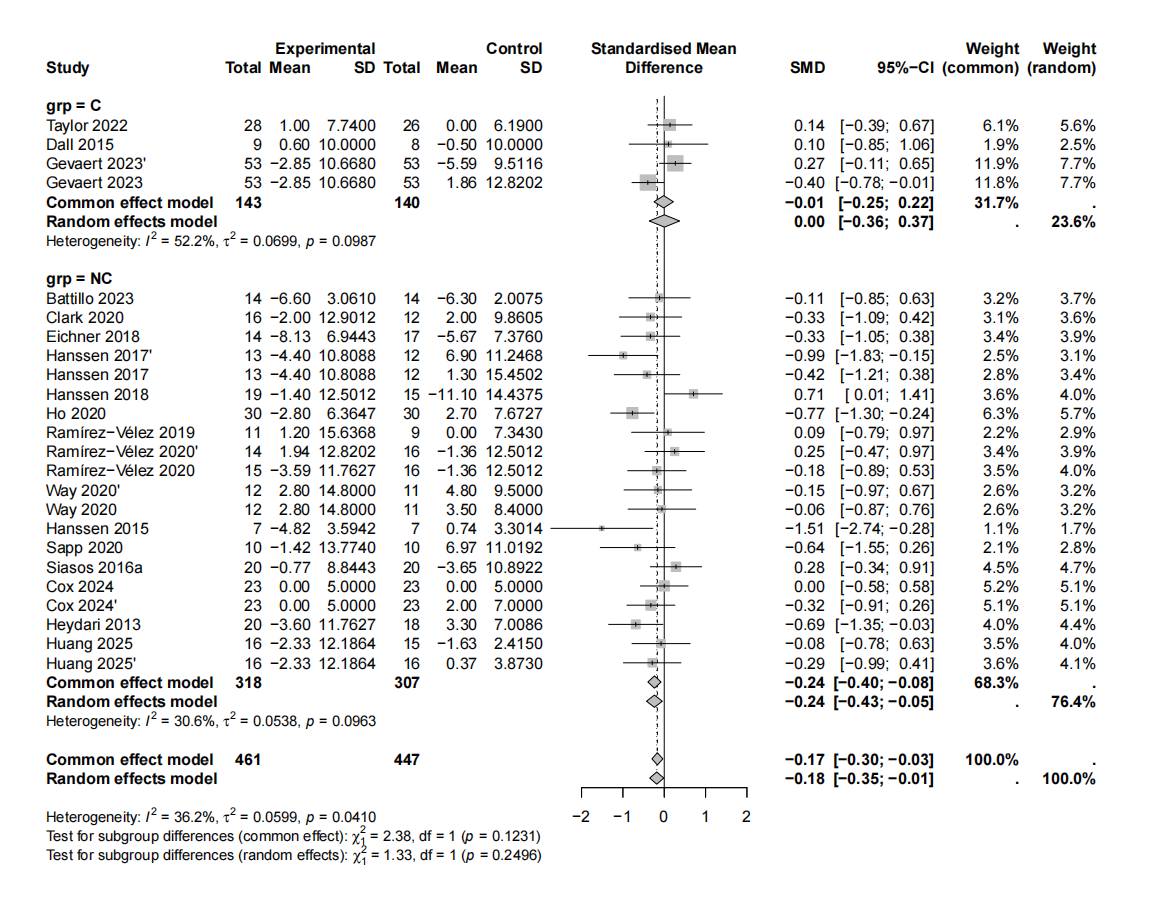


**Appendix 8N** Subgroup analysis of the effect of HIIT on AIx in patients with/without CVD.


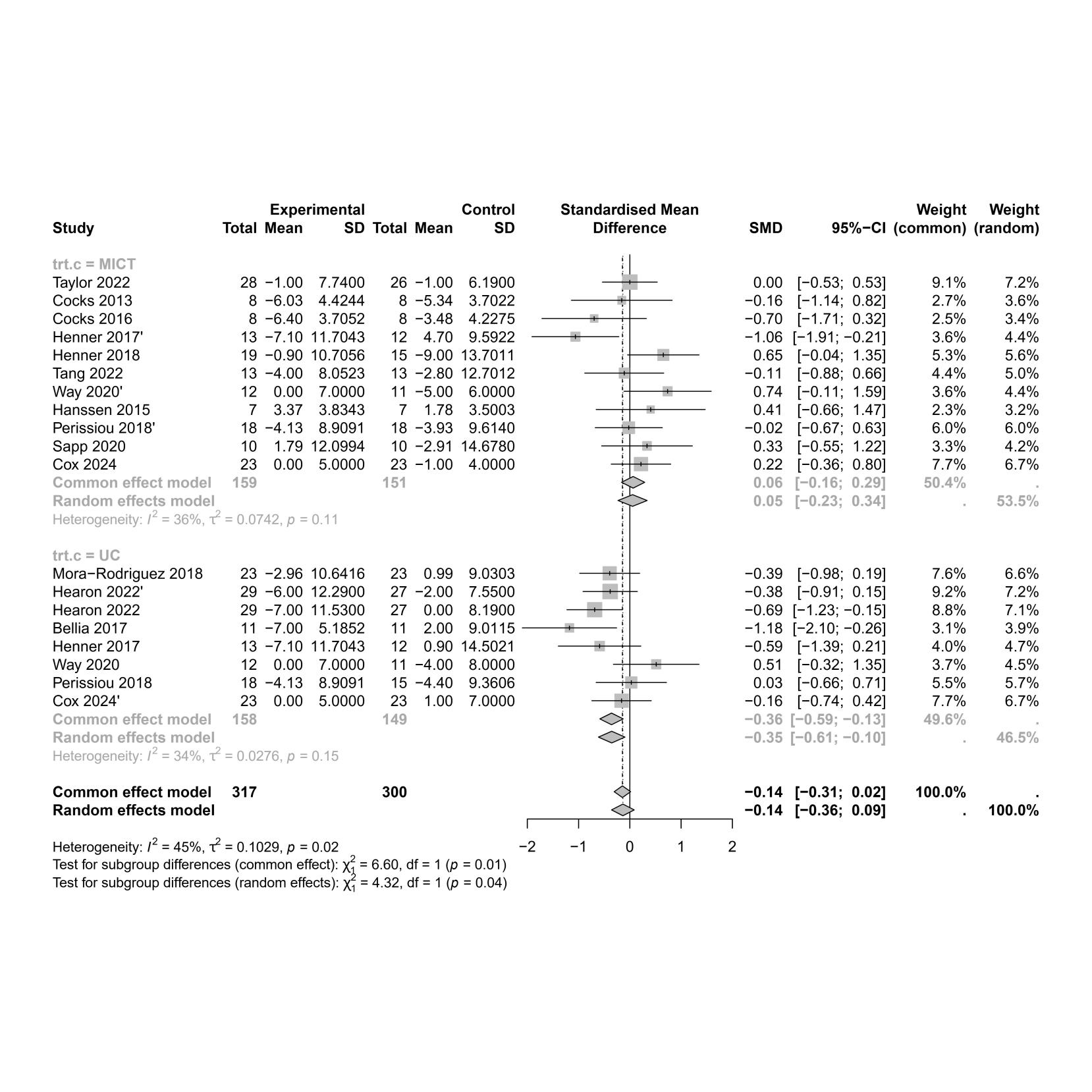


**Appendix 8O** Subgroup analysis of the effect of HIIT on AIx@75HR comparing with MICT or UC.


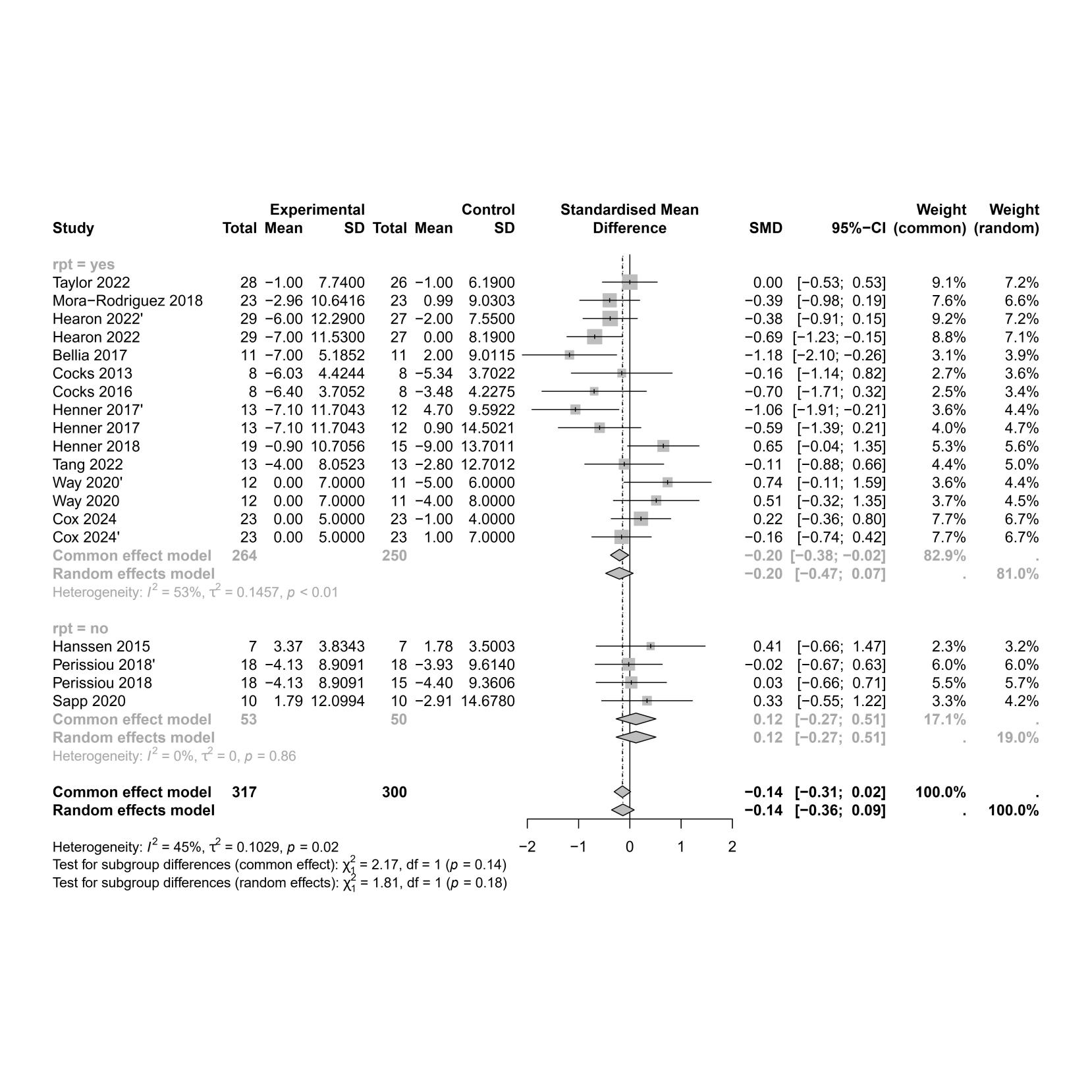


**Appendix 8P** Subgroup analysis of the effect of repeated/single HIIT on AIx@75HR.


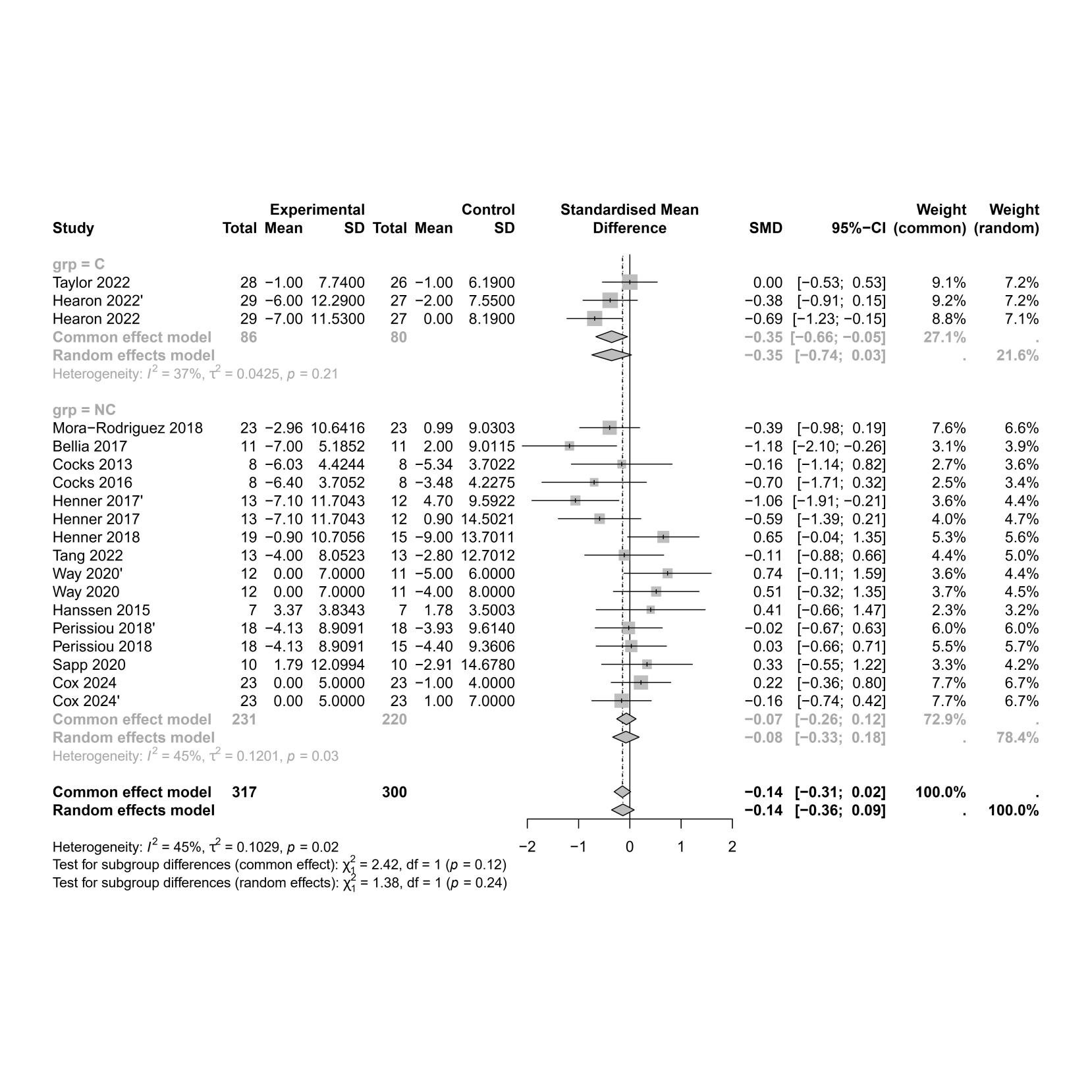


**Appendix 8Q** Subgroup analysis of the effect of HIIT on AIx@75HR in patients with/without CVD.


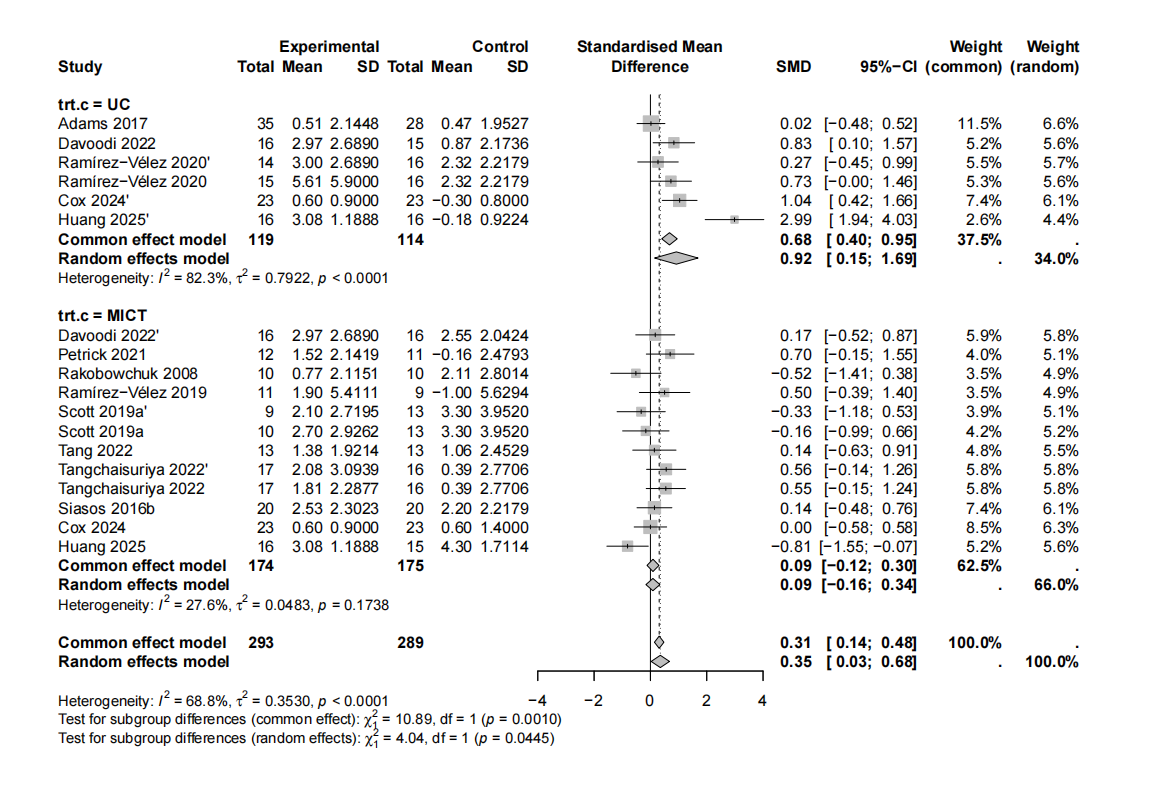


**Appendix 8R** Subgroup analysis of the effect of HIIT on FMD comparing with MICT or UC.


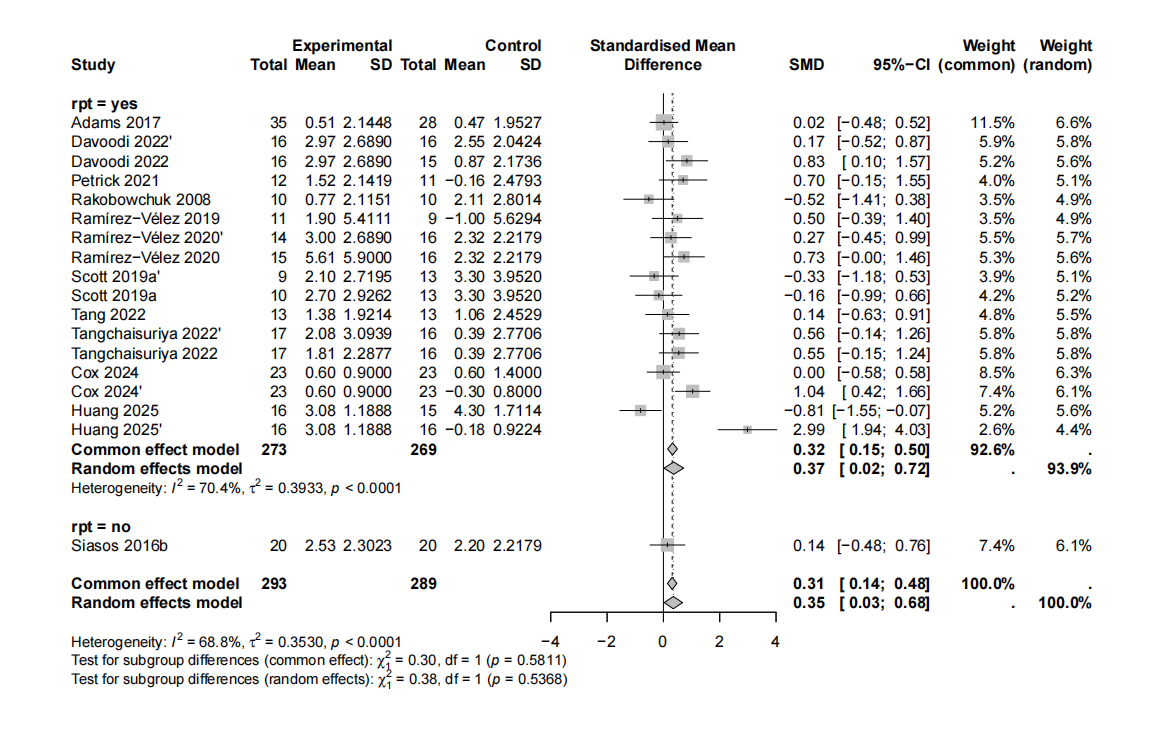


**Appendix 8S** Subgroup analysis of the effect of repeated/single HIIT on FMD.


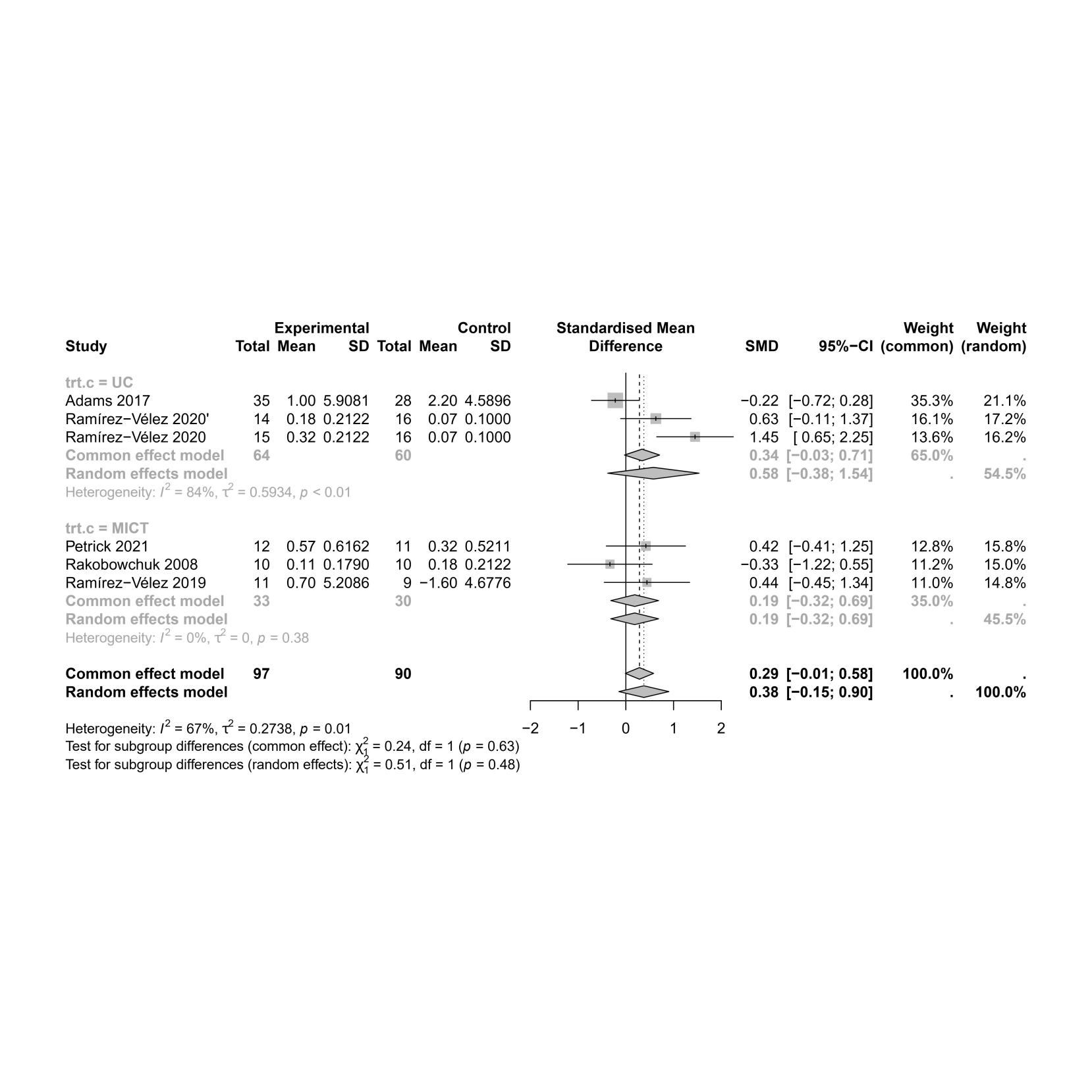


**Appendix 8T** Subgroup analysis of the effect of HIIT on nFMD comparing with MICT or UC.


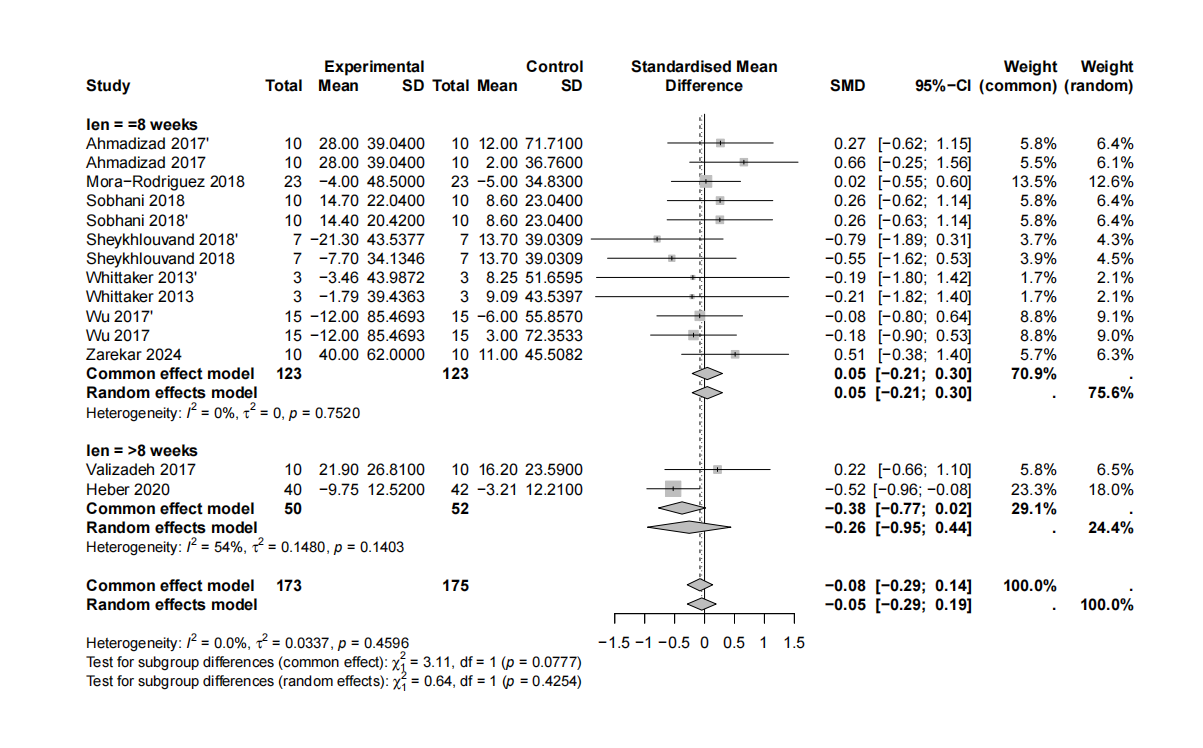


**Appendix 8-1** Subgroup Analysis of ≤8 weeks vs. >8 weeks HIIT on PLT.


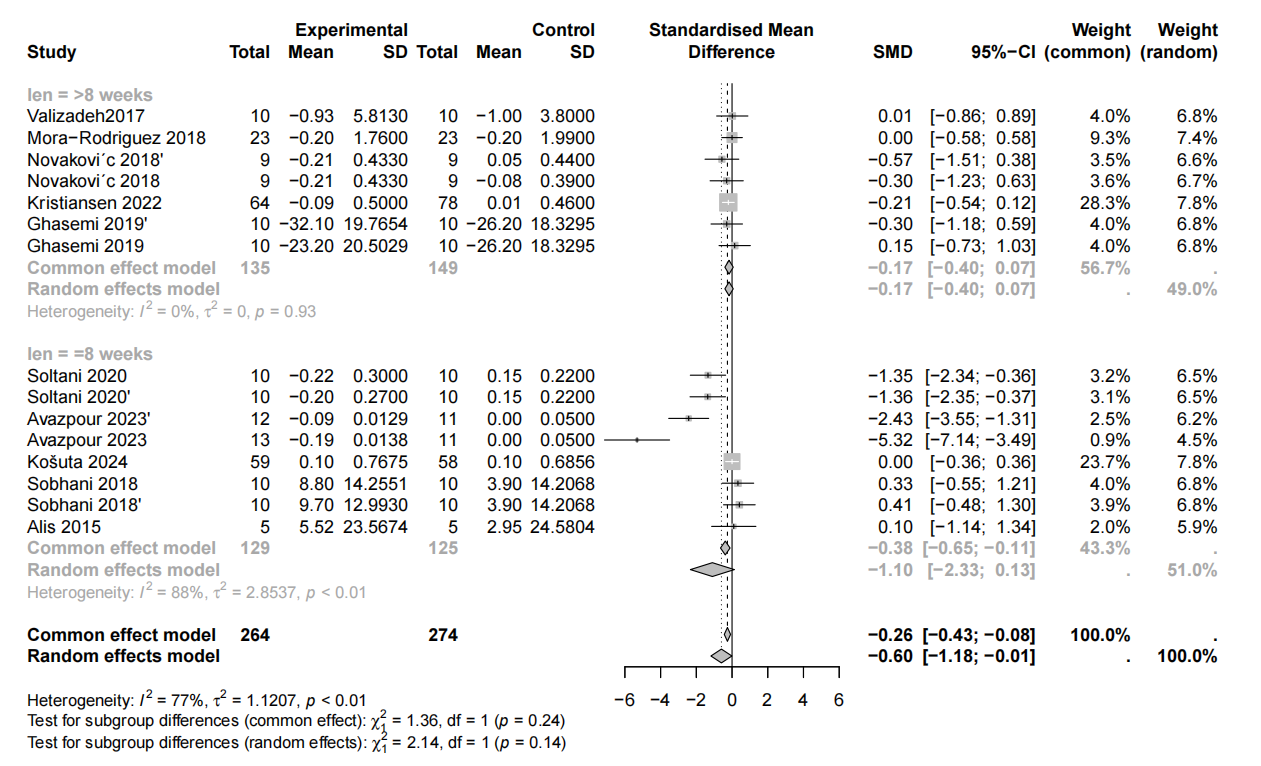


**Appendix 8-2** Subgroup Analysis of ≤8 weeks vs. >8 weeks HIIT on FIB.


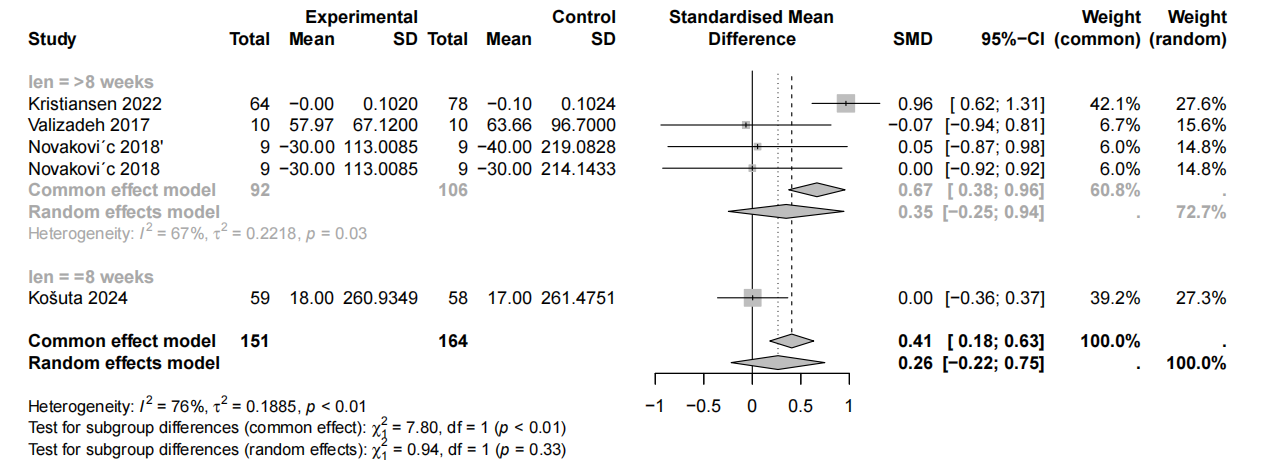


**Appendix 8-3** Subgroup Analysis of ≤8 weeks vs. >8 weeks HIIT on D-D.


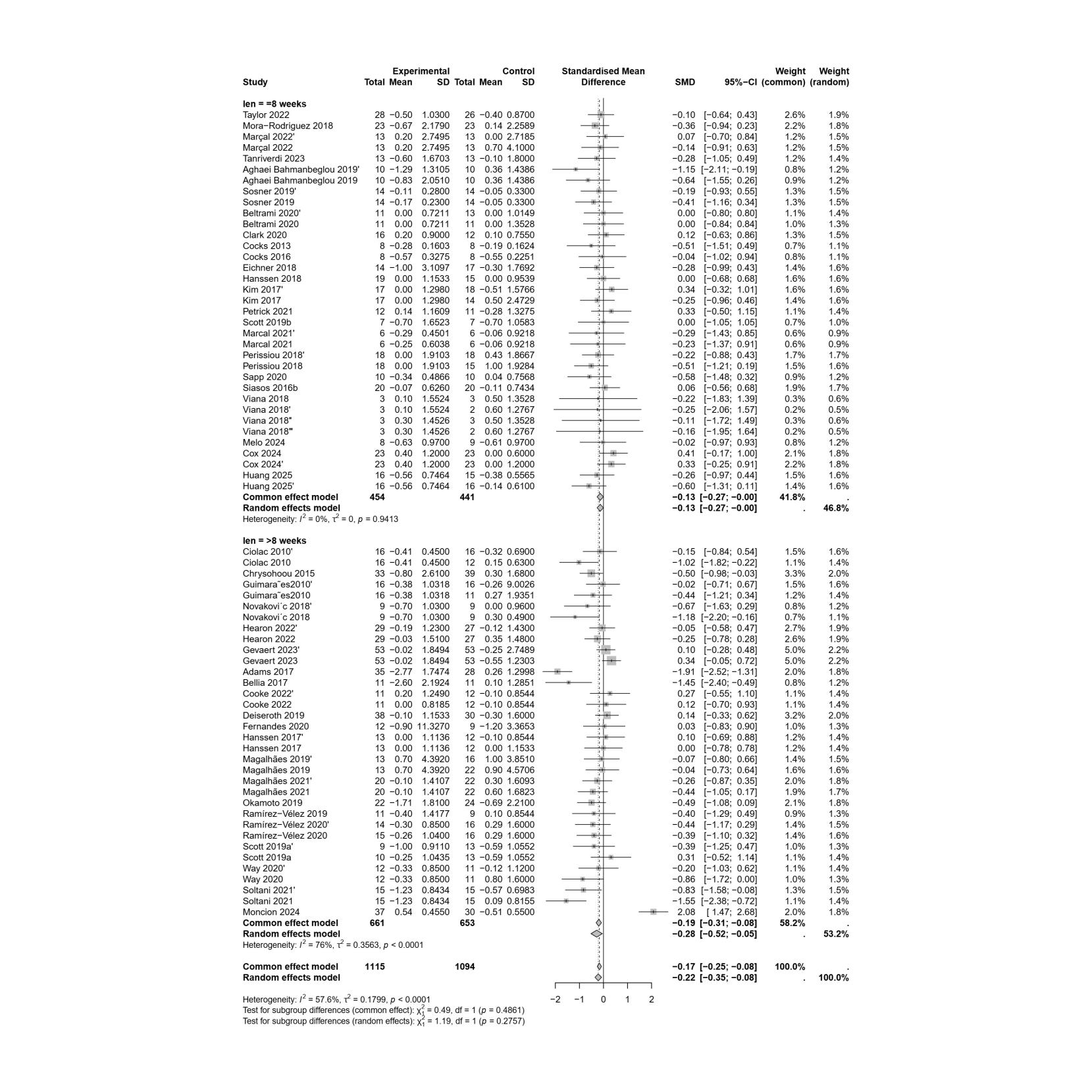


**Appendix 8-4** Subgroup Analysis of ≤8 weeks vs. >8 weeks HIIT on cfPWV.


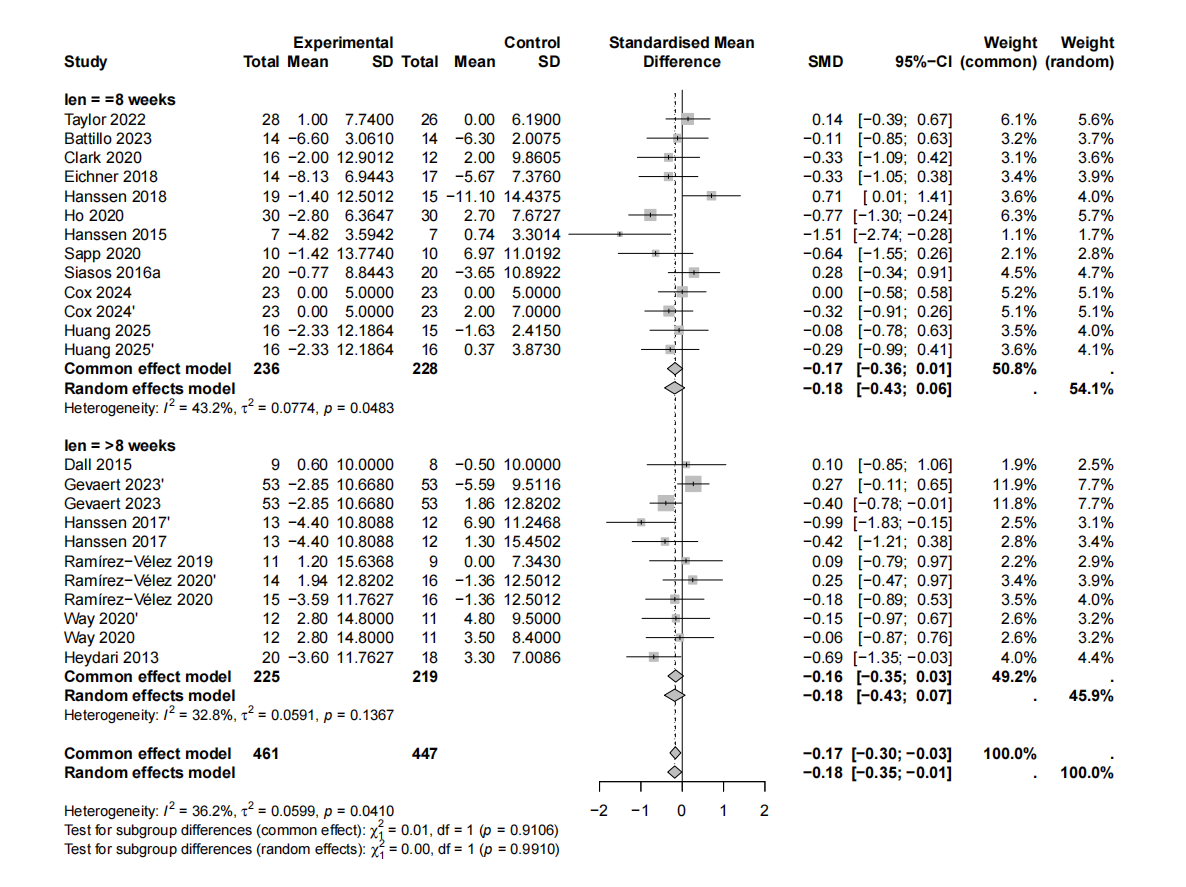


**Appendix 8-5** Subgroup Analysis of ≤8 weeks vs. >8 weeks HIIT on AIx.


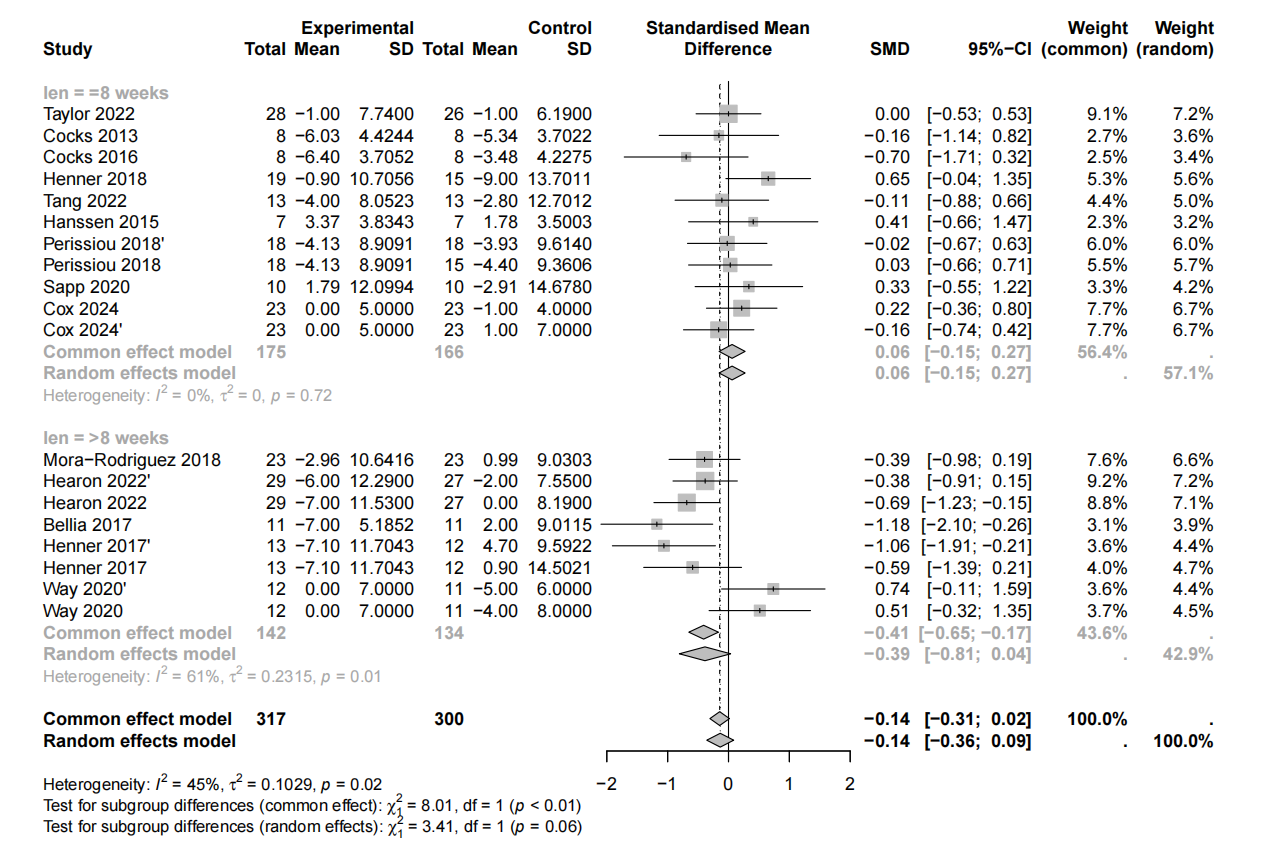


**Appendix 8-6** Subgroup Analysis of ≤8 weeks vs. >8 weeks HIIT on AIx@75HR.


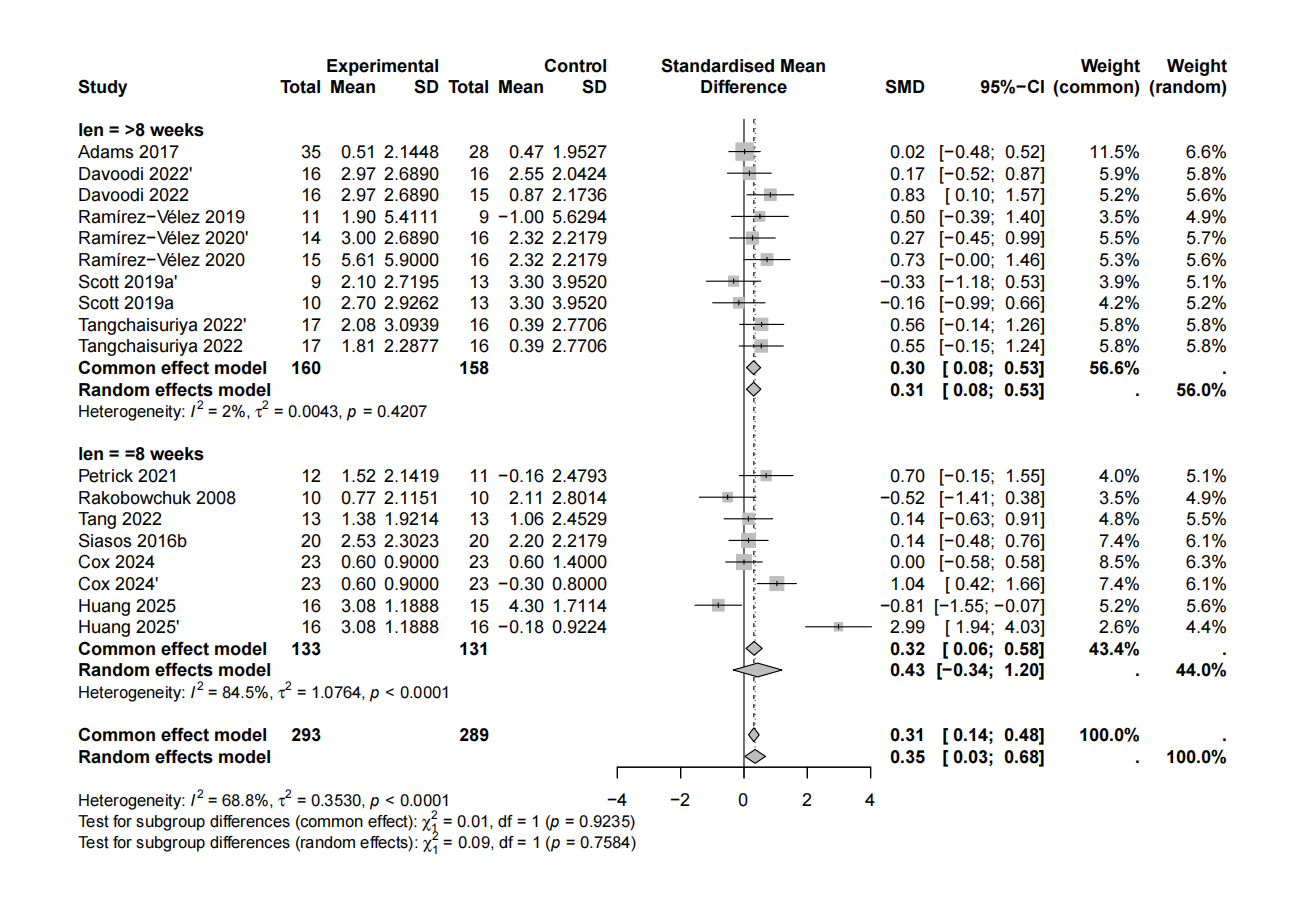


**Appendix 8-7** Subgroup Analysis of ≤8 weeks vs. >8 weeks HIIT on FMD.


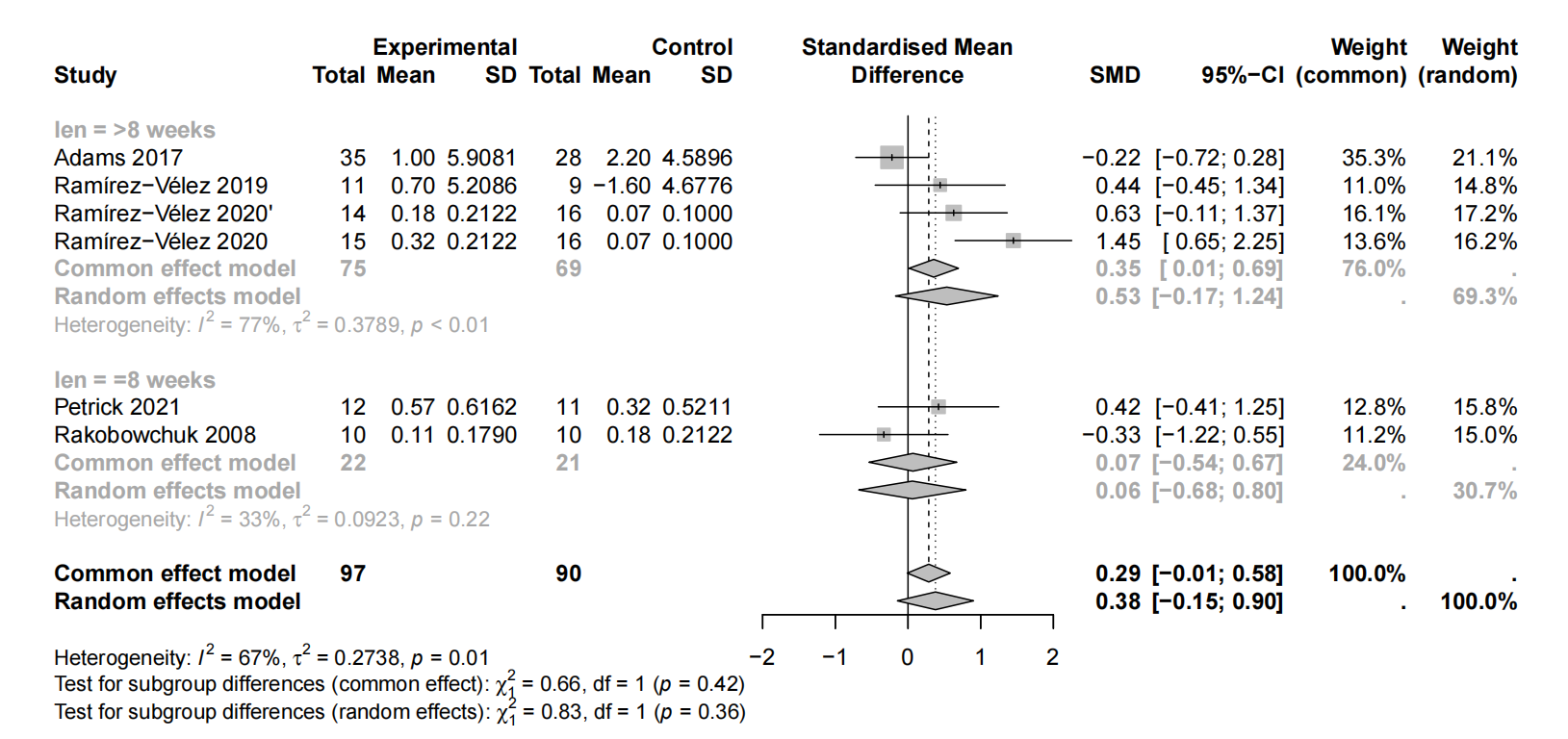


**Appendix 8-8** Subgroup Analysis of ≤8 weeks vs. >8 weeks HIIT on nFMD.


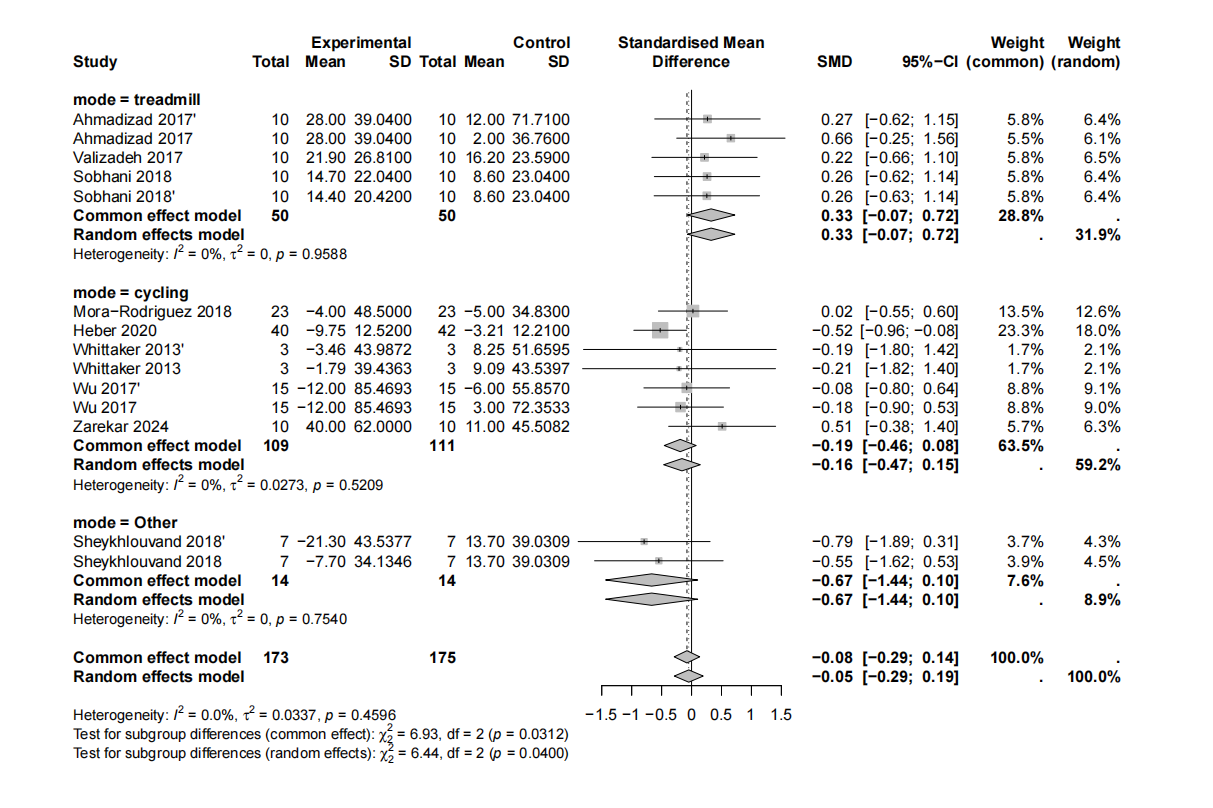


**Appendix 8-9** Subgroup Analysis of Treadmill/Cycling/Other Equipment-Based HIIT on PLT.


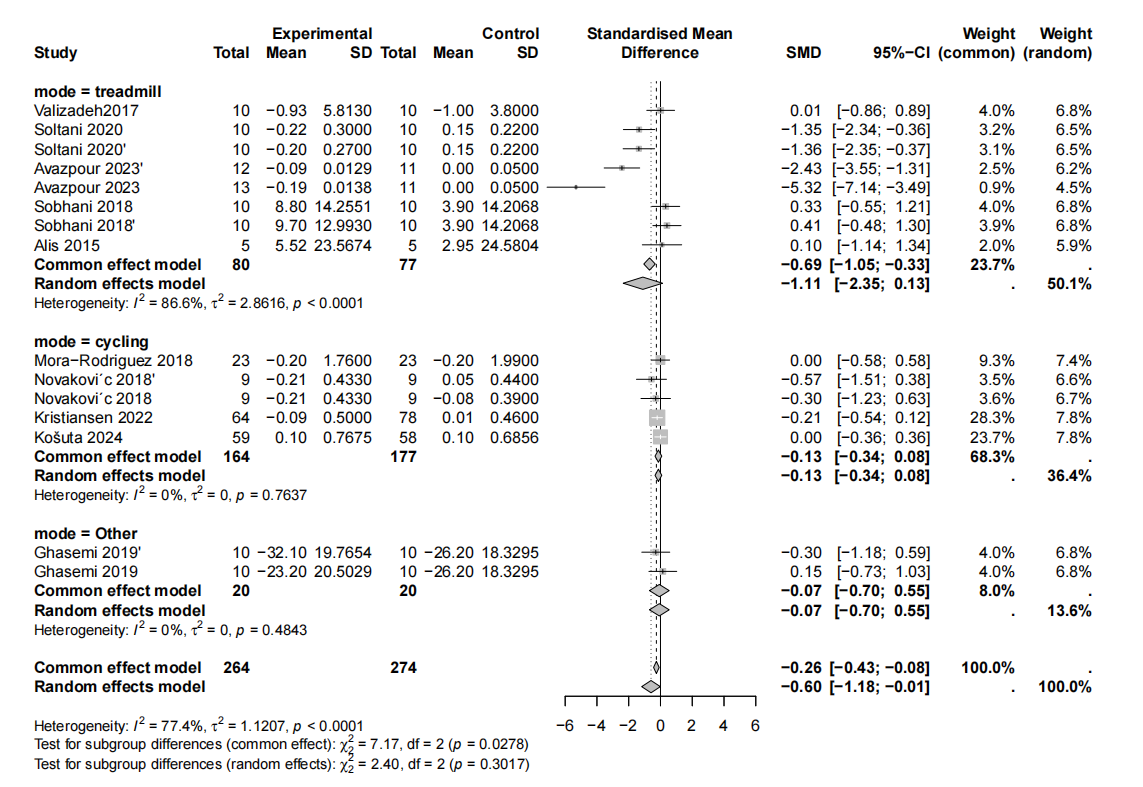


**Appendix 8-10** Subgroup Analysis of Treadmill/Cycling/Other Equipment-Based HIIT on FIB.


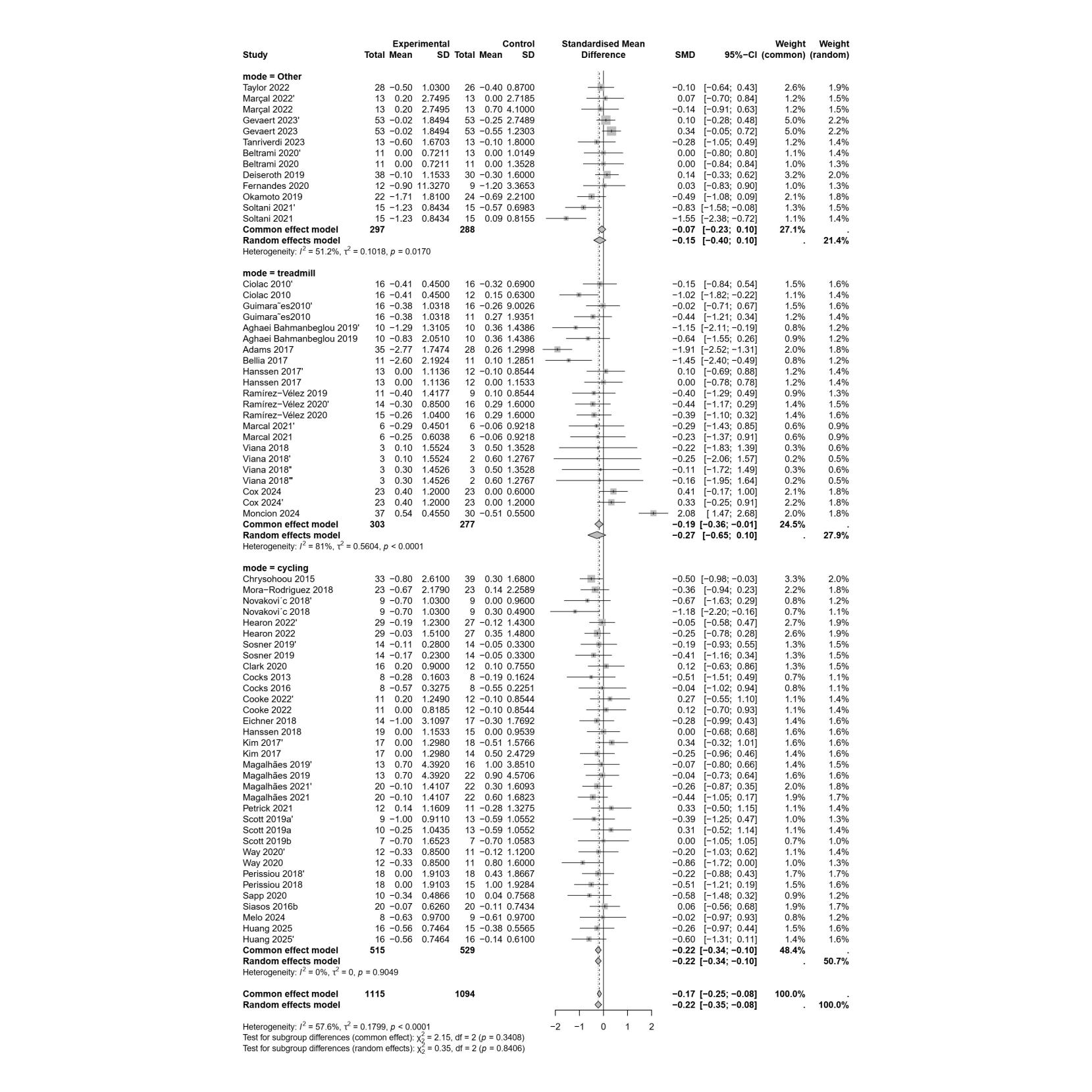


**Appendix 8-11** Subgroup Analysis of Treadmill/Cycling/Other Equipment-Based HIIT on cfPWV.


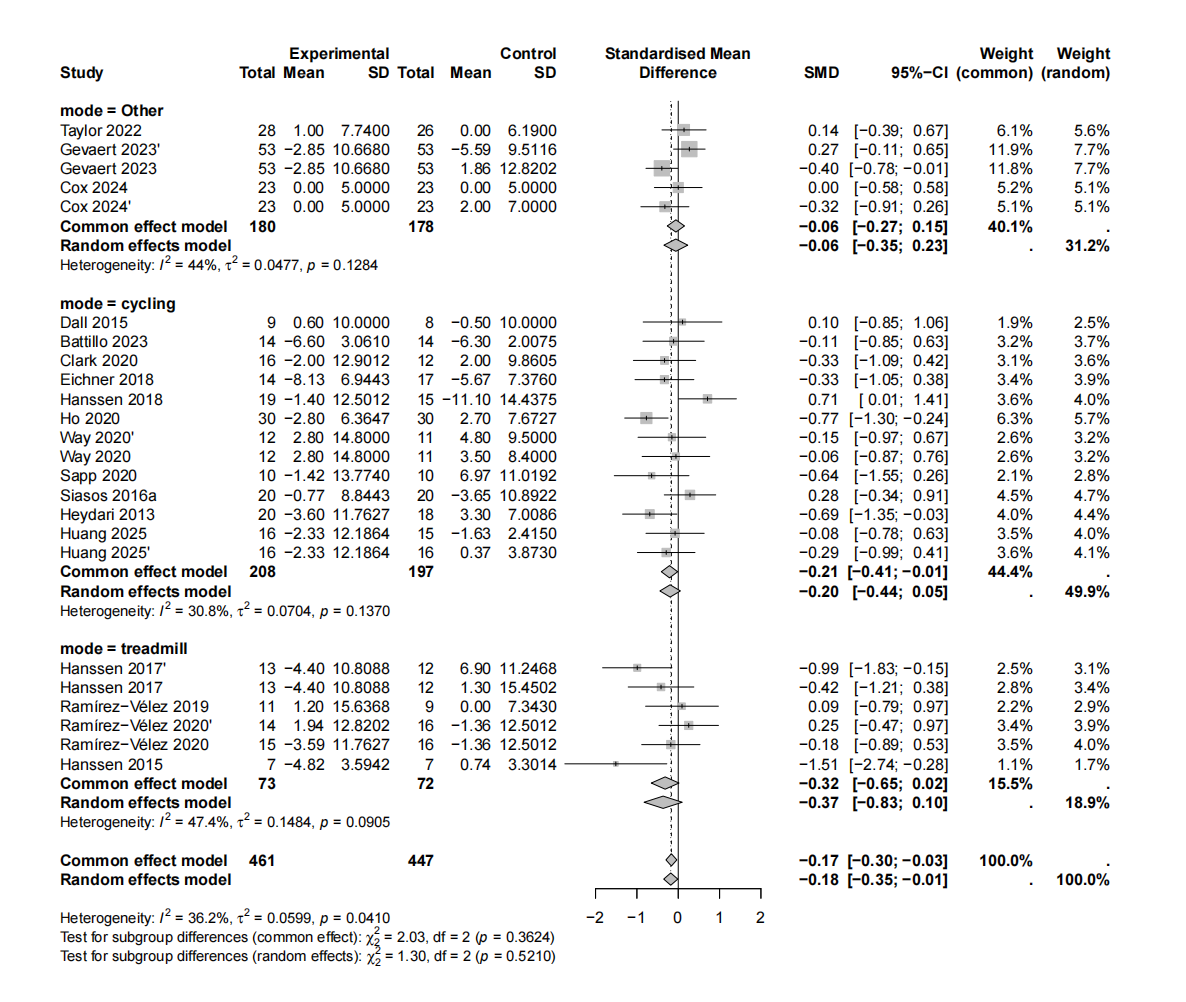


**Appendix 8-12** Subgroup Analysis of Treadmill/Cycling/Other Equipment-Based HIIT on AIx.


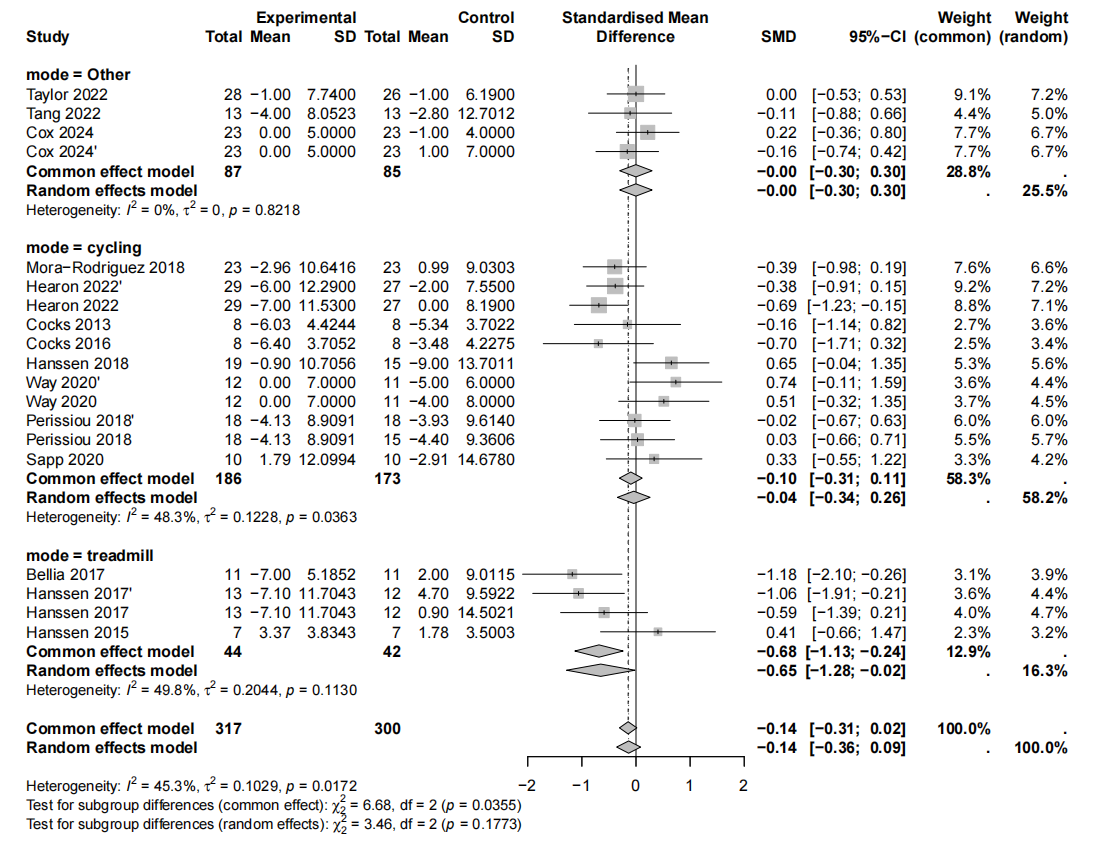


**Appendix 8-13** Subgroup Analysis of Treadmill/Cycling/Other Equipment-Based HIIT on AIx@75HR.


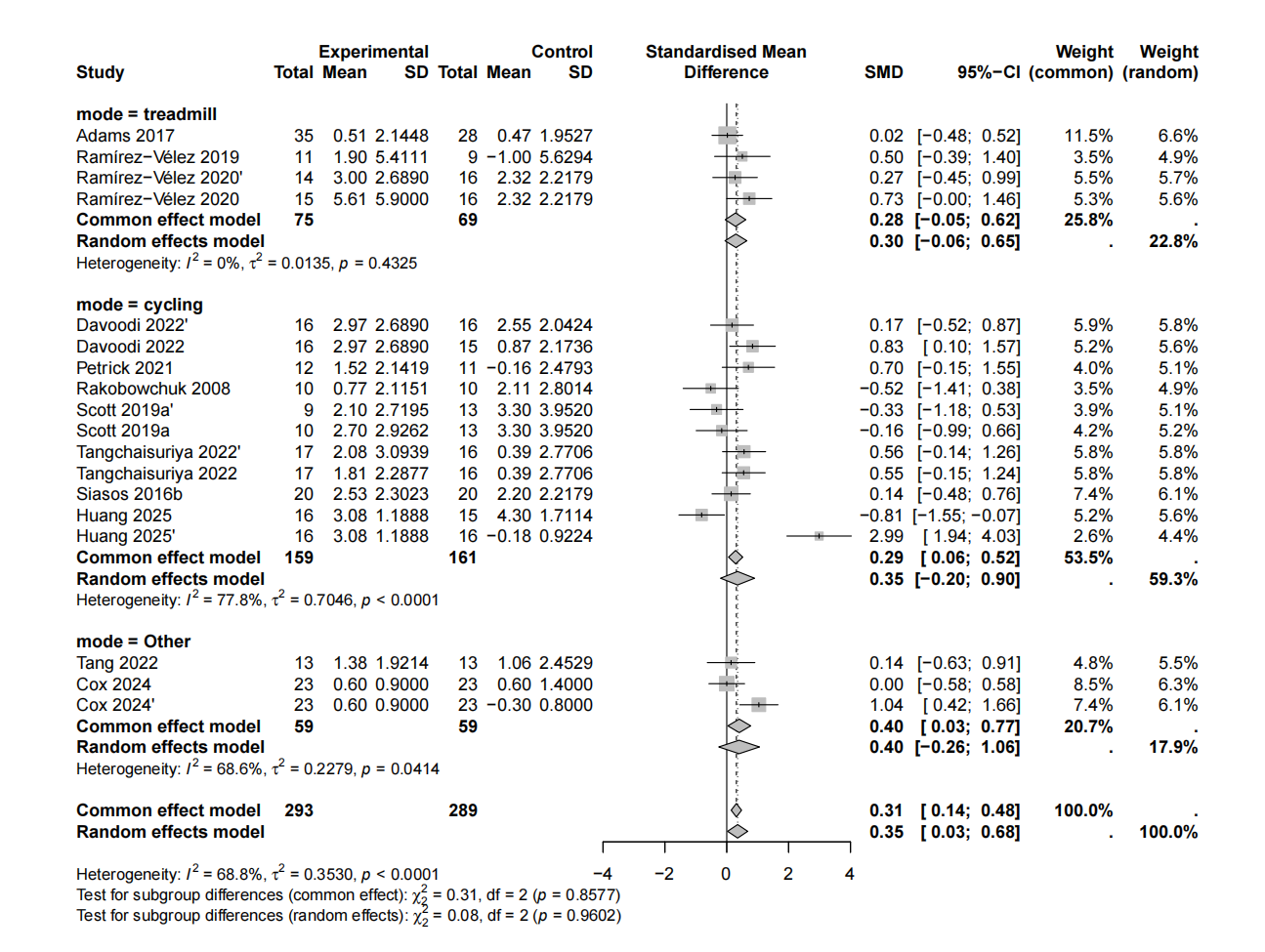


**Appendix 8-14** Subgroup Analysis of Treadmill/Cycling/Other Equipment-Based HIIT on FMD.

**Appendix 9.** Sensitivity analysis results.


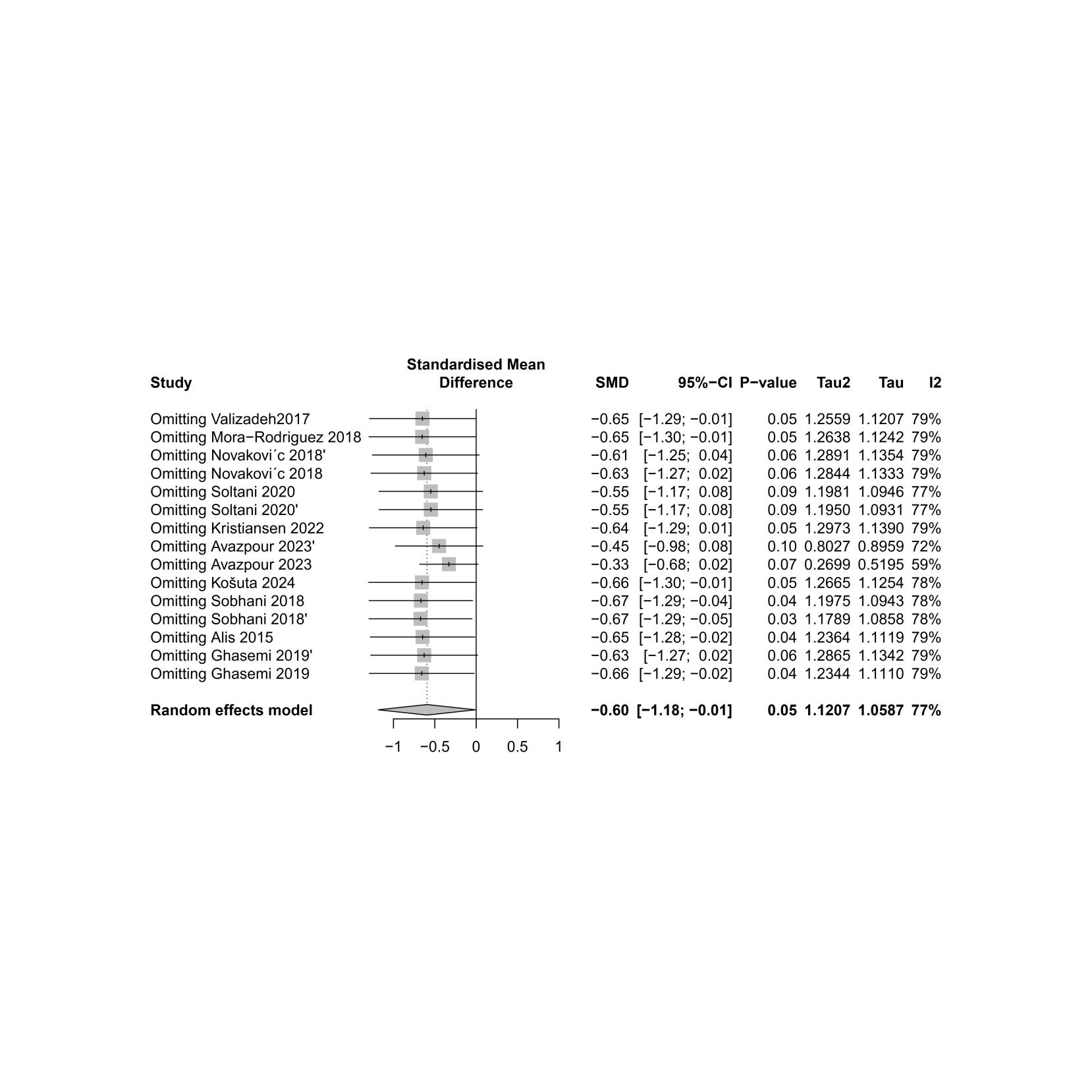


**Appendix 9A** Sensitivity analysis of FIB.


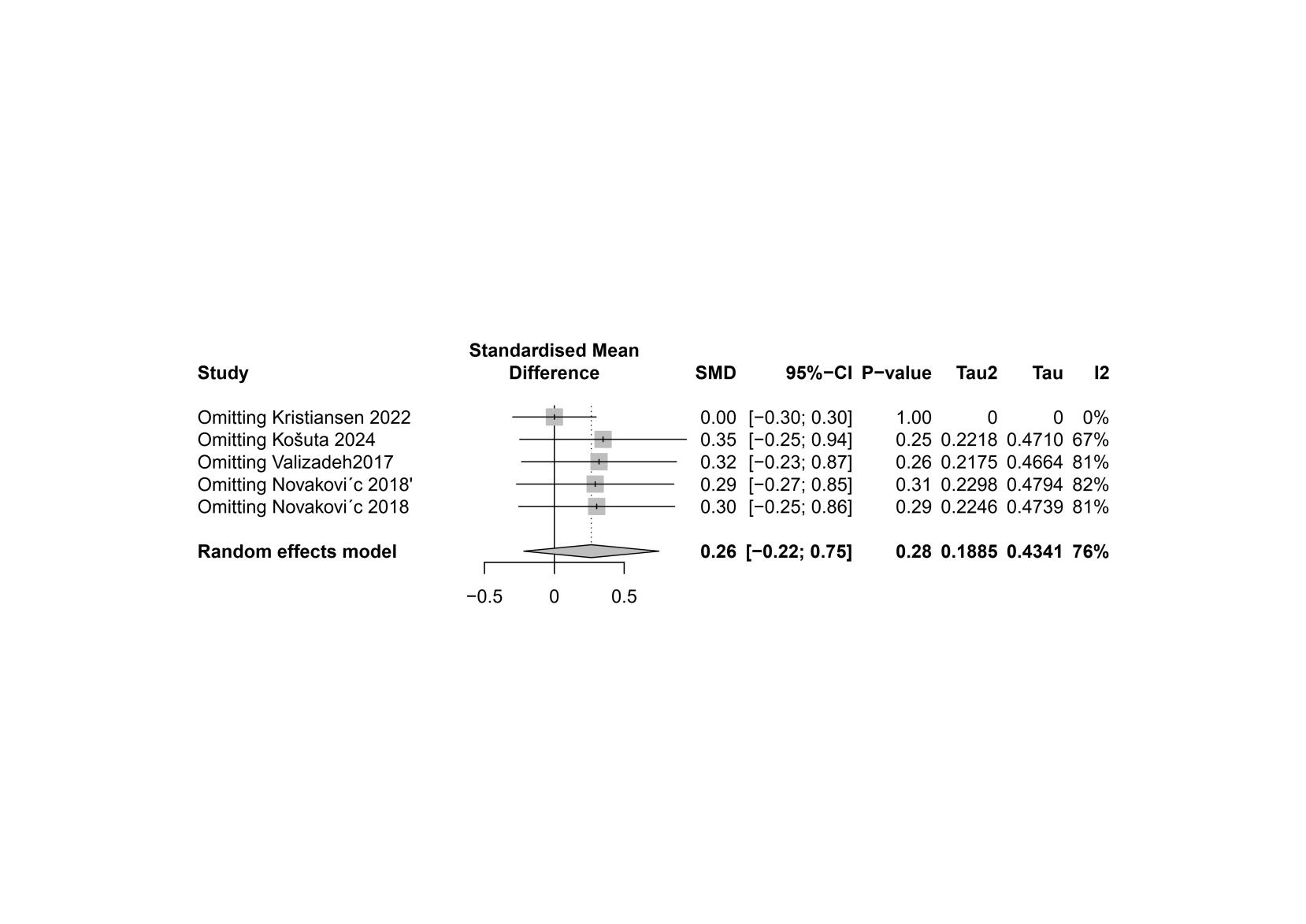


**Appendix 9B** Sensitivity analysis of D-D.


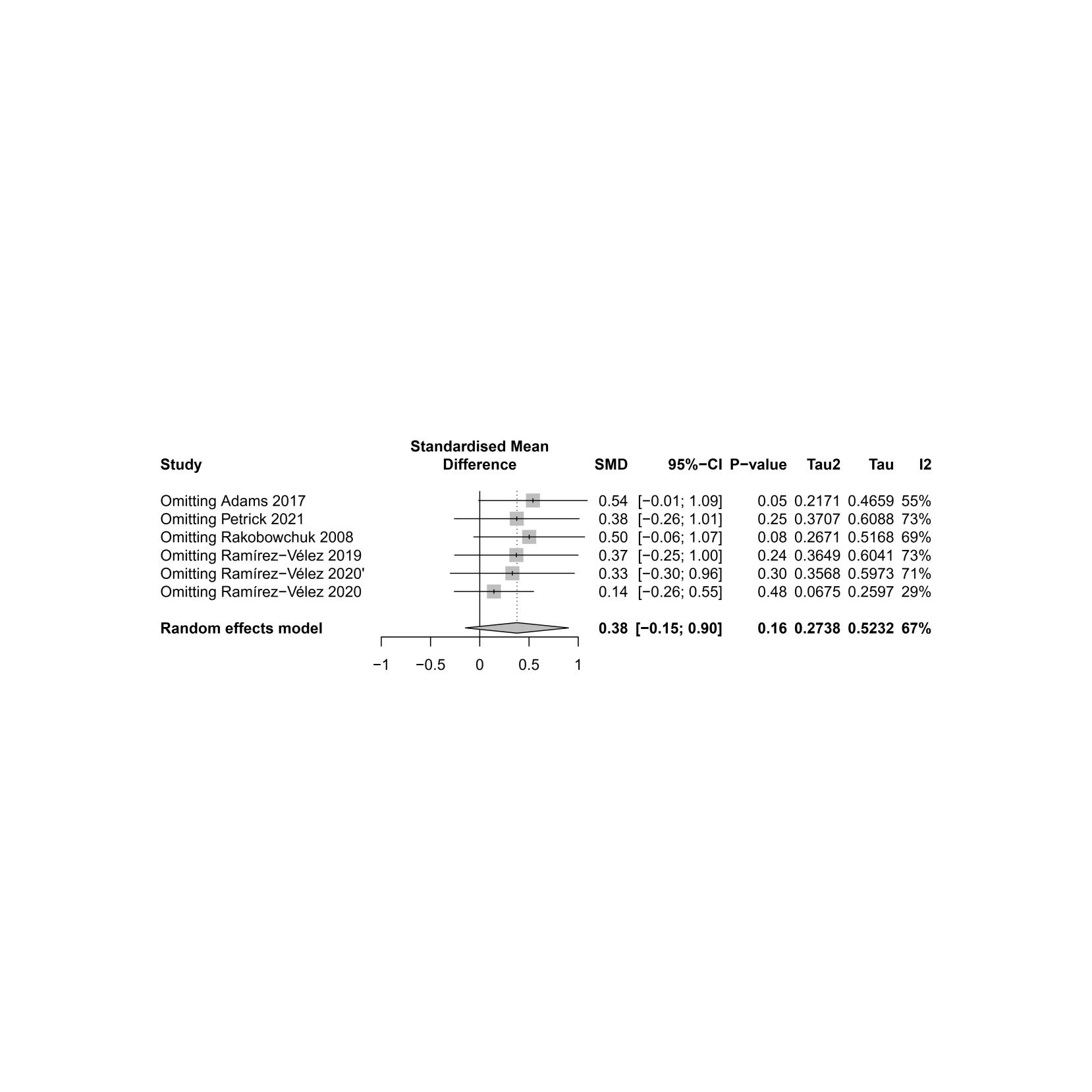


**Appendix 9C** Sensitivity analysis of nFMD.


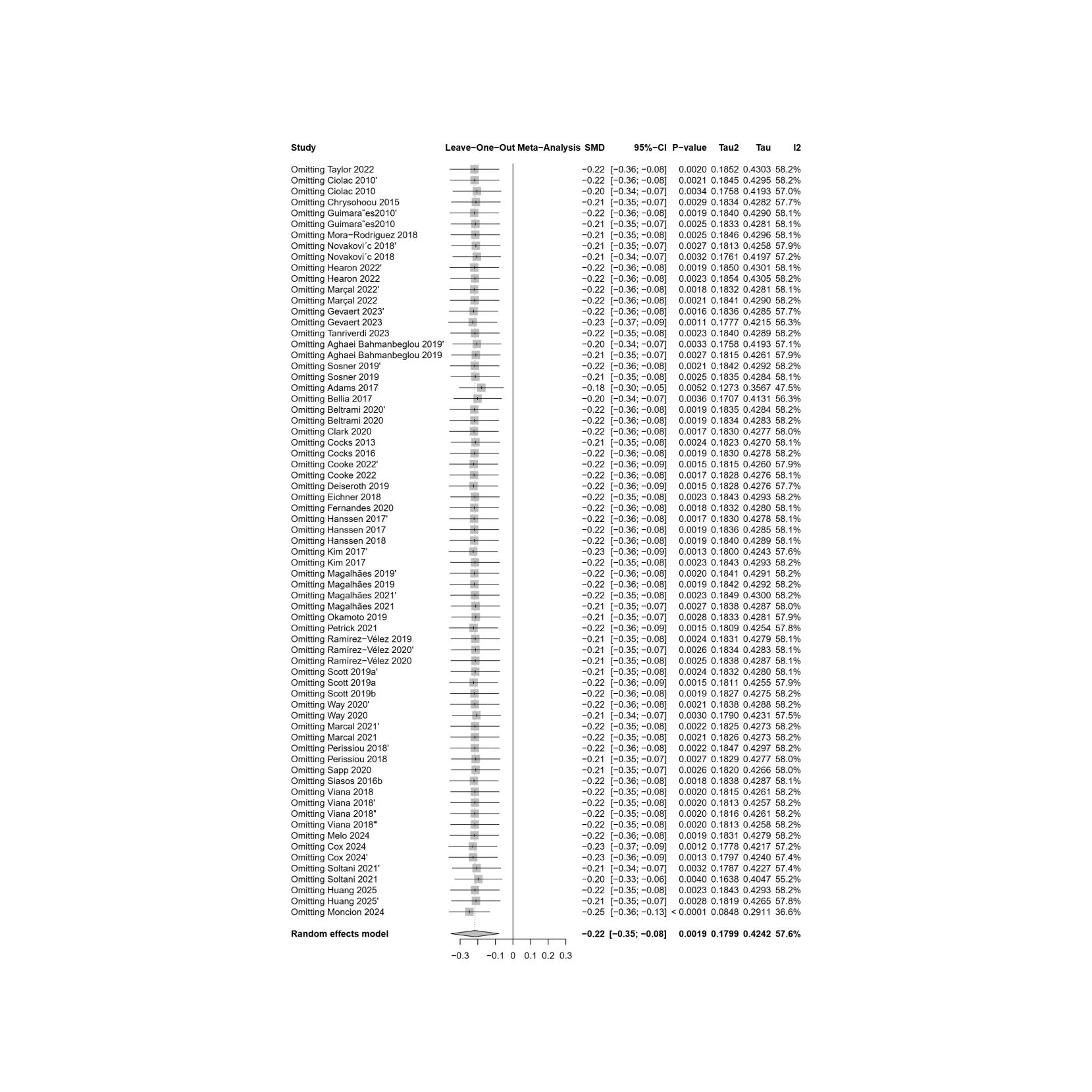


**Appendix 9D** Sensitivity analysis of cfPWV.


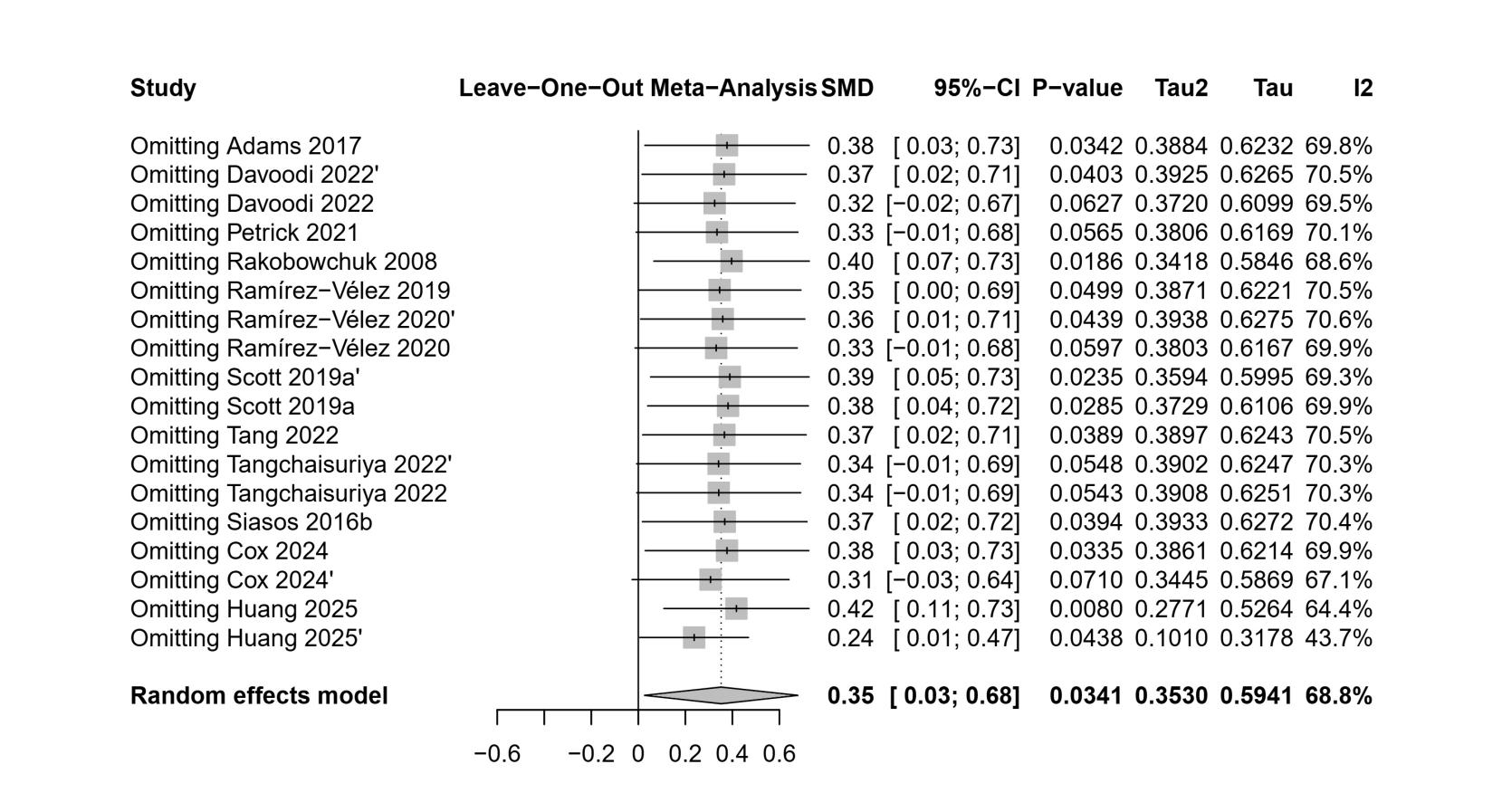


**Appendix 9E** Sensitivity analysis of FMD.


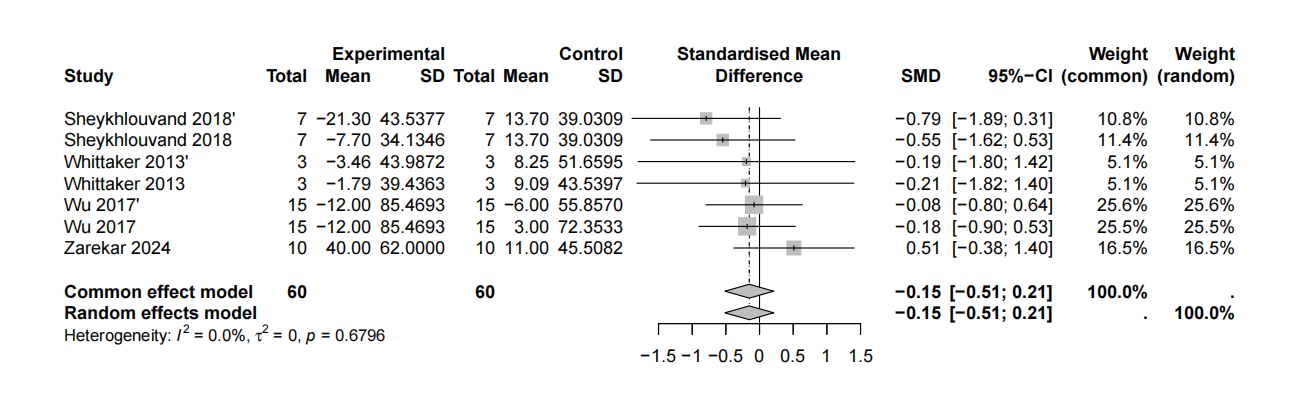


**Appendix 9F** Sensitivity Analysis of PLT Indicators Due to Missing Gender Data.


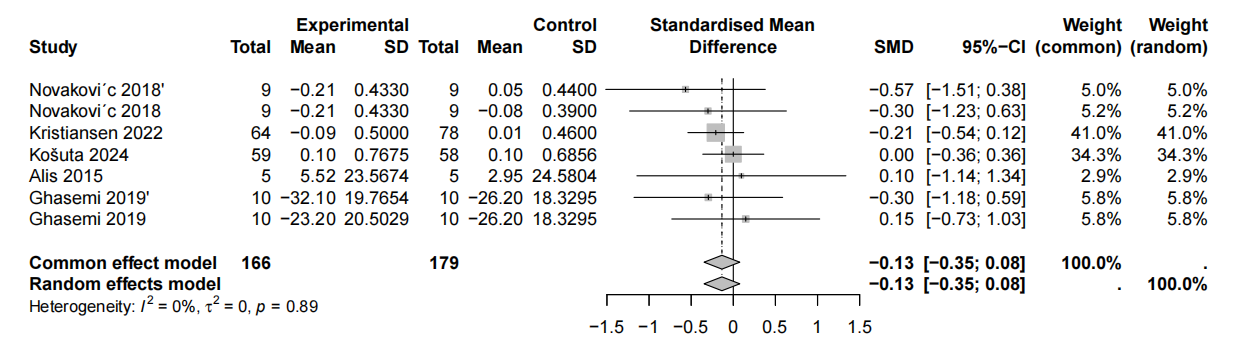


**Appendix 9G** Sensitivity Analysis of FIB Indicators Due to Missing Gender Data.


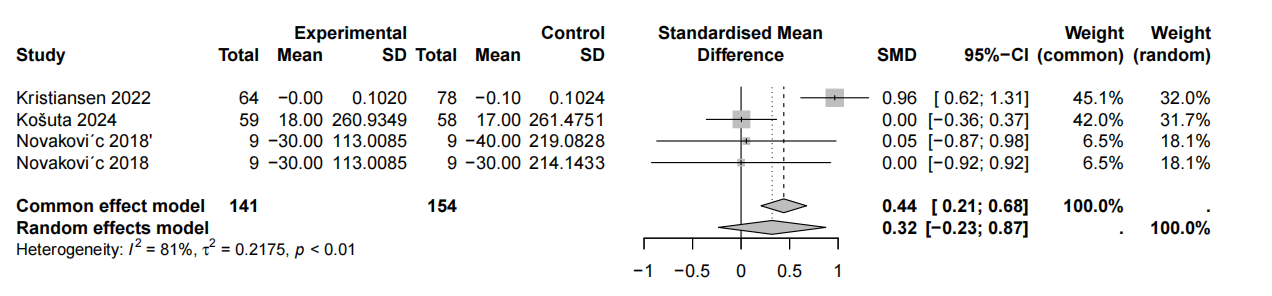


**Appendix 9H** Sensitivity Analysis of D-D Indicators Due to Missing Gender Data.


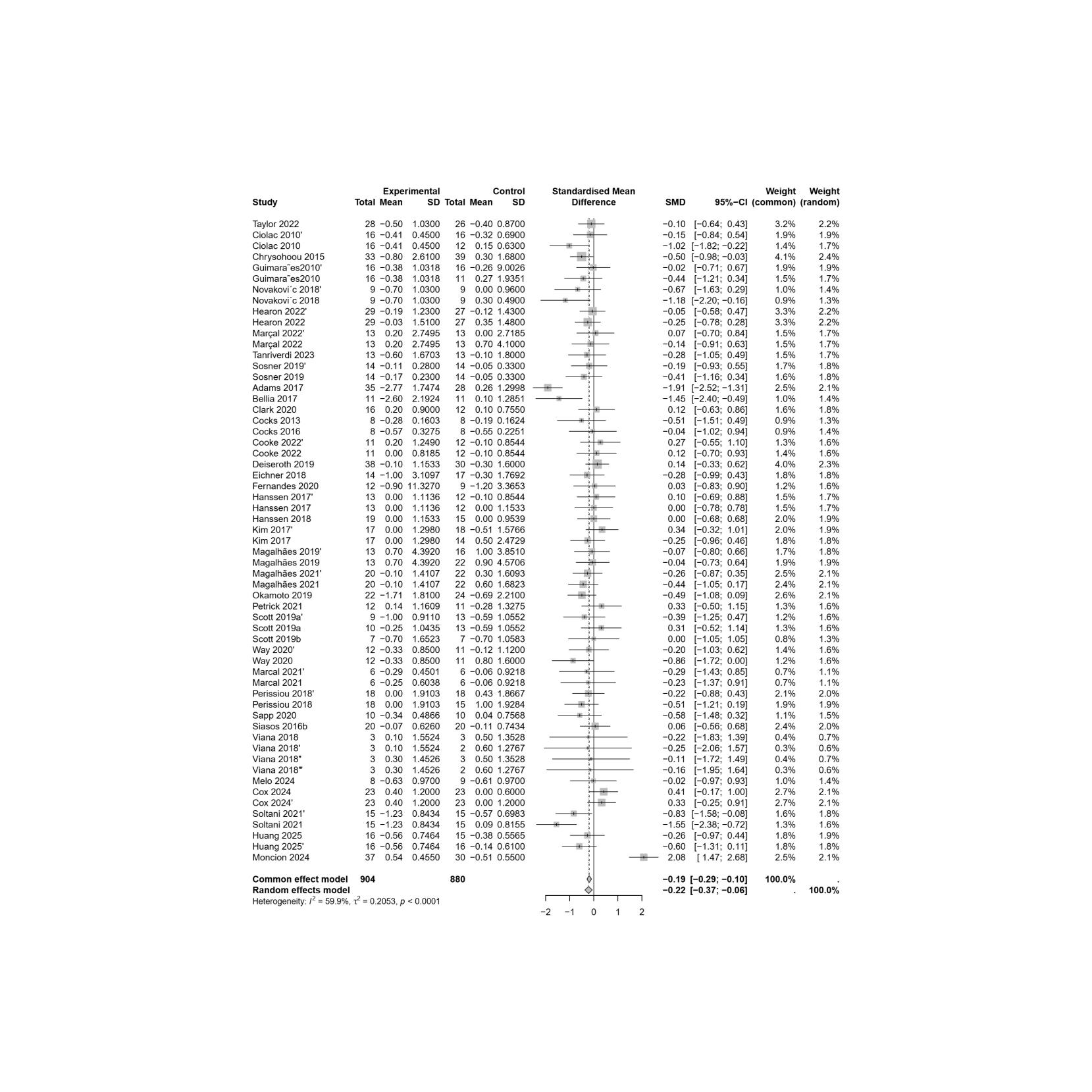


**Appendix 9I** Sensitivity Analysis of cfPWV Indicators Due to Missing Gender Data.


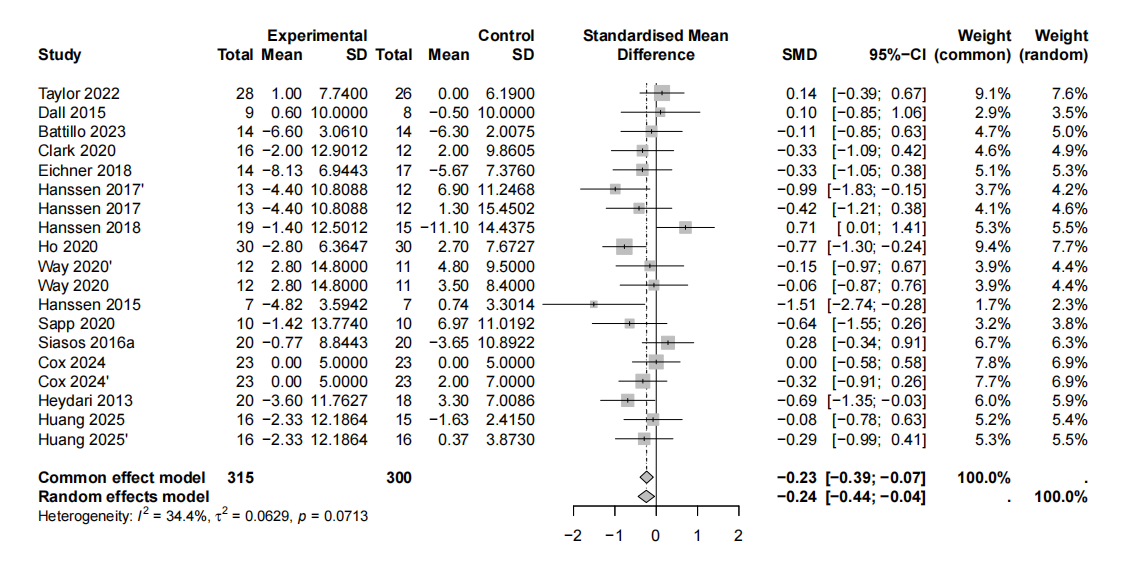


**Appendix 9J** Sensitivity Analysis of AIx Indicators Due to Missing Gender Data.


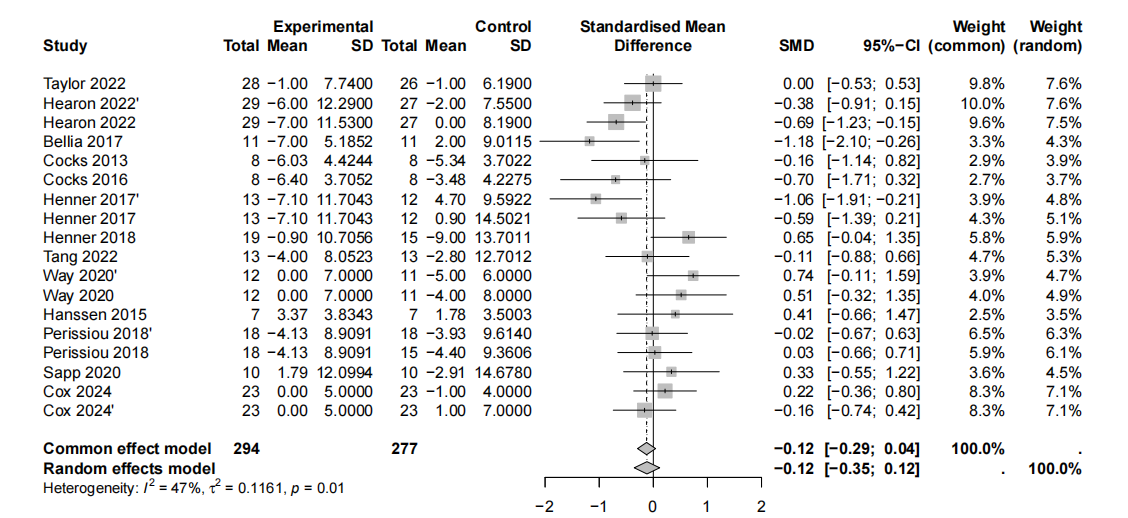


**Appendix 9K** Sensitivity Analysis of AIx@75HR Indicators Due to Missing Gender Data.


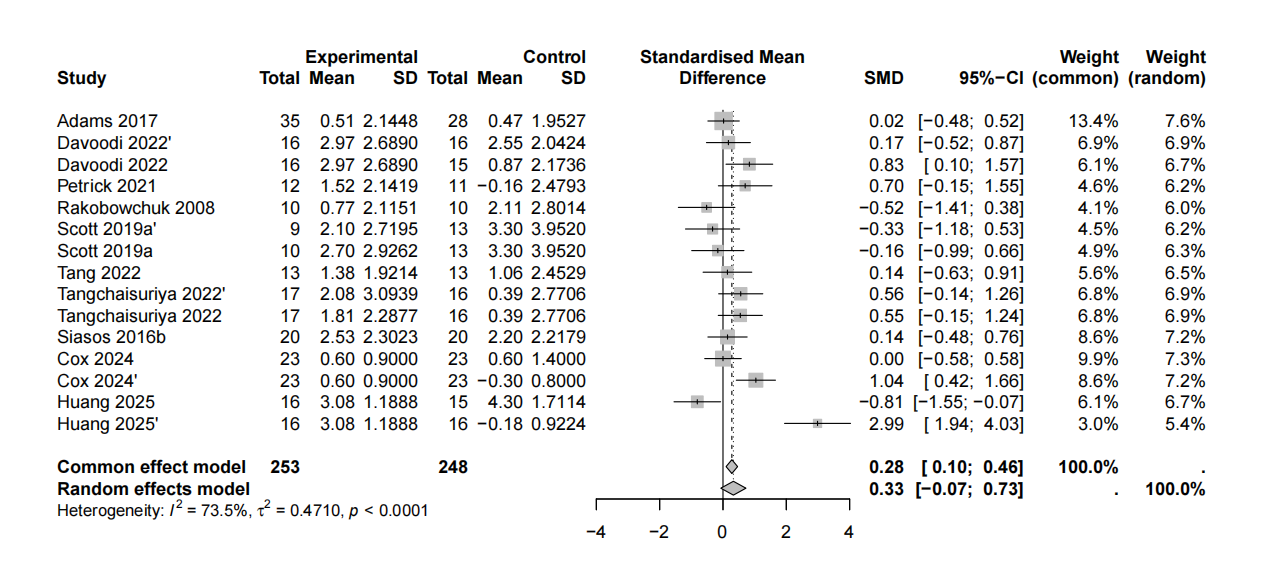


**Appendix 9L** Sensitivity Analysis of FMD Indicators Due to Missing Gender Data.


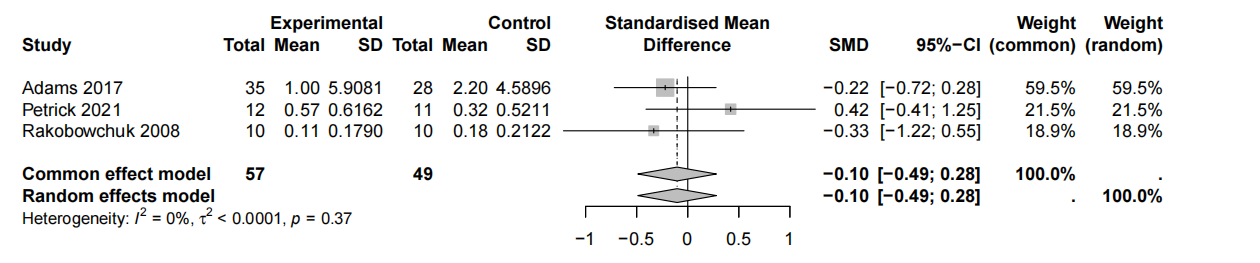


**Appendix 9M** Sensitivity Analysis of nFMD Indicators Due to Missing Gender Data.


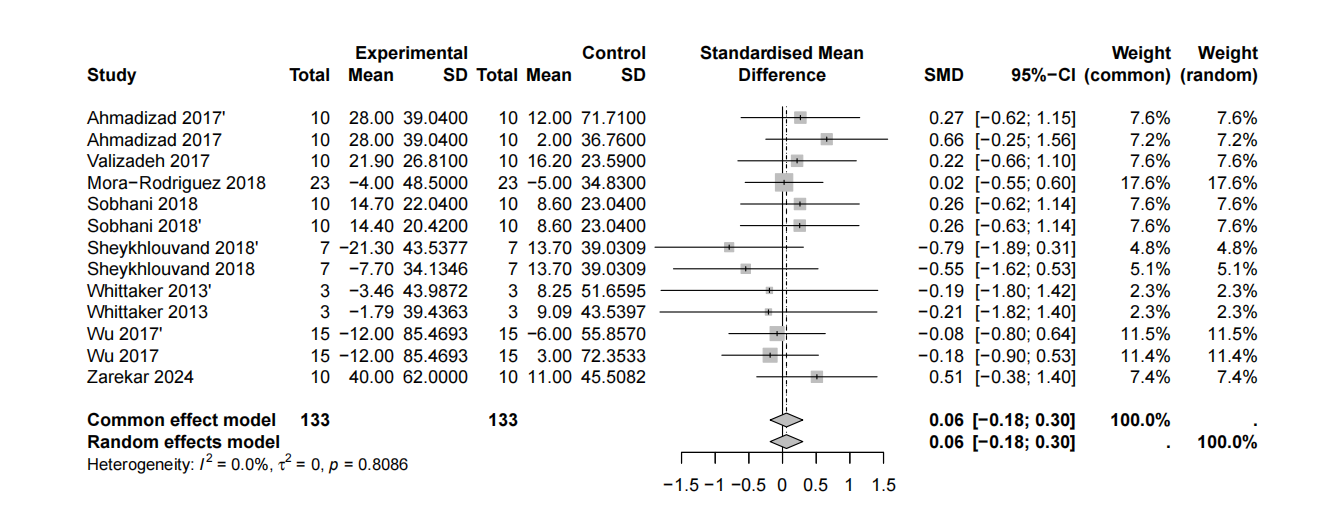


**Appendix 9N** Sensitivity Analysis of PLT Indicators When Combined with Other Exercises.


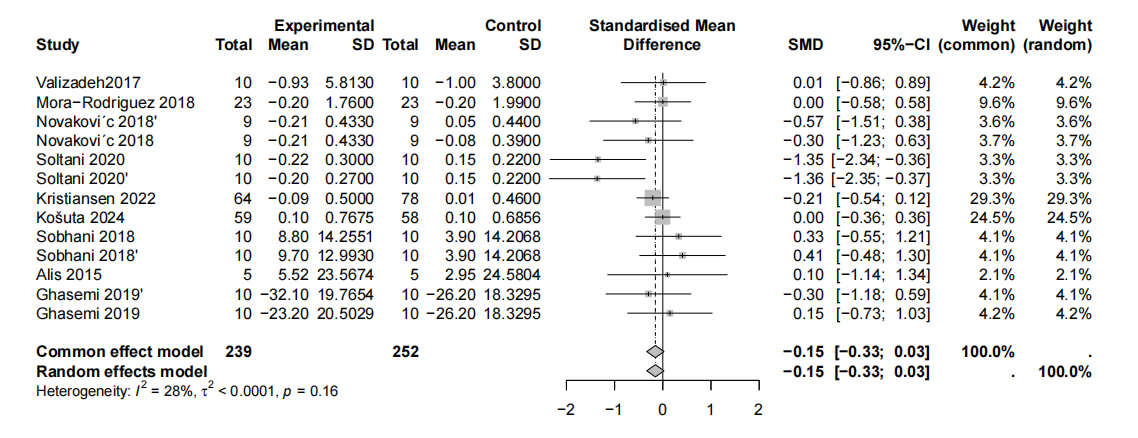


**Appendix 9O** Sensitivity Analysis of FIB Indicators When Combined with Other Exercises.


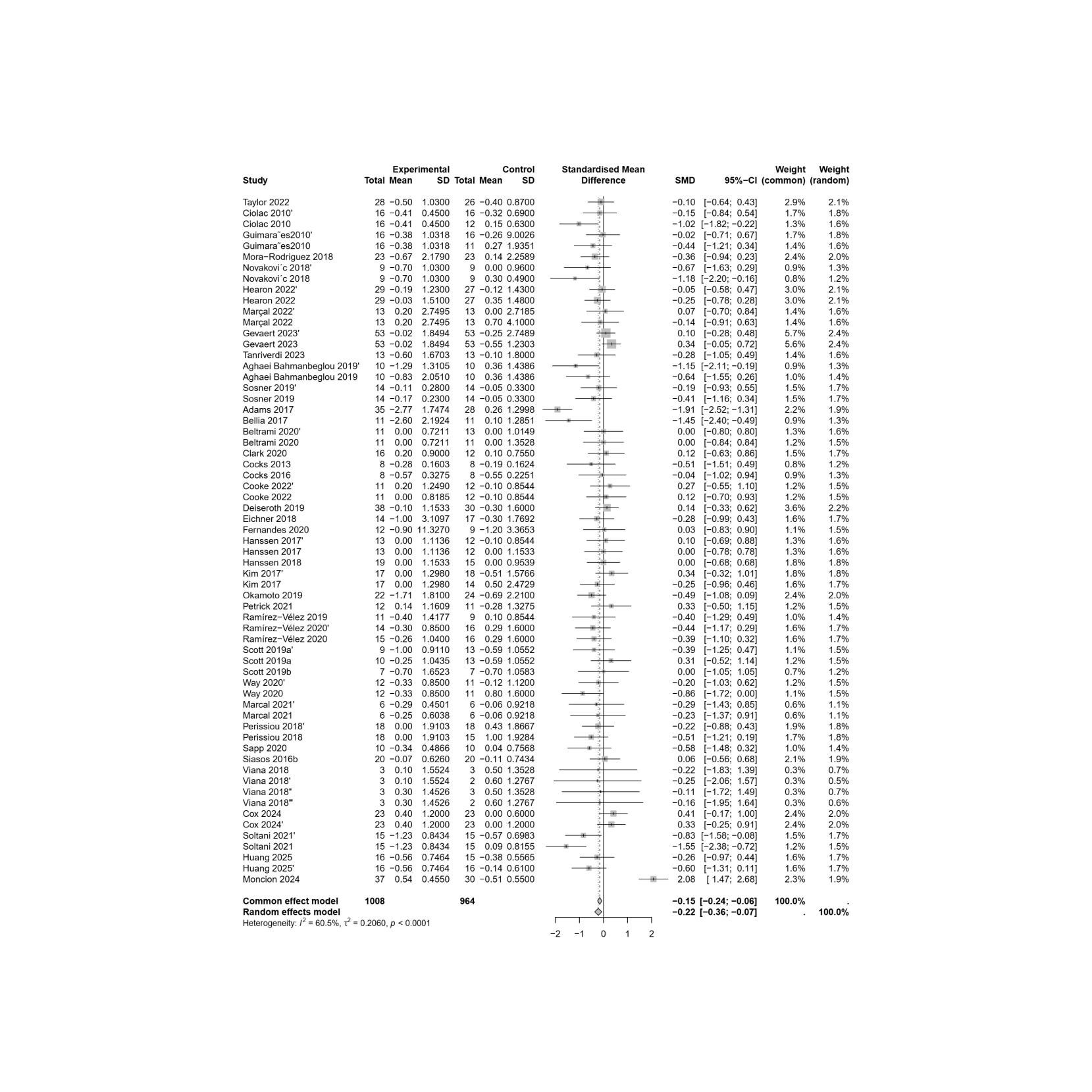


**Appendix 9P** Sensitivity Analysis of cfPWV Indicators When Combined with Other Exercises.

**Appendix 10.** Meta-regression analysis results.


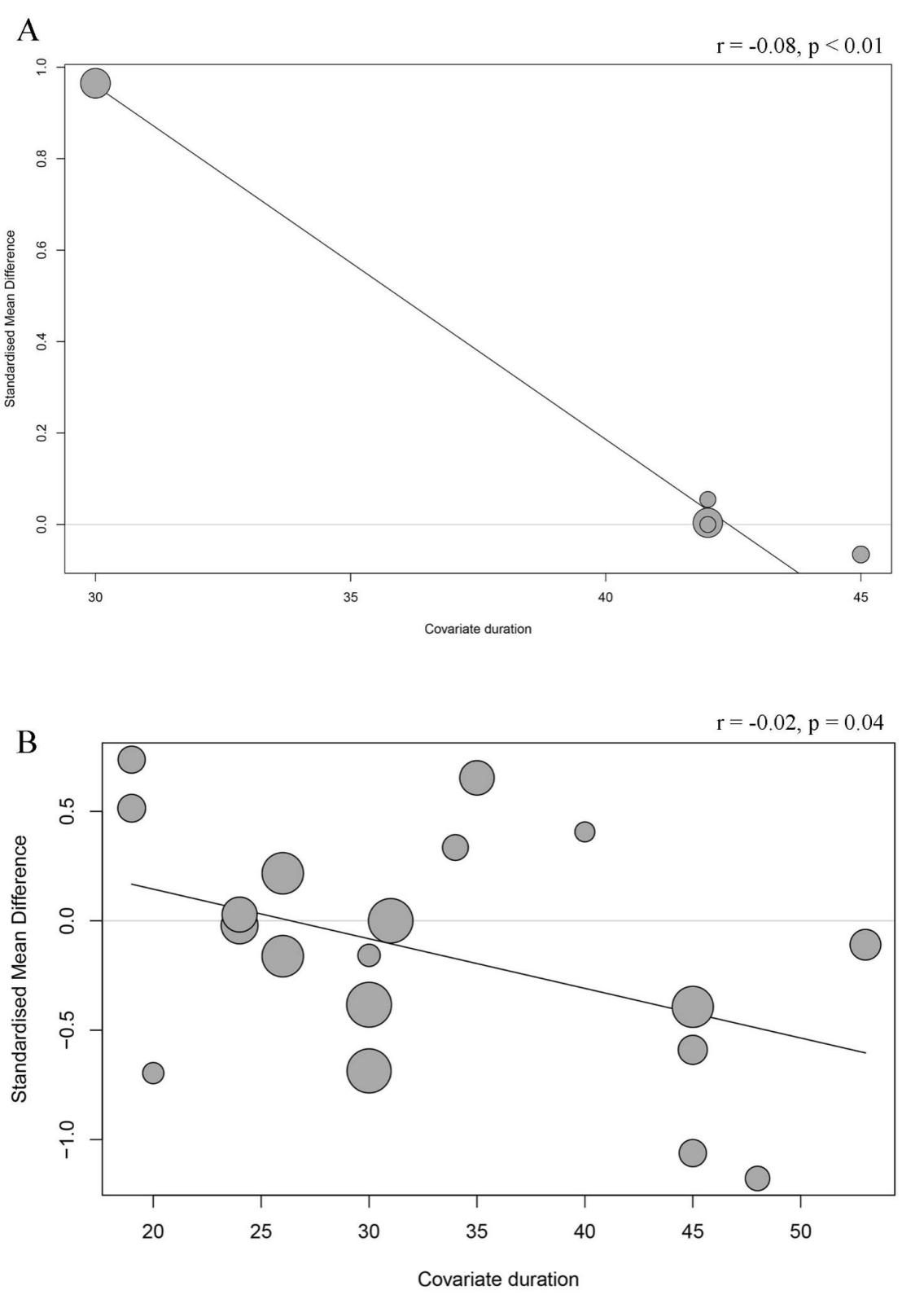


**A** Associations between HIIT training time and D-D. r=-0.08，*p<*0.01


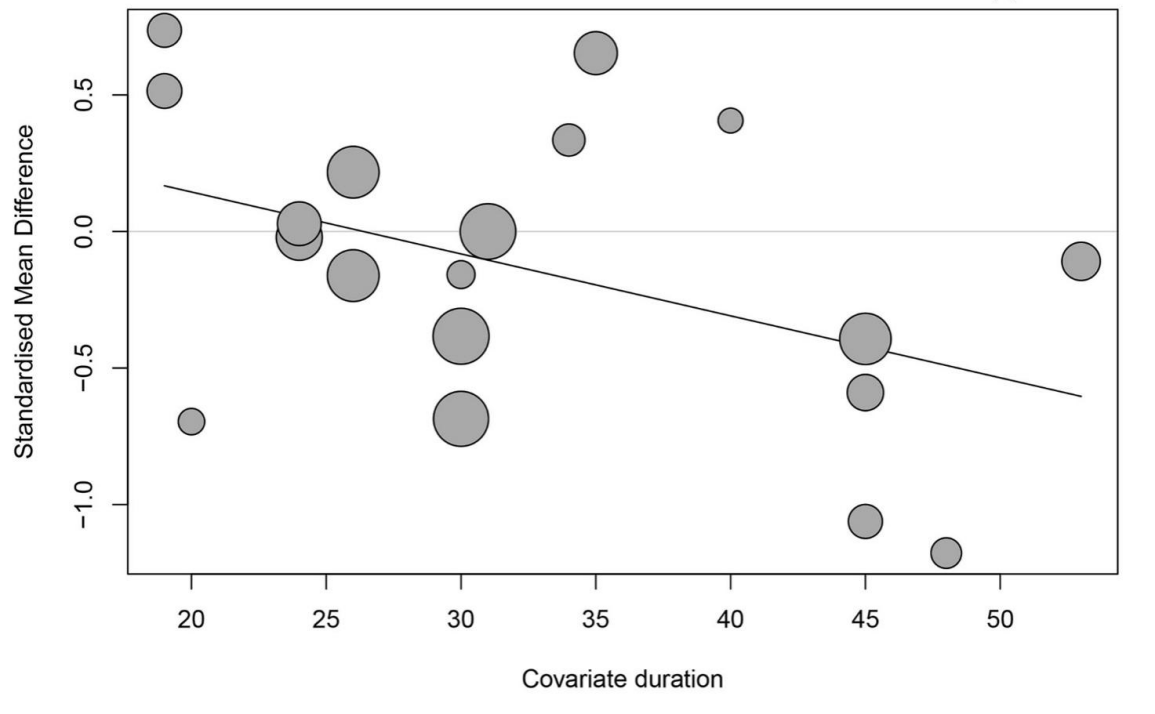


**B** Associations between HIIT training time and AIx@75HR. r=-0.02，*p=*0.04

**
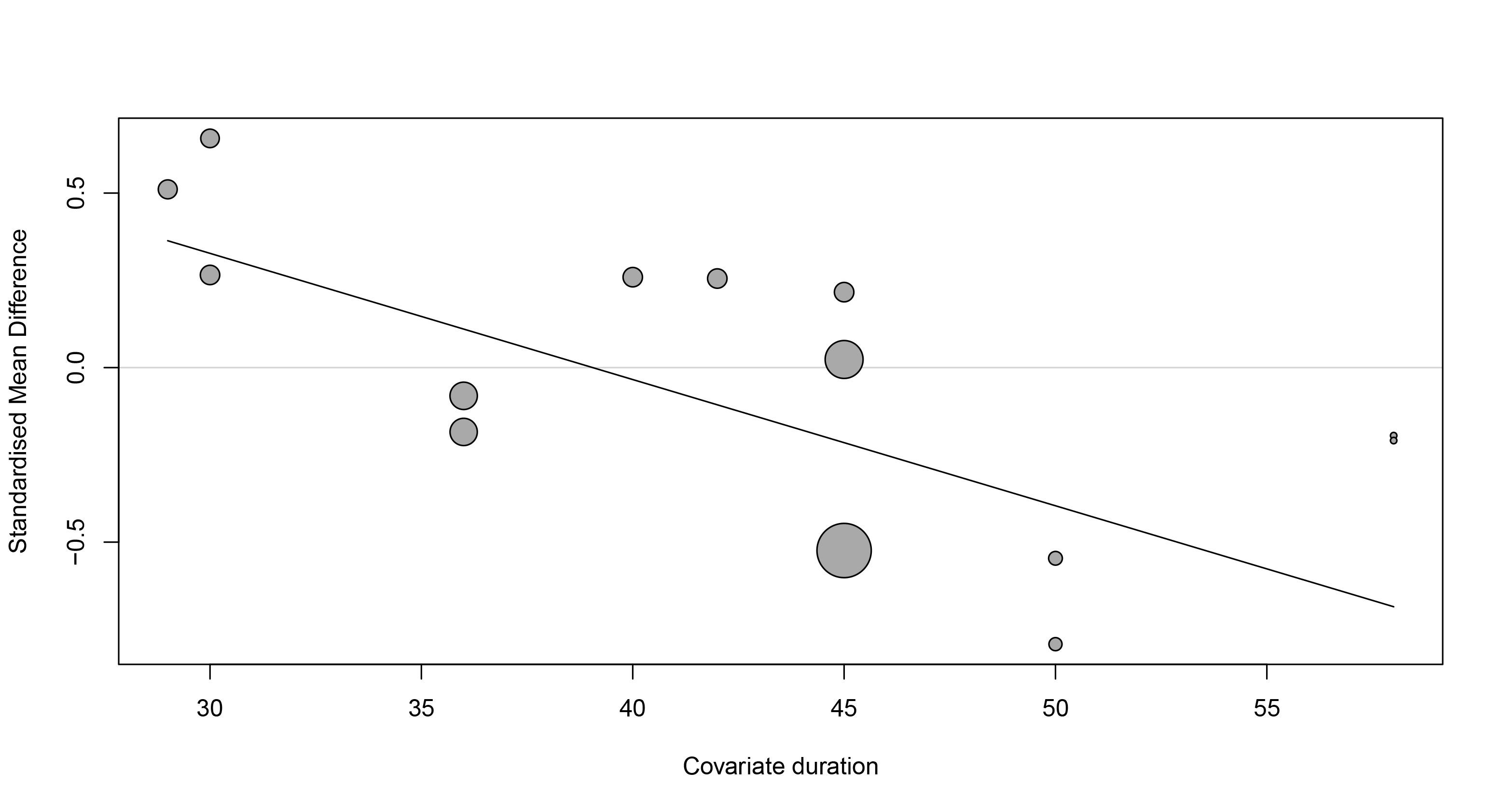
**

**C** Associations between HIIT training time and PLT. r=-0.07，*p*=0.02

**
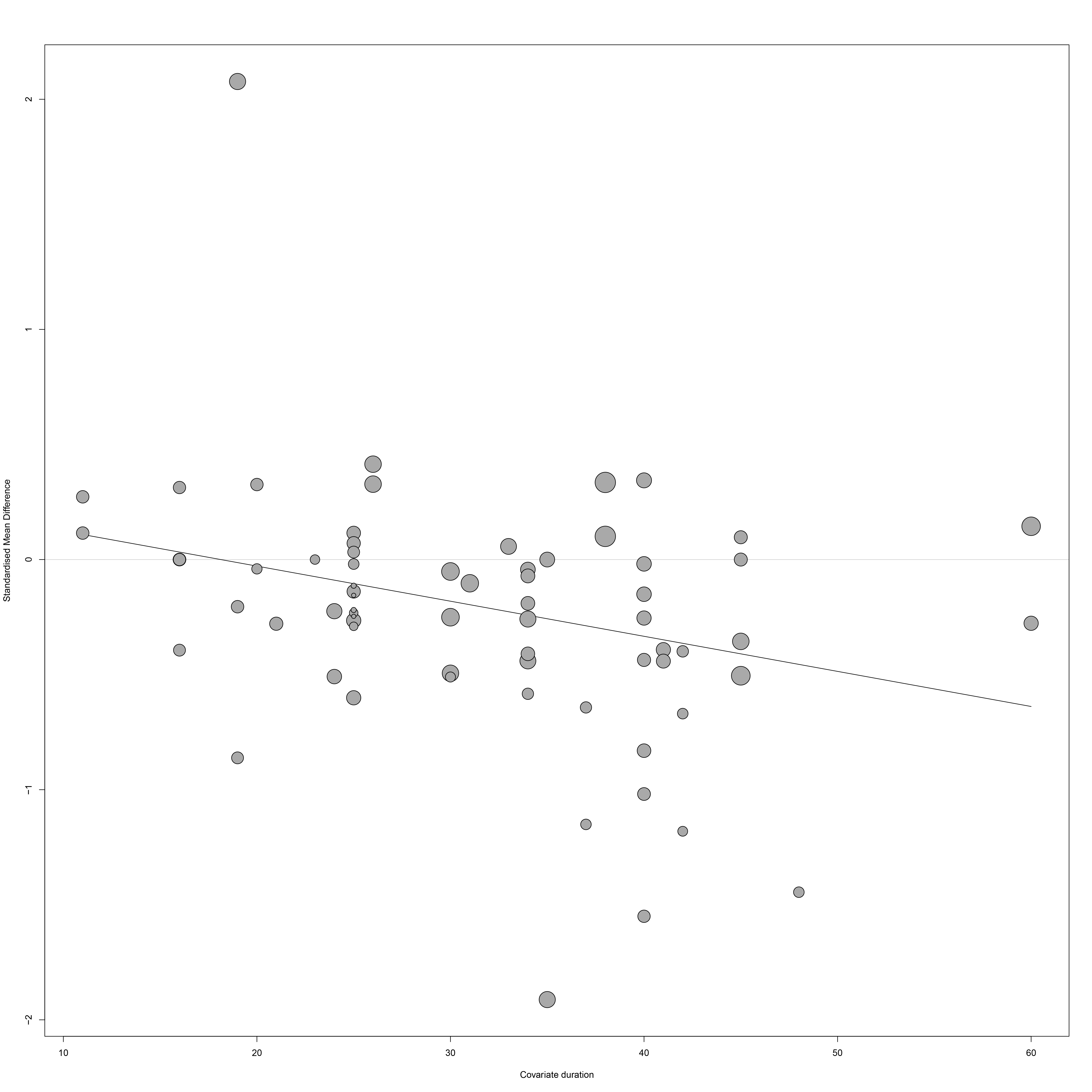
**

**D** Associations between HIIT training time and cfPWV. r=-0.03，*p*=0.02

**
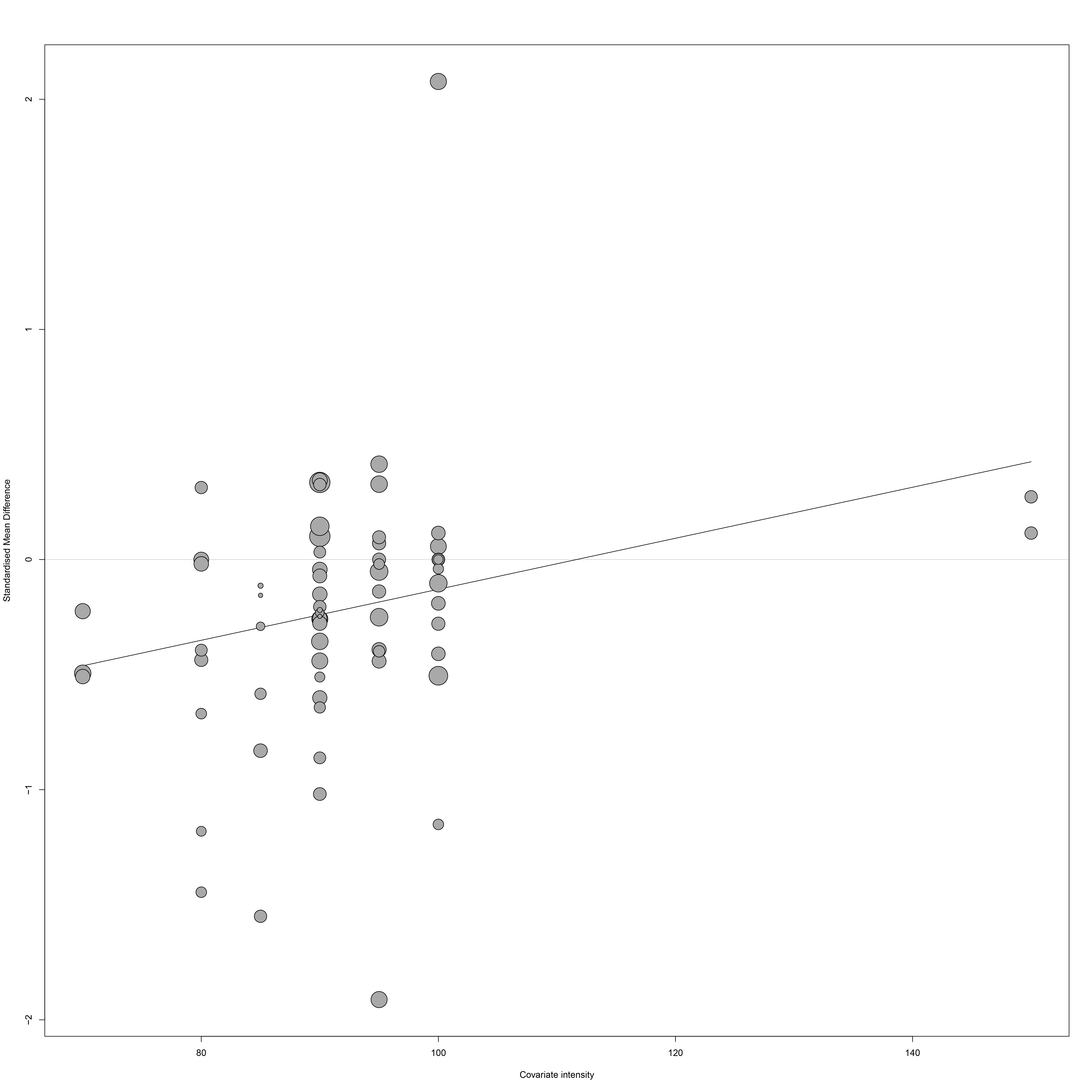
**

**E** Associations between HIIT training intensity and cfPWV. r=0.0001，*p*=0.05

**Appendix 11.** The funnel plot graphics of PLT, FIB, cfPWV, AIx, AIx@75HR, and FMD in MA.


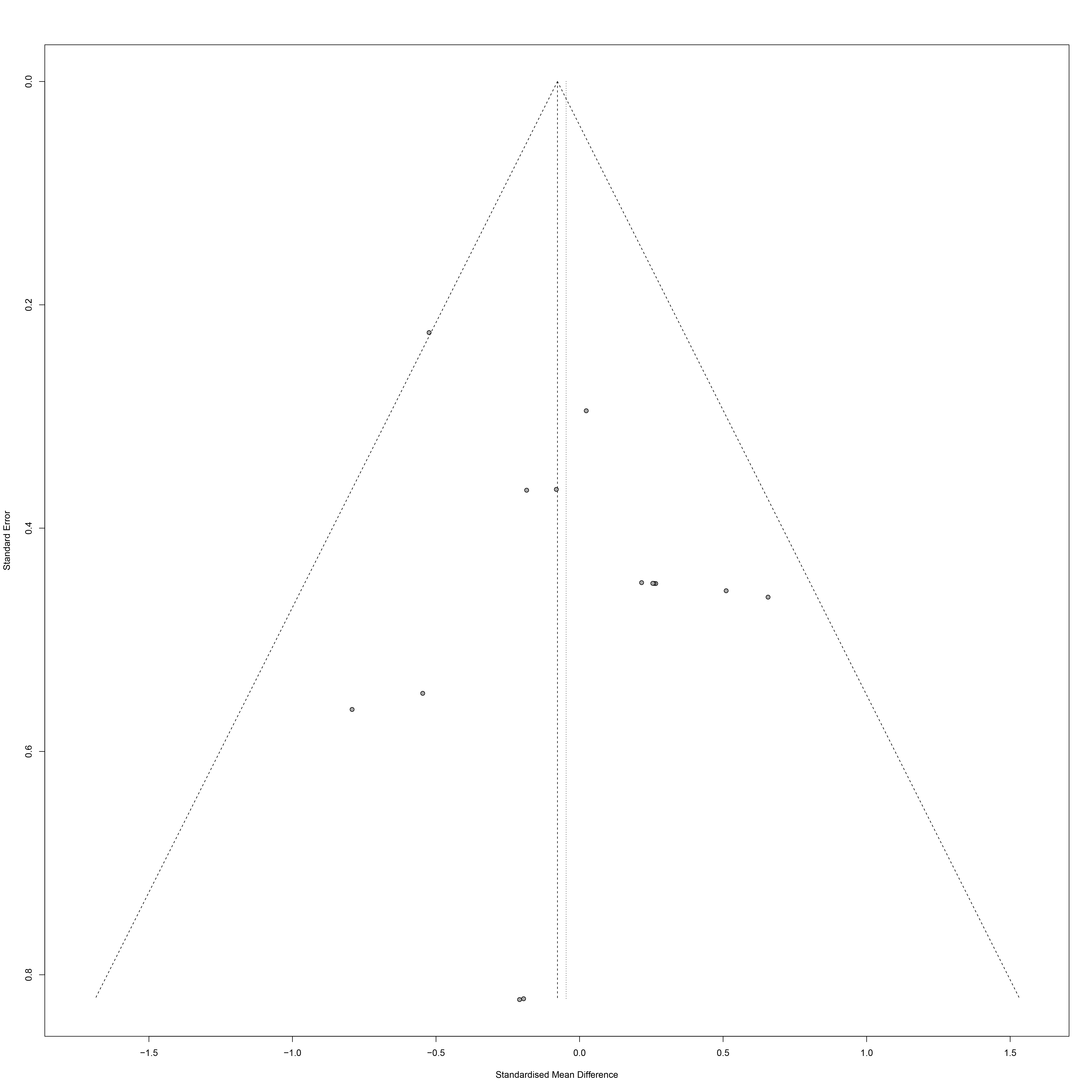


**A** PLT Egger *p=*0.27


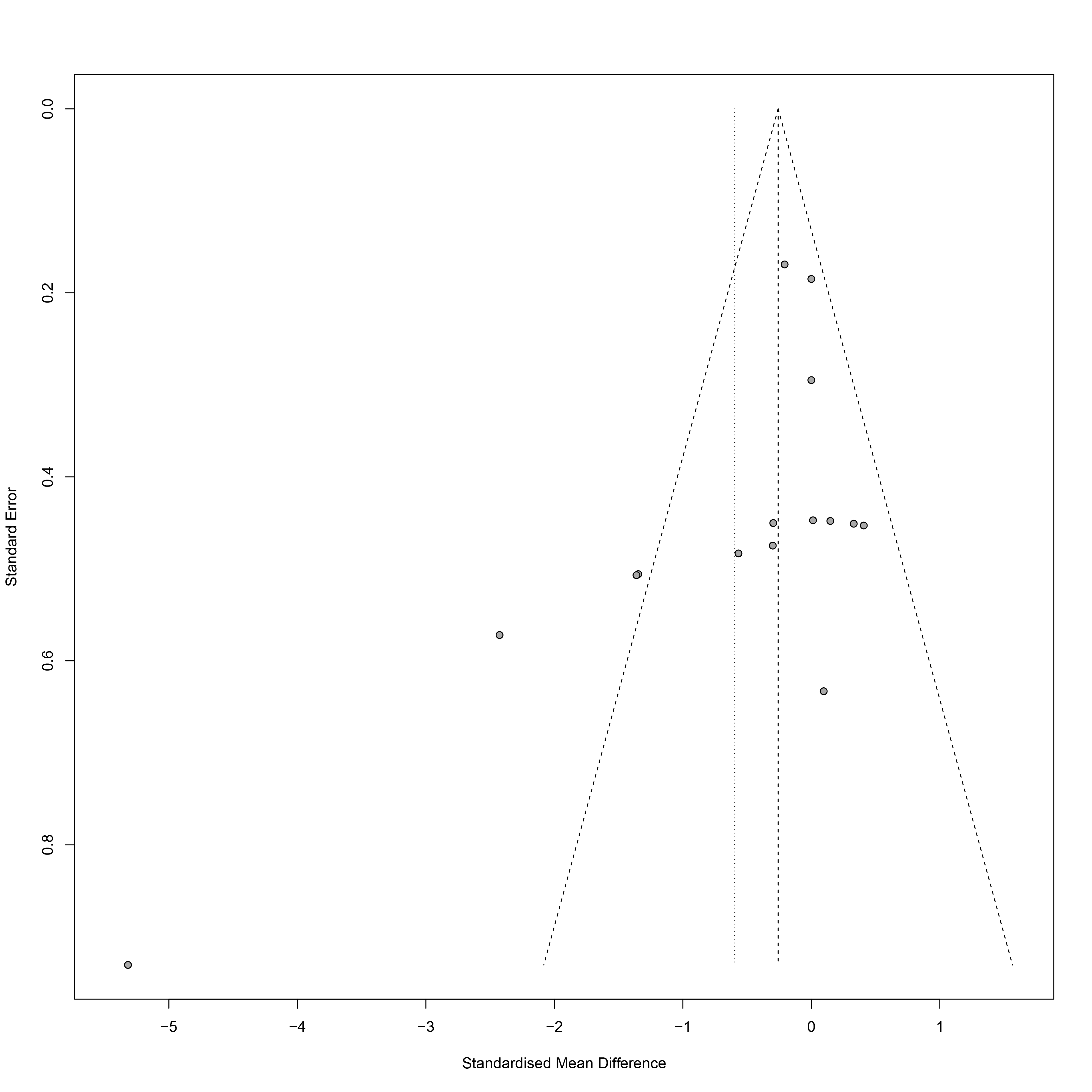


**B** FIB Egger *p=*0.06


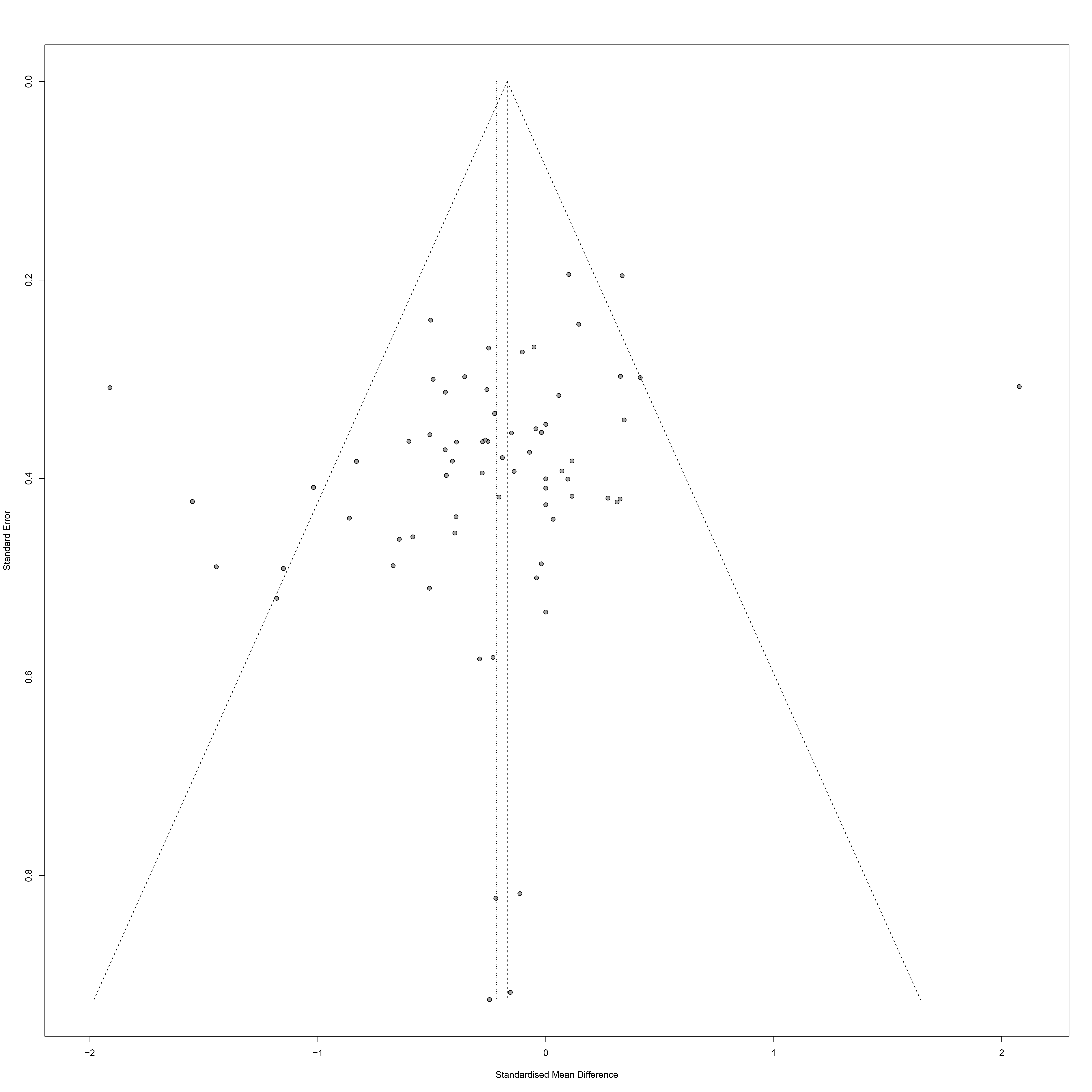


**C** cfPWV Egger *p=*0.04


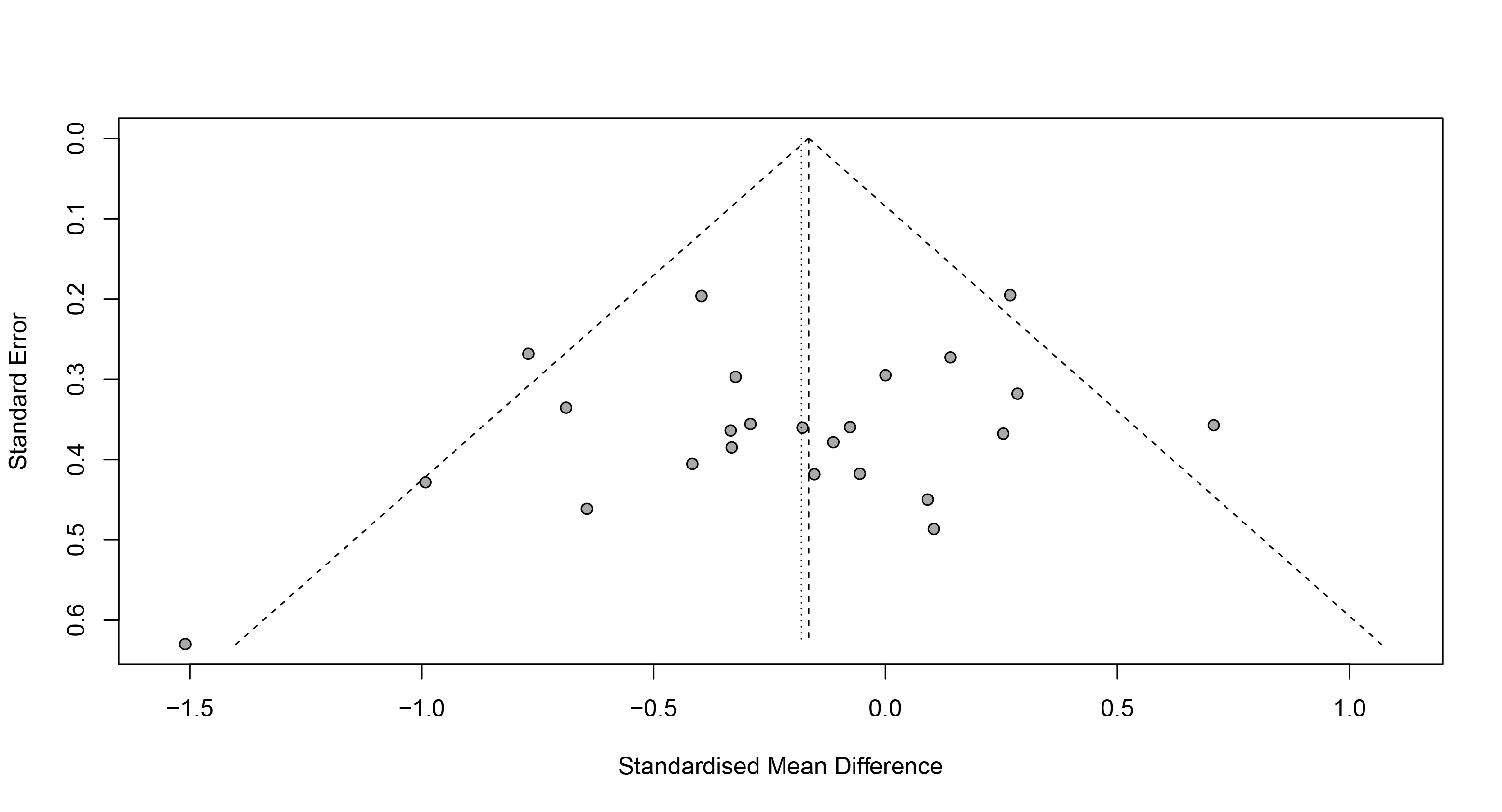


**D** AIx Egger *p=*0.29


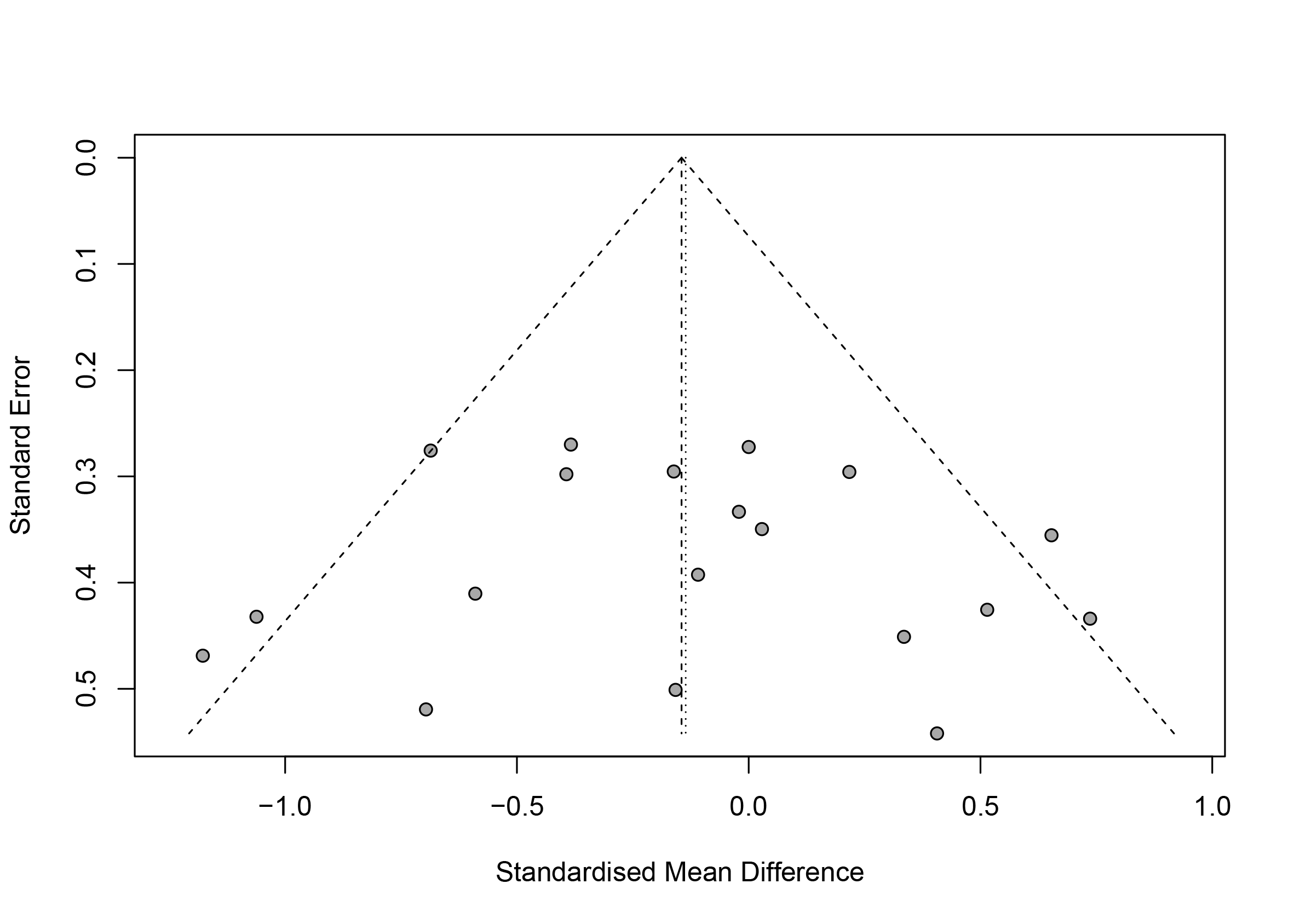


**E** AIx@75HR Egger *p=*0.34


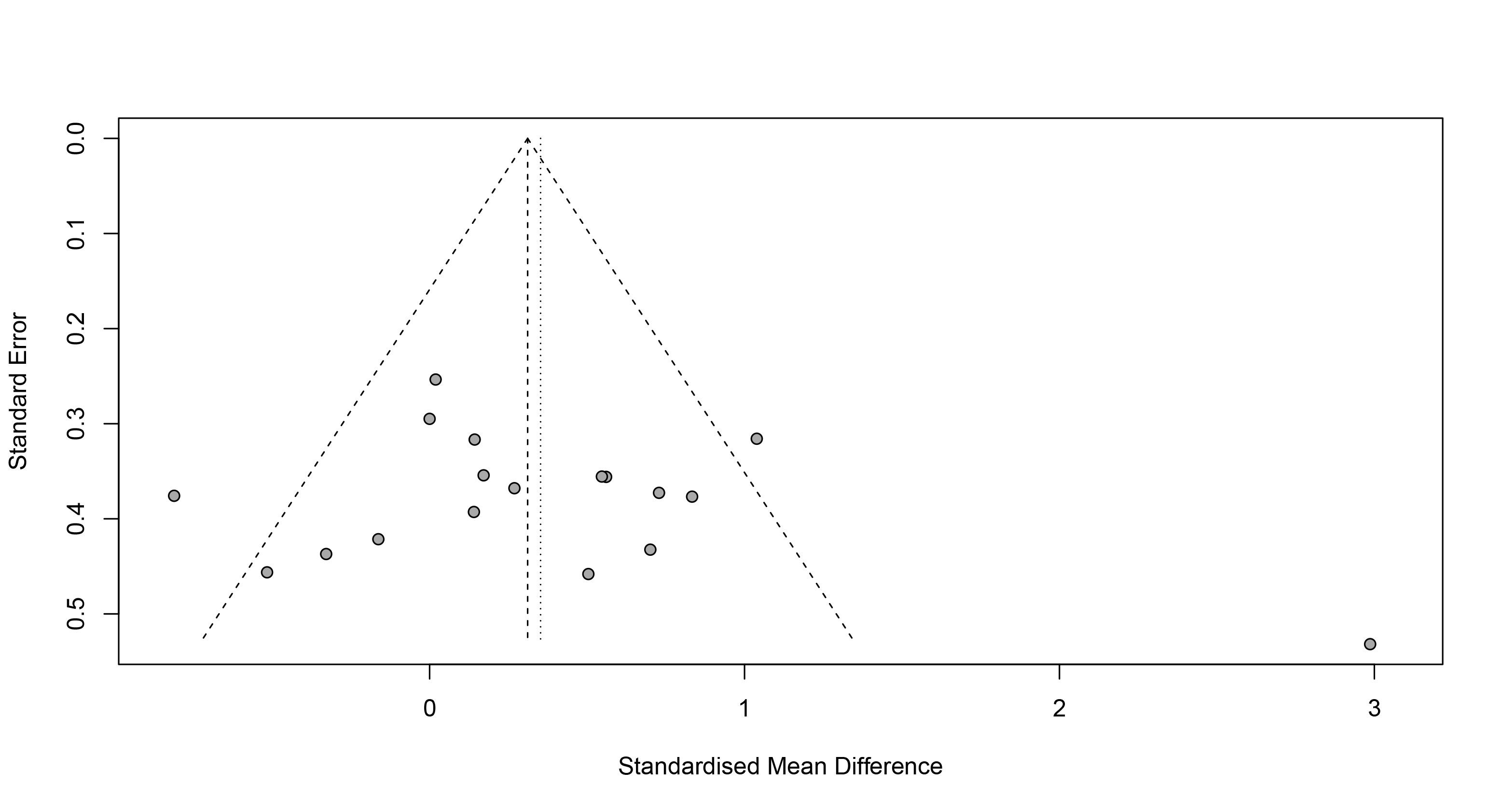


**F** cfPWV Egger *p=*0.29


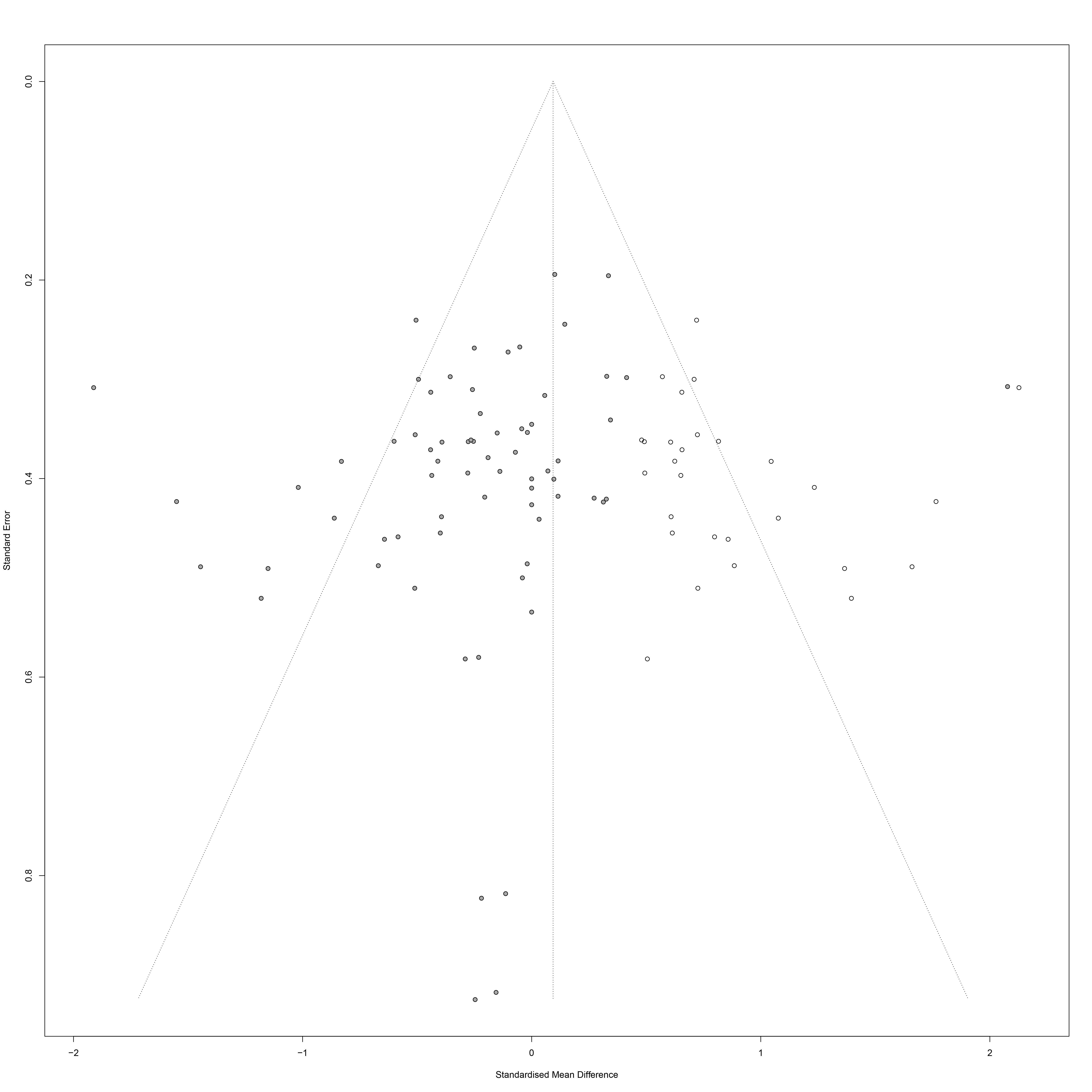


**G** Trim-and-Fill Adjusted Funnel Plot in cfPWV Meta-Analysis
